# *Porphyromonas gingivalis* Type IX Secretion Substrates Are Cleaved and Modified by a Sortase-Like Mechanism

**DOI:** 10.1371/journal.ppat.1005152

**Published:** 2015-09-04

**Authors:** Dhana G. Gorasia, Paul D. Veith, Dina Chen, Christine A. Seers, Helen A. Mitchell, Yu-Yen Chen, Michelle D. Glew, Stuart G. Dashper, Eric C. Reynolds

**Affiliations:** Oral Health Cooperative Research Centre, Melbourne Dental School, and The Bio21 Institute, The University of Melbourne, Victoria, Australia; University of Alberta, CANADA

## Abstract

The type IX secretion system (T9SS) of *Porphyromonas gingivalis* secretes proteins possessing a conserved C-terminal domain (CTD) to the cell surface. The C-terminal signal is essential for these proteins to translocate across the outer membrane via the T9SS. On the surface the CTD of these proteins is cleaved prior to extensive glycosylation. It is believed that the modification on these CTD proteins is anionic lipopolysaccharide (A-LPS), which enables the attachment of CTD proteins to the cell surface. However, the exact site of modification and the mechanism of attachment of CTD proteins to the cell surface are unknown. In this study we characterized two *wbaP (PG1964)* mutants that did not synthesise A-LPS and accumulated CTD proteins in the clarified culture fluid (CCF). The CTDs of the CTD proteins in the CCF were cleaved suggesting normal secretion, however, the CTD proteins were not glycosylated. Mass spectrometric analysis of CTD proteins purified from the CCF of the *wbaP* mutants revealed the presence of various peptide/amino acid modifications from the growth medium at the C-terminus of the mature CTD proteins. This suggested that modification occurs at the C-terminus of T9SS substrates in the wild type *P*. *gingivalis*. This was confirmed by analysis of CTD proteins from wild type, where a 648 Da linker was identified to be attached at the C-terminus of mature CTD proteins. Importantly, treatment with proteinase K released the 648 Da linker from the CTD proteins demonstrating a peptide bond between the C-terminus and the modification. Together, this is suggestive of a mechanism similar to sortase A for the cleavage and modification/attachment of CTD proteins in *P*. *gingivalis*. PG0026 has been recognized as the CTD signal peptidase and is now proposed to be the sortase-like protein in *P*. *gingivalis*. To our knowledge, this is the first biochemical evidence suggesting a sortase-like mechanism in Gram-negative bacteria.

## Author Summary

Chronic periodontitis, associated with the pathogen *Porphyromonas gingivalis*, is a major public health problem. *P*. *gingivalis* secretes virulence factors including the gingipains via the type IX secretion system (T9SS). These proteins contain a C-terminal signal that allows their secretion through the T9SS and it is cleaved by the protein PG0026 at the cell surface. Here we identify a mechanism by which gingipains and other proteins attach to the cell surface of this bacterium. We found that after removal of the C-terminal signal, the proteins were modified via a peptide linkage to either a putative component of anionic lipopolysaccharide (A-LPS) in the wild type or peptides from the growth medium in mutants lacking A-LPS, which has been suggested to anchor the proteins to the cell surface. Results from this study provide evidence for the exact site of modification of these proteins with A-LPS. Furthermore, our results also demonstrate for the first time that the anchoring mechanism of this secretion system involves a substitution between the signal and the anchor (A-LPS) which can be explained by the action of a single enzyme known as a sortase, which we believe is PG0026. This is the first evidence for a sortase-like mechanism in Gram-negative bacteria.

## Introduction

Chronic periodontitis, an inflammatory disease of the supporting tissues of the teeth is a major public health problem. *Porphyromonas gingivalis*, a Gram-negative, black-pigmented, anaerobic bacterium is regarded as a keystone pathogen for this disease [1]. Periodontitis has been associated with systemic conditions such as cardiovascular disease, dementia, rheumatoid arthritis, preterm birth and low birth weight [2, 3]. A diverse repertoire of *P*. *gingivalis* virulence factors such as lipopolysaccharide (LPS), fimbriae, capsular polysaccharide (CPS), haemagglutinin and cysteine proteases [Arg-gingipains (RgpA and RgpB) and Lys-gingipain (Kgp)] have been implicated in the pathogenesis of periodontitis [4]. Of these, the gingipains are considered the most important virulence factors and to date are the best studied [1, 5–7].

The gingipains belong to a group of proteins called CTD proteins [8]. There are approximately 30 CTD proteins in *P*. *gingivalis* and they all possess a conserved C-terminal domain [8]. Recently it has been demonstrated that the CTD- containing proteins are secreted and attached to the cell surface via the type IX secretion system (T9SS) [9–12]. These CTD proteins have also been found to be extensively modified and migrate as diffuse bands on SDS-PAGE with molecular weights at least 20 kDa higher than that predicted from their sequence [12, 13]. *P*. *gingivalis* has two different types of LPS, namely O-LPS and A-LPS. LPS consists of three major components: polysaccharide, core oligosaccharide and lipid A. The difference between O-LPS and A-LPS is in the repeating polysaccharide. The O-polysaccharide consists of a tetrasaccharide repeat unit composed of [→6)-alpha-D-Glc*p*-(1→4)-alpha-L-Rha*p*-(1→3)-beta-D-GalNAc-(1→3)-alpha-D-Gal*p*-(1→] [14] whilst the polysaccharide of A-LPS consists of repeating units of a backbone of α(1–6)-linked mannose residues and side chains of α-1,2-linked oligosaccharide of different lengths. One of the side chains consists of mannose phosphate [15, 16]. A monoclonal antibody (mAb 1B5) reactive to Manα1-2Man α1-phosphate side chains of A-LPS was produced by Curtis *et al*. [17]. The extensively modified CTD proteins react with mAb 1B5 antibodies suggesting that the modification on CTD proteins is A-LPS [17]. The presence of lipid A in the CTD proteins however, has not yet been confirmed. The A-LPS modification is thought to be the anchor that tethers CTD proteins to the cell surface [18], however, the mechanism of attachment is unknown.

Recently, we showed that the CTDs of CTD proteins are cleaved by PG0026 (PorU) during secretion in *P*. *gingivalis* and are released in the culture fluid [19]. Additionally, it was found that mutation of cysteine at position 690 to alanine abolished PG0026 cleavage activity [19]. Furthermore, it was also revealed that the CTD itself is not the site of modification but rather functions as the secretion signal of the T9SS with the modification occuring at or near the C-terminus of the mature CTD proteins [19]. Moreover, it has also been demonstrated that CTD cleavage, extensive post-translational modification and membrane localization is a conserved feature of the T9SS in *Parabacteroides distasonis*, *Prevotella intermedia*, *Tannerella forsythia* and *Cytophaga hutchinsonii* [12].

A number of proteins have been identified to play a role in the secretion and modification of CTD proteins in *P*. *gingivalis*. Insertional inacivation of *porT*, *porV* (PG27, *lptO*), *porK*, *porL*, *porM*, *porN*, *porP*, *porU* (PG0026), *porQ*, *porW* and *sov* results in defective secretion, and these mutants show accumulation of CTD proteins in the periplasm and exhibit reduced cell surface and culture supernatant gingipain activity [9, 19–23]. Conversely, mutants that are defective in the production of A-LPS display no cell surface gingipain activity as the proteins are found in the culture supernatant [16, 18, 24–30]. To date, several mutant strains have been identified that are defective in the modification of CTD proteins and do not produce mAb 1B5 reactive bands in western blots. Wzy, WaaL, GtfB and WzzP proteins are required for the synthesis of both O-LPS and A-LPS [16, 24–26] while gene products of *porR*, *vimA*, *vimE*, *wbpB*, *ugdA* and *wbaP* appear to abolish the synthesis of A-LPS only [18, 24, 27–30].

Although numerous proteins have been identified to play a role in the synthesis of A-LPS, no study has been performed to investigate the effect of the lack of A-LPS on the processing of CTD proteins. As previous studies on WbaP have focused on the effects of WbaP on the synthesis of O-LPS and A-LPS [24, 30], in this study, we aimed to understand the role of WbaP in the secretion and modification of CTD proteins in *P*. *gingivalis wbaP* mutants. We found that in *wbaP* mutants lacking A-LPS, the CTD was cleaved and the mature CTD proteins were modified at the C-terminus with peptides/amino acids from the growth medium while in the wild type, the CTD proteins were modified at the C-terminus with a putative component of A-LPS via a peptide bond. This transpeptidation suggests that modification and surface attachment of T9SS substrates in the Gram-negative bacterium *P*. *gingivalis* occurs via a sortase-like mechanism.

## Materials and Methods

### Bacteria and culture conditions

*Porphyromonas gingivalis* strains were grown in tryptic soy enriched Brain Heart Infusion broth (TSBHI) (25 g/L Tryptic soy, 30 g/L BHI) supplemented with 0.5 mg/mL cysteine, 5 μg/mL haemin and 5 μg/mL menadione [21]. A colony from the *P*. *gingivalis* W50 *rgpA* ^-^ *rgpB* ^-^ *kgp* ^-^ triple mutant (W50ABK) [31] was isolated and grown in the same media as above with appropriate antibiotic selections renamed in this paper to W50ABK*WbaP. All *P*. *gingivalis* strains were grown anaerobically (80% N_2_, 10% H_2_ and 10% CO_2_) at 37°C to an O.D_600nm_ of 0.4–0.5. A new *rgpA* ^-^ *rgpB* ^-^ *kgp* ^-^ triple mutant strain, ECR353 was produced as follows. Plasmid pNS1 containing a fragment of *kgp* [32] was transformed into the *dam* ^-^ *dcm* ^-^ *E*. *coli* strain SCS110, isolated, then digested with Bc1I and SnaBI. The *ermFermAM* genes were excised from pVA2198 [33] using EcoICRI and BamHI digestion and ligated to the digested pNS1 producing pNS1E1. Plasmid pNS1E1 was linearised using XbaI and used to transform the *rgpA*, *rgpB* double mutant *P*. *gingivalis* W50AB [13]. Recombinant cells were selected using HBA supplemented with erythromycin and correct homologous recombination was verified using PCR. For glycine treated *P*. *gingivalis* strains, W50ABK*WbaP, W50WbaP and W50 were grown anaerobically overnight in half strength TSBHI media in the absence or presence of 0.2 M glycine.

### Construction of W50WbaP deletion mutant

The *wbaP* deletion mutant in the W50 strain was generated by double cross-over recombination using the suicide plasmid pΔWbaP-ermF that contained upstream and downstream regions of the *wbaP* (PG1964) gene that were directionally cloned adjacent to an erythromycin resistance cassette (*ermF*). The upstream and downstream regions of *wbaP* were amplified by PCR from W50 genomic DNA using the following primer pairs: Upstream-F (5’ACGCCCATGGGGAGTTGGCCTGGG ATCTGATCG 3’) (NcoI site underlined)/ Upstream-R (5’ACGCCCGCGGACCCACAGG CTCTTGGCGTTGAA 3’) (SacII site underlined) and Downstream-F (5’ACGCCTGCAGCCG GATGAAATGCTGGAACGCCT 3’) (PstI site underlined)/ Downstream-R (5’ACGCATGCAT GAAGATCCCCGAAATGGCACCTG 3’) (NsiI site underlined). The PCR fragments were sequentially cloned into the suicide vector, pAL30 [34] and the resulting plasmid pΔWbaP-ermF was linearised using NsiI and electroporated into *P*. *gingivalis* W50. The correct homologous recombination of transformants was verified using PCR.

### Preparation of vesicle free culture fluid (CCF)

Vesicle free culture fluid (CCF) was essentially obtained as previously published [19]. Briefly, *P*. *gingivalis* cultures were harvested at an early log phase, centrifuged at 8000 *g* for 25 min and the supernatant filtered through a 0.2 μm filter. Filtered supernatant was centrifuged at 180000 *g* for 45 min to pellet the vesicles and supernatant was collected and designated CCF.

### Preparation of periplasm and cell envelope

Periplasmic fractions were prepared by cold-osmotic shock essentially as described previously [21]. Briefly, *P*. *gingivalis* 250 mL cell cultures were centrifuged at 8000 *g* for 25 min and the cell pellet was resuspended in a solution containing 500 mM sucrose, 30 mM Tris-HCl, pH 7.4, 1 mM EDTA, pH 8.0, 2 mM Tosyl-l-lysyl-chloromethane hydrochloride (TLCK) (Sigma-Aldrich) and complete protease inhibitor (CIP) cocktail (Roche). The cells were incubated in ice for 10 min and then centrifuged at 8000 *g* for 25 min. The supernatant was removed and the cell pellet was resuspended in 5 mM MgCl_2_ containing TLCK and CIP and gently mixed for 10 min. The cells were centrifuged again and the supernatant collected as the periplasmic fraction.

The cell envelope (CE) was prepared by sonicating washed *P*. *gingivalis* cells in phosphate buffer saline (PBS) using an ultrasonic processor (model CPX 750, Cole Parmer) with amplitude seat to 40%, pulser to 1 sec on, 2 sec off for a total of 30 min. The membranes were pelleted by centrifugation at 48400 *g* for 20 minutes, and washed with PBS.

### SDS-polyacrylamide gel electrophoresis

Protein samples were precipitated using 10% trichloroacetic acid (TCA) and washed with ice cold acetone and allowed to dry. Samples were then resuspended in 7 M urea, 2 M thiourea, 4% CHAPS, 30 mM Tris-HCL, pH 8. An aliquot of sample was mixed with SDS- loading dye and analysed on NuPAGE Bis-Tris precast gels. The gels were electrophoresed using either MOPS or MES SDS running buffer (Life Technologies). The gels were stained overnight with 0.1% w/v Coomassie Blue G-250 in 17% w/v ammonium sulphate, 34% v/v methanol and 3% v/v o-phosphoric acid.

### In gel tryptic digestion and mass spectrometry

Protein gel bands were excised and washed in 50 mM NH_4_HCO_3_/ethanol 1:1 solution, and reduced and alkylated with dithiothreitol (DTT) and iodoacetamide respectively. In gel tryptic digestion was performed overnight at 37°C using sequencing grade modified trypsin (10 ng/μL) (Promega, NSW, Australia). The resulting peptides were analysed as previously described using an LC-MS/MS system comprising an Ultimate 3000 HPLC (Dionex, Sydney, Australia) and an Esquire HCT Ultra ion trap [35].

### 2-dimensional gel electrophoresis (2-DGE)

Proteins from the CCF of W50ABK*WbaP and W50WbaP were precipitated using 10% TCA. The sample was then solubilised in a buffer containing 7 M Urea, 2 M thiourea, 4% CHAPS, 40 mM DTT, 0.2% ampholytes and 1% bromophenol blue. The IPG strip (pH 3–10) (Bio-Rad) was rehydrated overnight with the solubilised proteins from the CCF at room temperature. After rehydration the IPG strips were assembled in the IPGRunner Mini-Cell and isoelectric focusing (IEF) was performed with the following settings (175 V, 15 min, 175–2000 V ramp for 45 min, 2000 V for 120 min). Following IEF, the strip was reduced and alkylated using 10 mg/mL DTT and 25 mg/mL iodoacetamide, respectively. The IPG strip was then set by 0.5% agarose in running buffer on top of a ZOOM gel (Life Technologies) and SDS-PAGE was performed at 200 V for 50 min. The gel was then stained with Coomassie blue.

### Automated protein spot picking, digestion and identification by MALDI TOF/TOF

Protein spots were picked with a robot, digested with trypsin and analysed with MALDI-TOF/TOF mass spectrometry as previously described [36] except that the gel spots were first dehydrated with 100% acetonitrile followed by 80% acetonitrile in water to avoid over dried gel pieces from “flying” out of the plate and 0.1% n-octyl β-D-glucopyranoside was added to the trypsin solution to aid the transfer of the small volumes of trypsin from the pipette tip to the plate.

### Western blot

Protein samples were resolved on SDS-PAGE gels and transferred onto a nitrocellulose membrane. The membrane was blocked with 5% skim milk powder in PBS-tween (0.05% (v/v) tween-20 in PBS). The membranes were probed with mAb 1B5 (a kind gift from Professor M.A Curtis) primary antibody followed by goat anti-mouse HRP conjugated secondary antibody. The signal was developed using SuperSignal West Pico chemiluminescent substrate and visualized with a LAS-3000 imaging system.

### Whole genome sequencing of W50ABK*WbaP

*P*. *gingivalis* W50ABK*WbaP was grown to stationary phase in supplemented Brain Heart Infusion medium, harvested by centrifugation and washed in 10 mM Tris-HCl pH 8.0. DNA was extracted using the Blood and Tissue Genomic DNA Isolation Kit (Qiagen) and concentrated to 100 ng/μL using ethanol precipitation then resuspended in 10 mM Tris-HCl pH 8.0.

Whole-genome resequencing was performed at The Australian Genome Research Facility (AGRF) using the Illumina HiSeq-2000 Platform with the CASAVA1.8.2 pipeline. Sequencing generated 8,132,274 paired-end 100 bp reads, or 1.6 GB of nucleotides in total, representing approximately 600-fold coverage of the 2.3 MB genome. Average insert size was calculated as 422 bp. To identify small indels and single-nucleotide variants, sequence reads were aligned against the *P*. *gingivalis* W83 genome (NC_002950.2), modified with the insertion of *cat*, *tetQ* and *erm* inactivating *rgpA*, *rgpB* and *kgp* respectively [31]. Alignments were performed using the BWA alignment tool [37], SAMtools [38] and Freebayes on the Galaxy platform [39–42].

### Preparation of *P*. *gingivalis* LPS and silver staining

*P*. *gingivalis* cells were washed once in water and suspended in 10 mM Tris (pH 8.0) containing 2% SDS and 10 mM DTT. The bacteria were heated at 100°C for 5 minutes and cooled to room temperature prior to addition of 20 μg/mL Benzonase (Sigma-Aldrich). The samples were then incubated at 37°C for 1 hour followed by the addition of 20 μg/mL of proteinase K and further incubation at 37°C overnight. The next day, gel loading buffer was added to the samples and subjected to SDS-PAGE followed by silver staining using the tetrathionate method as previously described [43, 44].

### Cryo-electron microscopy

Cryo-EM on W50WbaP, ECR353 and wild type *P*. *gingivalis* was performed as described by Chen *et al*. [21].

### Purification of Pro-CPG70, P59 and P27

*P*. *gingivalis* W50ABK*WbaP was grown in the presence or absence of 0.2 M glycine. The culture was spun at 8000 g for 20 minutes and the supernatant (culture fluid) was transferred to a new tube. The culture fluid was filtered using 0.22 μm filter and concentrated using a Sartocon Mini cross-flow filtration device (Sartorius,) equipped with a 3-kDa molecular mass cut-off filter cassette. The concentrated sample was applied onto a MonoQ column (GE Healthcare); the bound proteins were eluted with a 40-mL linear gradient of 50–500 mM NaCl in 20 mM Tris-HCL, pH 8 using an ÄKTAexplorer 100 automated liquid chromatography system (GE Healthcare). The column fractions that contained Pro-CPG70 were pooled and concentrated using an Amicon Centriprep YM-10 centrifugal filter device (Millipore). The concentrate was applied onto a S200 gel filtration column (GE Healthcare) equilibrated with a buffer containing 20 mM Tris-HCL, pH 8 and 150 mM NaCl and 0.5 mL column fractions were collected at a flow rate of 0.5 mL/min.

### Electrospray ionisation-time of flight (ESI-TOF) mass spectrometry

ESI-TOF mass spectrometry was performed on the purified Pro-CPG70, P59 and P27 samples using an Agilent 6220 Q-TOF coupled to an Agilent 1200 HPLC. Separation of sample was performed on a C_4_ column (2.1 mm × 150 mm, 3.6-μm particle size) with 0.1% formic acid aqueous solution as buffer A and acetonitrile + 0.1% formic acid as buffer B. The gradient was 5–95% for 8 minutes. The mass spectrometer was operated in positive MS only mode and data collected from 100 to 2500 m/z. Internal reference masses of 121.0508 and 922.0097 were used for calibration. Deconvolution of the mass spectra was carried out using Agilent Mass Hunter Qualitative Analysis software (B.05) using the maximum entropy algorithm.

### In solution digestion and LTQ Orbitrap mass spectrometery

Purified Pro-CPG70 and P59 were TCA precipitated and the pellet was resuspended in 8M urea containing 20 mM DTT. After 2 hours of incubation at room temperature, 55 mM of iodoacetamide was added to the reaction and incubated for 30 minutes in the dark. Trypsin (3 μg) was added and the samples were incubated overnight at 37°C with shaking. Following incubation, the samples were desalted using C18 zip-tip, and the eluted samples were freeze-dried to reduce the volume and were analysed with an LTQ Orbitrap Elite mass spectrometer (Thermo Scientific) with a nanoESI source interfaced with an Ultimate 3000 RSLC nano-HPLC (Thermo Scientific). The LC system was fitted with an Acclaim Pepmap nano-trap column (Dionex-C18, 100 Å, 75 μm x 2 cm) and an Acclaim Pepmap RSLC analytical column (Dionex-C18, 100 Å, 75 μm x 15 cm). The tryptic peptides were injected into the enrichment column at an isocratic flow of 5 μL/min of 3% v/v CH_3_CN containing 0.1% v/v formic acid for 5 min applied before the enrichment column was switched in-line with the analytical column. The eluents were 0.1% v/v formic acid (solvent A) and 100% v/v CH_3_CN in 0.1% formic acid (solvent B). Separation was performed with a solvent B gradient of 6–80% for 53 minutes. The mass spectrometer was operated in the data-dependent mode with nano-ESI spray voltage of 2.0kV, capillary temperature of 250°C and S-lens RF value of 55%. All spectra were acquired in positive mode with full scan MS spectra scanning from m/z 300–1650 in the FT mode at 240,000 resolution after accumulating to a target value of 1.0e^6^. The top 10 most intense precursor ions were subjected to high energy collision induced dissociation (HCD) with normalized collision energy of 35 and activation time of 0.1 ms. Dynamic exclusion with 2 repeat counts over 30 seconds and exclusion for 70 seconds was applied. The Mascot MS/MS ions search was performed using the following settings: *P*. *gingivalis* W83 database, trypsin, 2 missed cleavages, carbamidomethyl-Cys as a fixed modification and oxidation of methionine as a variable modification, 10 ppm peptide tolerance, 0.2 Da MS/MS tolerance and “Decoy” was enabled.

Manual MS/MS searching was done by extracting ion chromatograms for the major b-ions expected. For the CPG70 C-terminal peptide (KAEDYIEVILDD…), these ions were m/z 948.5 (b-8 ion) and m/z 1061.5 (b-9 ion). Parent ions were considered to correspond to modified C-terminal peptides when these ions were the major fragment ions in the MS/MS spectrum, and upon inspection several additional neighbouring b-ions were present. The peptide modifications were assigned by calculating the accurate delta mass, adding the mass of water (18.010565) and searching the Metlin database (http://metlin.scripps.edu/metabo_search_alt2.php) for matching metabolites [45]. The settings used were tolerance = 15 ppm, charge = neutral, remove peptides from search = unchecked.

### Purification of modified CTD proteins from *P*. *gingivalis*, W50

Modified CTD proteins were partially purified from *P*. *gingivalis* wild type W50 6-day old cultures as previously published for the purification of modified Arg gingipain (“RIB”) [46] with the exception that only a single chromatographic step was used. Proteins solubilised in 1% Zwittergent were separated by anion exchange chromatography using a Q-Sepharose column equilibrated in 20 mM Bis-Tris/5 mM CaCl_2_/50 mM NaCl/0.05% Zwittergent, pH 6 and eluted with a linear gradient of 50–500 mM NaCl.

### Deglycosylation of CTD proteins and digestion with trypsin and proteinase K

Fractions containing modified CTD proteins were combined and concentrated using a 10 kDa molecular weight cut off membrane. The proteins were precipitated using 10% TCA and the pellet was resuspended in 50% acetonitrile/0.1% aqueous trifluoroacetic acid. The samples were transferred to glass vials and freeze dried thoroughly. Deglycosylation was performed using the protocol from the manufacturer of PROzyme/Glyko Glycofree Chemical deglycosylation kit (GKK-500). The deglycosylated CTD proteins were analysed by SDS-PAGE. In-gel digestion on the protein bands was performed with trypsin as described above. Proteinase K digestion (1 μg per band) was performed on the tryptic fragments at 45°C for 4 hours. The tryptic digests and proteinase K digested samples were analysed by LC-MS-MS as described above.

## Results

### The colony isolate of W50ABK contains a spontaneous mutation within the *wbaP* gene

**Fig 1:**
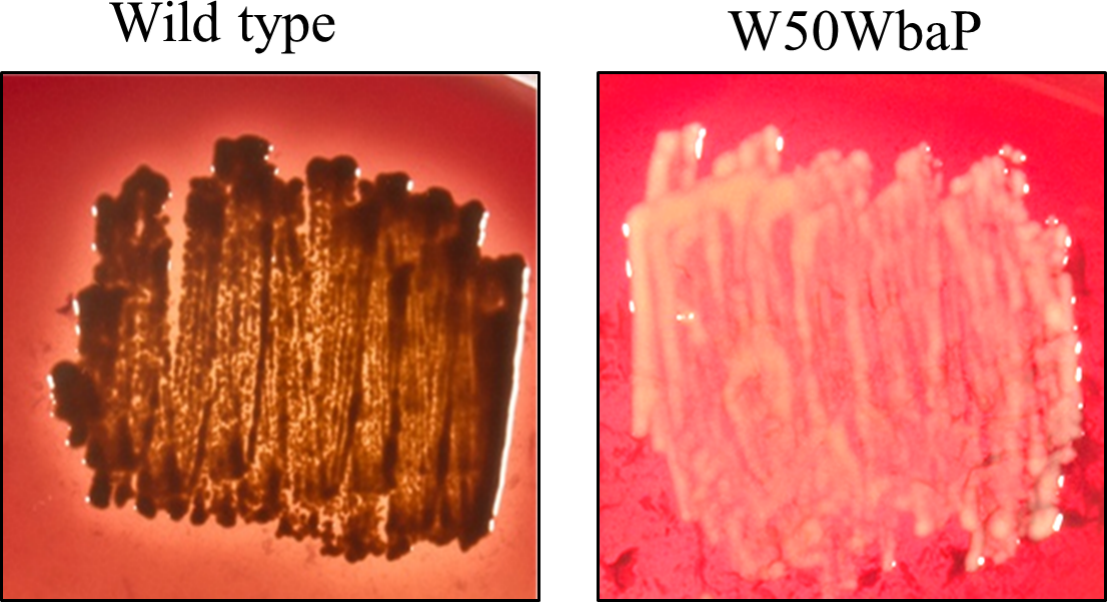
No pigmentation of W50WbaP mutant. Pigmentation of WT and W50WbaP mutant on blood agar plates.

*P*. *gingivalis* W50ABK, lacking gingipains RgpA, RgpB and Kgp, was originally generated in our laboratory over a decade ago. Recently we analysed a colony isolate of the W50ABK mutant together with the wild type, W50, using proteomics and western blots. The mutant had a considerably reduced number of CTD proteins on the cell surface compared to wild type and was found to be defective in A-LPS synthesis (see below). As the absence of gingipains was not expected to affect the synthesis of A-LPS it was speculated that a spontaneous mutation had occurred in the W50ABK clonal isolate and therefore the whole genome was sequenced to identify the mutation. The sequencing revealed two point mutations. There was a G>T transversion relative to position 2,053,626 of the *P*. *gingivalis* W83 genome that changes a GAG (Glu) codon to a TAG stop codon in *wbaP*, which encodes a sugar transferase [30]. The creation of a stop codon at this position would result in truncation of the 498 residue WbaP to 192 residues. This mutation was found in 100% of reads at the particular position, with a good level of coverage (>200), and did not occur in any other *P*. *gingivalis* W50 genomes previously sequenced in this laboratory. There was also a non-synonymous mutation G>C relative to position 2,333,645 of the *P*. *gingivalis* W83 genome that changes a GCA (Ala) codon to a CCA (Pro) codon in PG2219, *trkH* that encodes a potassium transporter resulting in an amino acid substitution at position 188 of the TrkH protein. This mutation was also found to be present in the genome of our W50 laboratory strain used to create the W50ABK strain, but was not present in W50 isolates obtained from external sources including the ATCC 53978 strain. The clonal isolate W50ABK was therefore renamed W50ABK*WbaP. To confirm that the lack of A-LPS in W50ABK*WbaP was due to a defective *wbaP* gene, we generated a *wbaP* deletion mutant in *P*. *gingivalis* W50 cells. Wild type, W50, *P*. *gingivalis* is black pigmented when grown on blood agar due to the accumulation of the μ-oxo bisheme complex of Fe(III) protoporphyrin IX [47]. In order to acquire this μ-oxo bisheme complex, Kgp on the cell surface is required to bind and hydrolyse the haemoglobin [48]. The *wbaP* mutant cells had no pigmentation, consistent with the intended mutation, as lack of A-LPS would result in no attachment of gingipains to the cell surface (Fig 1).

### Loss of CTD proteins in membrane fractions of WbaP mutants

**Fig 2:**
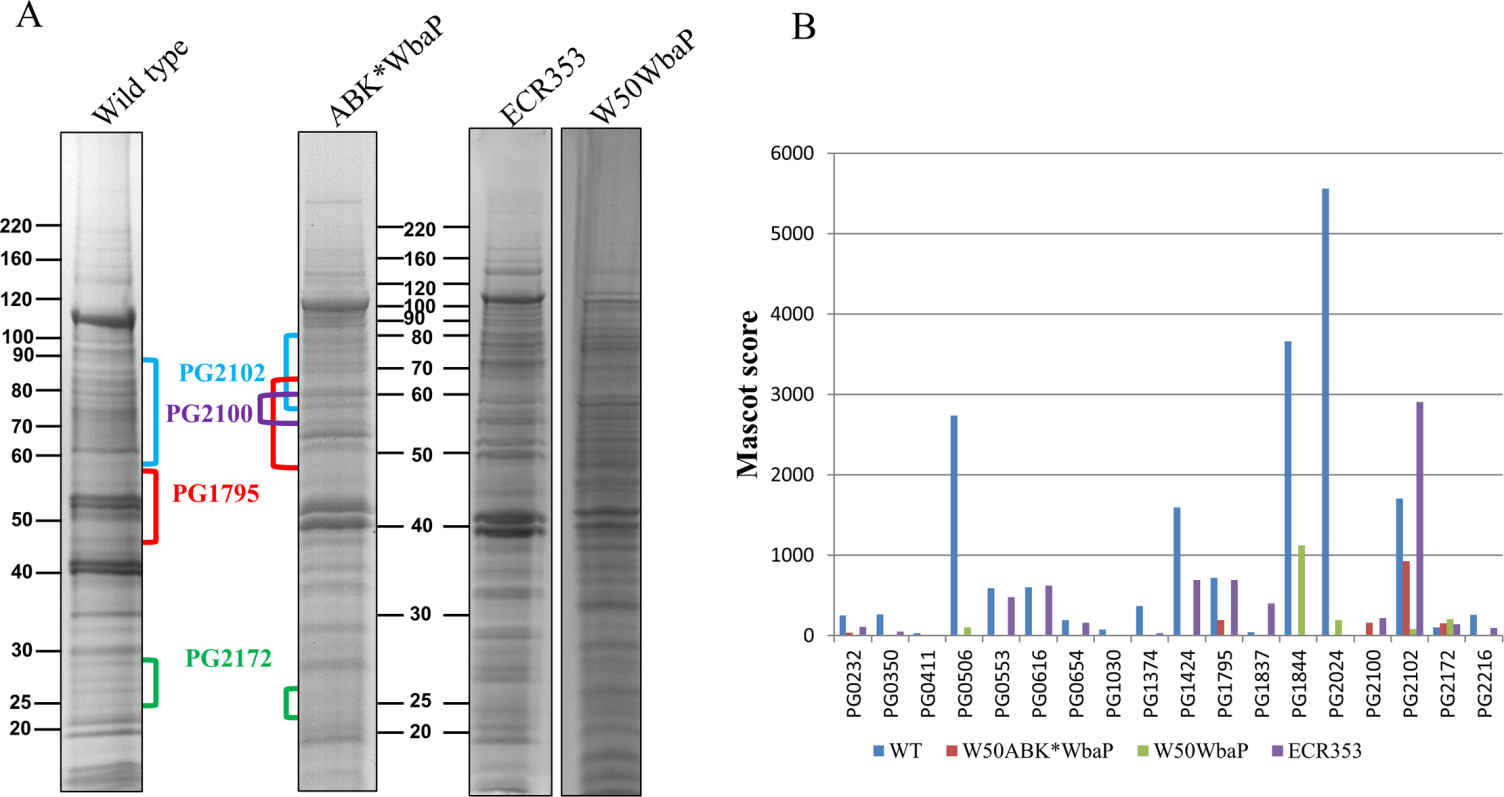
Cell envelope analysis of wild type and W50ABK*WbaP mutant. (A) CE proteins from WT (W50), W50ABK*WbaP, W50WbaP and ECR353 (*rgpA* ^-^ *rgpB* ^-^ *kgp* ^-^) were separated on SDS-PAGE. The gel was cut into segments and the proteins were identified by mass spectrometry. (B) A plot of Mascot scores of identified CTD proteins in the CE from WT, W50ABK*WbaP, W50WbaP and ECR353. When the same protein was identified from multiple gel segments the Mascot scores were summed (S1 Table).

The cell envelope (CE), containing both inner and outer membranes, was prepared from wild type, ECR353, W50WbaP and W50ABK*WbaP *P*. *gingivalis* cells by sonication. The proteins in the CE were TCA precipitated to deactivate proteases and subjected to SDS-PAGE separation (Fig 2A). The protein bands were identified by mass spectrometry (S1 Table). A total of 17 CTD proteins were identified in the wild type, but surprisingly only 5 CTD proteins were identified in the W50ABK*WbaP mutant (Fig 2B). Of the five identified proteins in the W50ABK*WbaP mutant, three were also identified in the wild type with a higher Mascot score and one (PG2172) with a lower Mascot score (Fig 2B). An exception was PG2100 which was only identified in the W50ABK*WbaP mutant (Fig 2B). All together, the results indicated a considerable reduction of CTD proteins on the cell surface of the W50ABK*WbaP mutant suggesting a defect in either the secretion or cell surface attachment of these proteins. In the wild type, the CTD proteins were found to be at elevated molecular weight and their CTD signals had been removed as previously published [12]. In W50ABK*WbaP, PG2102 and PG1795 were also found at an elevated molecular weight and apparently without their CTDs consistent with normal processing, however PG2100 and PG2172 appeared unmodified and their CTDs were present (Fig 2A). The W50WbaP mutant and ECR353 (another ABK mutant in W50) were analysed in the same way. W50WbaP exhibited a reduced number of CTD proteins in the CE sample, whilst a total of 13 CTD proteins were identified in the CE of ECR353 (Fig 2A), demonstrating that the reduction in the level of membrane-associated CTD proteins was due to the lack of WbaP rather than the gingipains.

### No accumulation of CTD proteins in the periplasm of W50ABK*WbaP

The periplasm of the W50ABK*WbaP mutant was analysed to determine if the CTD proteins were accumulating in the periplasm of this strain. The periplasmic fractions were obtained from the wild type and W50ABK*WbaP mutant by osmotic shock and were analysed by SDS-PAGE. The protein profile on the gel was comparable between the wild type and the W50ABK*WbaP mutant as opposed to secretion mutants such as *lptO* in which the CTD proteins accumulate in the periplasm [21] (S1 Fig). This suggested that there was no accumulation of CTD proteins in the periplasm of the W50ABK*WbaP mutant cells. Additionally, the proteins from the major bands in the periplasm of the W50ABK*WbaP mutant were identified by mass spectrometry to be *bona-fide* periplasmic proteins (S1 Fig) supporting the conclusion of non-accumulation of CTD proteins in the periplasm.

### Lack of A-LPS in W50ABK*WbaP and W50WbaP mutants

**Fig 3:**
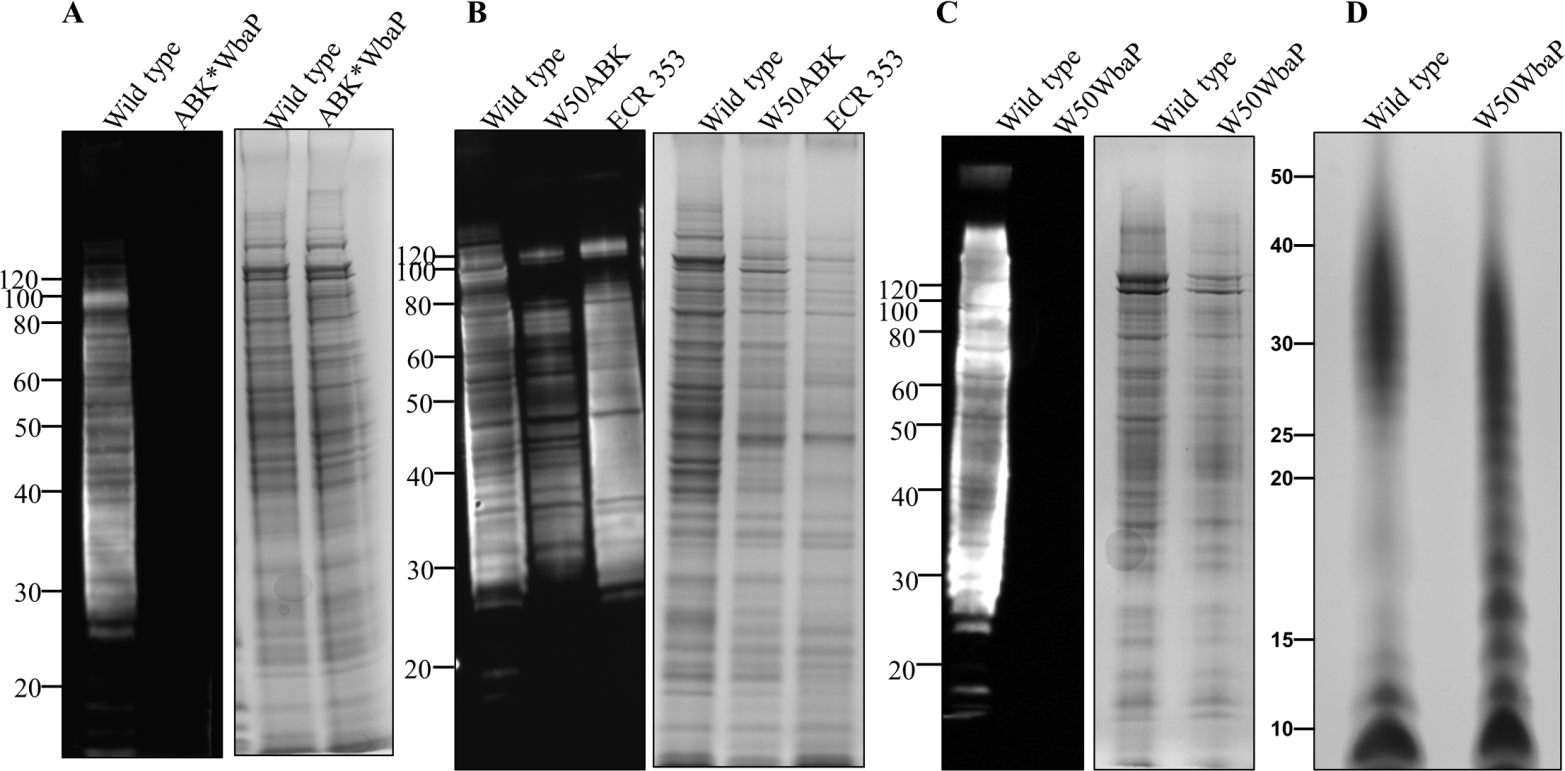
Absence of A-LPS in W50ABK*WbaP and W50WbaP mutants. Immunoblot analysis of whole cell culture lysates with anti-APS (mAb 1B5) antibody. (A) WT and W50ABK*WbaP mutant, (B) WT, W50ABK mutant original stock and ECR353 (another ABK mutant in W50), (C) WT and W50WbaP mutant. Images on the right of each immunoblot (A-C) shows the corresponding commassie stained gel to show a loading control. (D) Silver stained LPS profiles from WT and W50WbaP.

Since one of the proposed roles of A-LPS is to anchor CTD proteins to the cell surface, we questioned whether W50ABK*WbaP mutant cells lacked A-LPS. Cell lysates from *P*. *gingivalis* wild type and W50ABK*WbaP were subjected to immunoblot analysis with anti-APS antibody (mAb 1B5). The W50ABK*WbaP mutant presented no immunoreaction to mAb 1B5, suggesting lack of A-LPS in this strain (Fig 3A). To verify that the lack of A-LPS is not linked to the absence of gingipains in the W50ABK*WbaP mutant we also performed immunoblot analysis on the original stock of W50ABK as well as another W50 triple mutant also lacking all three gingipains (ECR353). These two mutant strains were reactive to mAb 1B5 (Fig 3B) suggesting that the lack of A-LPS in the W50ABK*WbaP mutant is not due to the absence of gingipains but due to a random mutation in *wbaP* that had occurred in this strain. This was further confirmed by analysis of the W50WbaP mutant strain. As expected, the W50WbaP mutant did not exhibit any reaction to mAb 1B5, suggesting that these cells did not synthesise A-LPS (Fig 3C). This confirms earlier results obtained by Shoji et al. for a WbaP (PGN_1896) mutant in *P*. *gingivalis* ATCC 33277 [24]. To explore if WbaP was involved in the synthesis of O-LPS, we purified LPS from wild type and W50WbaP and analysed the samples by SDS-PAGE followed by silver staining (Fig 3D). A ladder of bands was seen in both wild type and W50WbaP mutant cells, suggesting that WbaP is not involved in the production of O-LPS. However, it was observed that the bands in the region of 15–20 kDa were less abundant in the wild type compared to the *wbaP* mutant. Perhaps the absence of A-LPS affects the O-polysaccharide chain length in the *wbaP* mutant.

### Absence of electron dense surface layer (EDSL) in W50WbaP mutant

**Fig 4:**
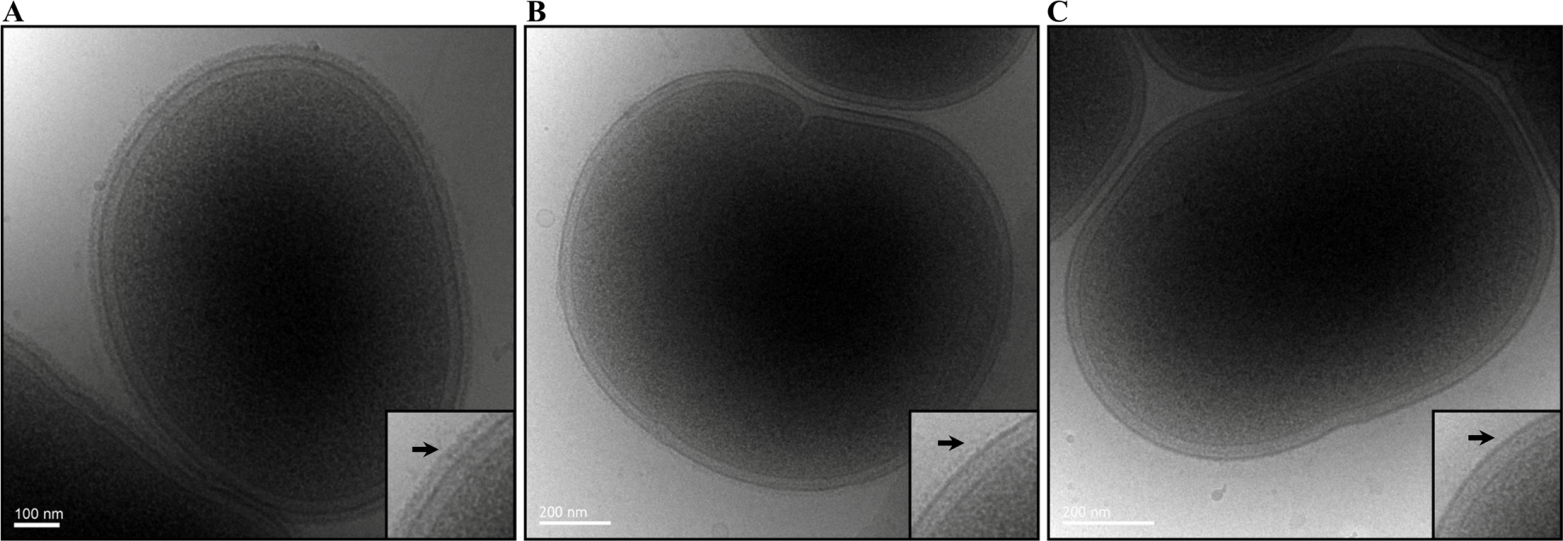
Absence of EDSL on W50WbaP and substantially reduced EDSL on ECR353 (W50ABK). Representative cryo-EM micrographs of (A) Wild type, (B) ECR353 (W50ABK mutant) and (C) W50WbaP. A magnified section is shown on each right bottom corner. Arrows point to the electron-dense surface layer (EDSL) that is absent in W50WbaP and much reduced in ECR353.

To examine if the lack of A-LPS has an effect on the EDSL layer, we performed cryo-electron microscopy on the W50WbaP mutant, ECR353 (*rgpA* ^-^, *rgpB* ^-^ and *kgp* ^-^) and wild type, W50. The micrographs revealed a complete absence of EDSL on the W50WbaP mutant cells (Fig 4C) and a substantially reduced amount of EDSL on ECR353 compared to wild type (Fig 4A and 4B) suggesting that A-LPS is essential for the development of the EDSL while the gingipains are necessary for an EDSL of normal density.

### CTD proteins are released into the clarified culture fluid (CCF) of *wbaP* mutants

**Fig 5:**
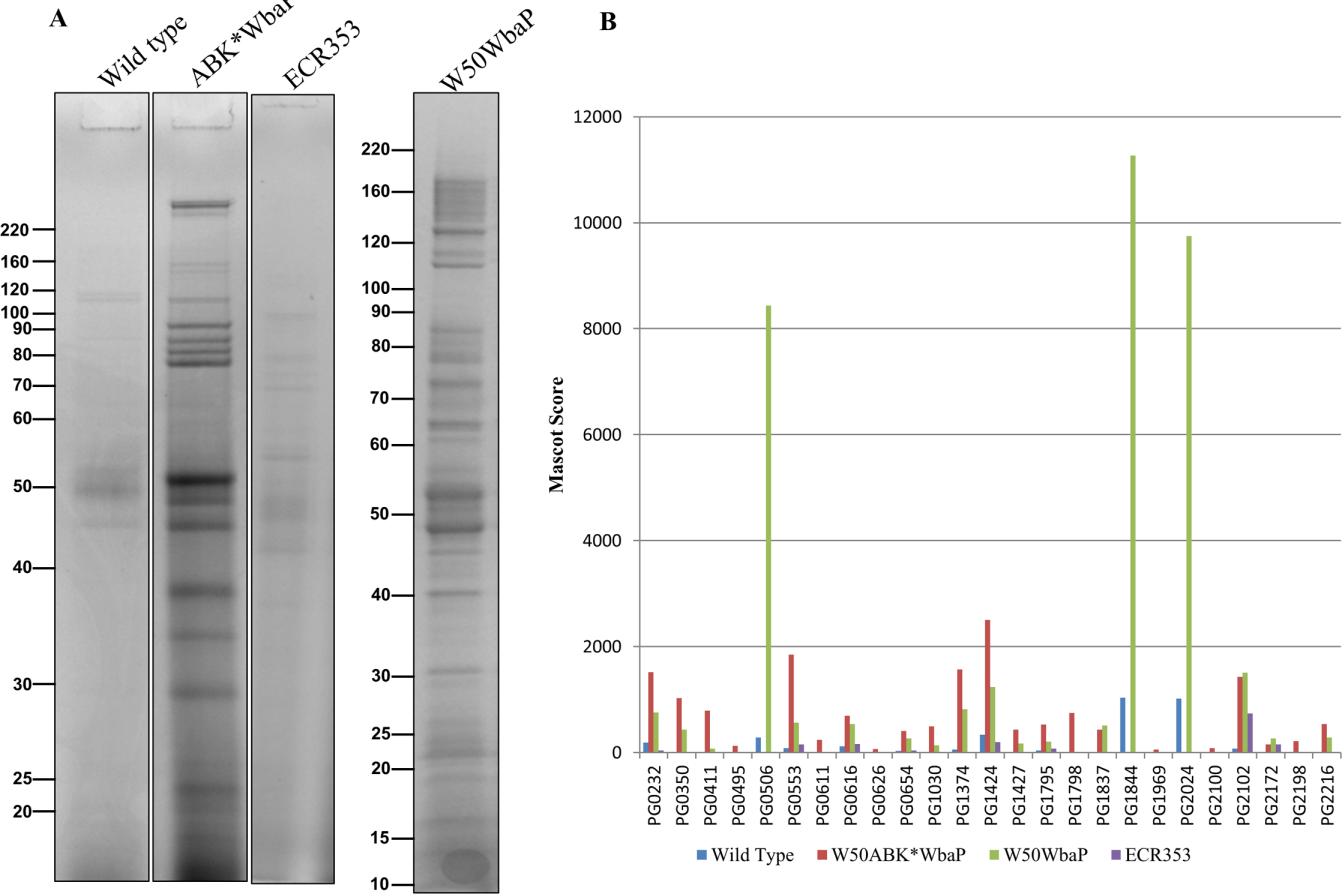
Accumulation of CTD proteins in the CCF of W50ABK*WbaP and W50WbaP mutants. (A) CCF from WT, W50ABK*WbaP, ECR353 and W50WbaP was subjected to SDS-PAGE analysis. The gel was cut into segments and proteins were identified by mass spectrometry. (B) A plot of Mascot scores of identified CTD proteins in CCF of WT, W50ABK*WbaP, ECR353 and W50WbaP. When the same protein was identified from multiple gel segments the Mascot scores were summed (S2 Table).

Since A-LPS was absent in the *wbaP* mutants, and A-LPS is thought to anchor CTD proteins to the surface of cells, we assumed that the CTD proteins had been released into the CCF of these mutants. To confirm this, the CCF of both W50ABK*WbaP and W50WbaP mutants were analysed by SDS-PAGE (Fig 5A). The CCF of the wild type and ECR353 exhibited very few visible protein bands compared with the CCF of the W50ABK*WbaP and W50WbaP mutant strains (Fig 5A). The banding pattern of W50WbaP was different from W50ABK*WbaP due to the presence of gingipains in W50WbaP. The protein bands were identified by mass spectrometry and many of the identified proteins were CTD proteins (Fig 5B and S2 Table). A total of 11, 8, 22 and 18 CTD proteins were identified in the CCF of wild type, ECR353, W50ABK*WbaP and W50WbaP strains, respectively. The CTD proteins identified from the wild type and ECR353 had a considerably lower Mascot score compared with W50ABK*WbaP and W50WbaP consistent with the gel image.

### The C-Terminal domain is cleaved from CTD proteins in W50ABK*WbaP and W50WbaP mutant strains

**Fig 6:**
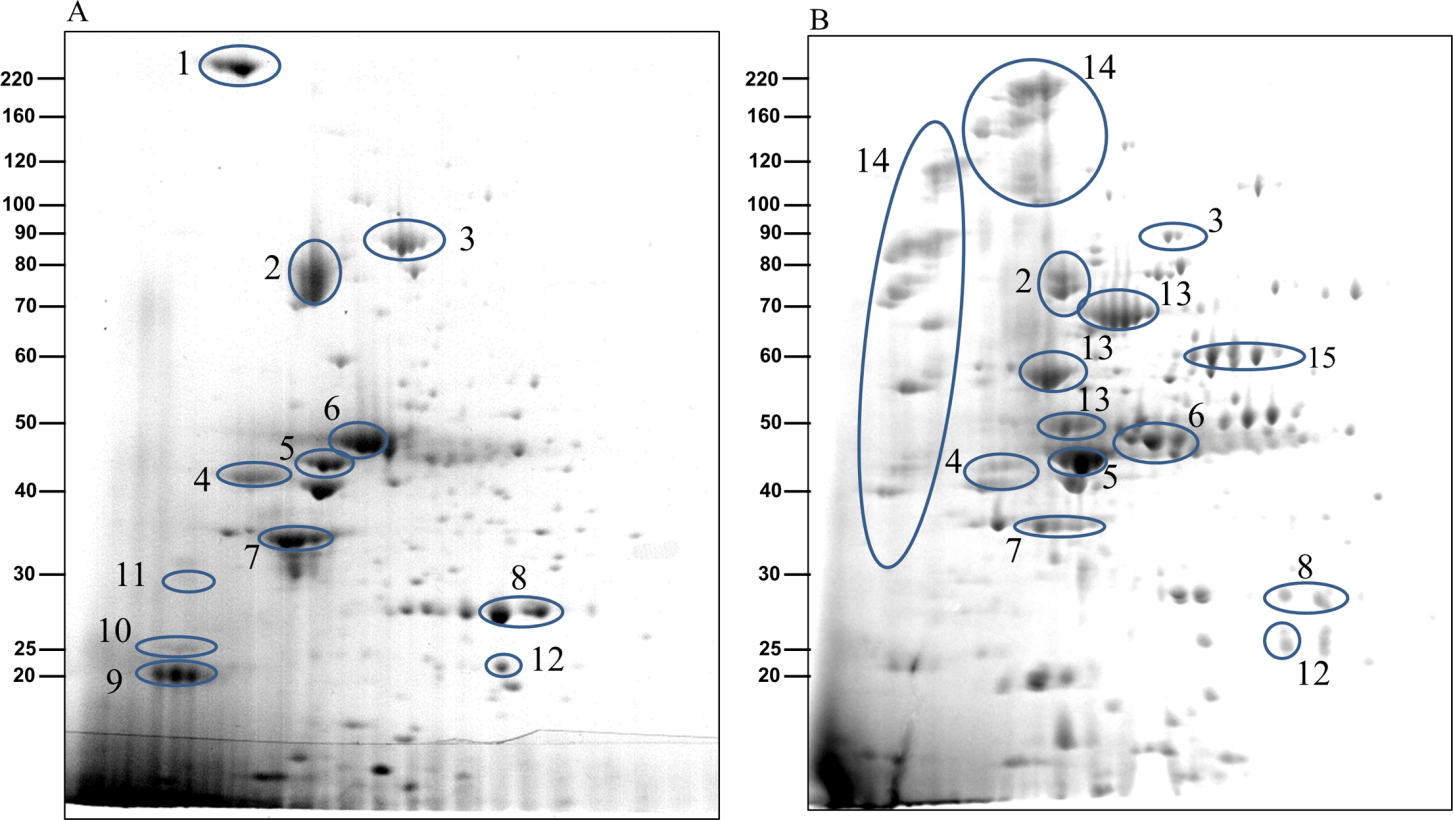
The CTD proteins in W50ABK*WbaP mutant are processed with their C-terminal domains cleaved off. 2-Dimensional gel electrophoresis of CCF from (A)W50ABK*WbaP mutant and (B) W50WbaP mutant. The protein spots were identified by mass spectrometry and selected proteins are listed in Table 1.

To examine if the CTD proteins in the CCF of the W50ABK*WbaP and W50WbaP mutants were proteolytically processed we performed 2-dimensional gel electrophoresis (2-DGE) on the CCF to better determine the molecular weight of the CTD proteins. 2-DGE separates proteins on the basis of isoelectric point and molecular weight therefore a greater partitioning of proteins is achieved with this technique compared with 1-D gel electrophoresis. Fig 6 shows the 2-D gel of the CCF of the W50ABK*WbaP and W50WbaP mutants. The protein spots were identified by mass spectrometry. The observed molecular weight and the calculated molecular weight of the protein after both N- and C-terminal signal cleavages are shown in Table 1. The observed molecular weight matched the predicted molecular weight of the mature CTD proteins with the exception of PG2198 and PG2172 suggesting that the CTD proteins in the CCF of W50ABK*WbaP and W50WbaP mutants are processed with respect to their C-terminal domain being cleaved. Moreover, no peptides from the CTD region were identified in these CTD proteins except for PG2198 and PG2172 where the CTD peptides were identified; this explains the observed higher molecular weight for these two proteins. In the W50WbaP mutant, incompletely processed gingipains were identified. It appears that autolytic processing of gingipains in the culture fluid is slower than the processing that occurs on the cell surface of the wild type. In addition to this, we also analysed the 3–14 kDa region in the 1-D gel of the CCF of W50ABK*WbaP and W50WbaP to look for the cleaved C-terminal signals. The region less than 14 kDa was excised, digested with trypsin and analysed by mass spectrometry. The CTDs of a total of 22 different CTD proteins were identified from the W50ABK*WbaP and W50WbaP mutants and the cleavage sites of 18 of these were identified (Table 2). Gingipains are absent in the W50ABK*WbaP mutant and therefore they were only identified in the W50WbaP mutant. As the CTD cleavage sites for both W50ABK*WbaP and W50WbaP mutants were the same this confirms that the cleavage site observed is not due to processing/cleavage by gingipains. Together, the results suggest that the CTD proteins are fully processed in the mutants, with their C-terminal domain cleaved, but due to the absence of A-LPS they are unable to be properly modified and anchored to the cell surface. To investigate if the cleavage of CTD would occur in any mutant lacking A-LPS, we analysed the low molecular weight region of the CCF of HG66 *P*. *gingivalis* strain, which has been recently shown to contain a defective *wbpB* gene and lacks A-LPS [49]. The absence of A-LPS in our HG66 strain was confirmed by western blotting using mAb 1B5 (S2 Fig). The CTDs of a total of 16 different CTD proteins were identified (Table 2), suggesting normal cleavage of CTD proteins in this strain.

**Table 1:** Identification of CTD proteins in the 2-D gel of W50ABK*WbaP and W50WbaP CCF by MALDI-TOF/TOF MS.

#	Protein Name	MW Obs	MW Calc^a^	PMF Score^b^	MS/MS Score^c^	Seq Cov (%)
1	Hag A	242	223	80/-	-/-	9/-
2	^d^Pro-CPG70	76	79.8	109/201	71/62	20/30
3	^d^Pro-PG0553	86	90.7	187/162	-/74	37/31
4	PG0350	43	42.1	68/36	-/166	21/27
	PG0495	43	42.2	-/-	75/-	-/-
	PG2216	43	42.8	59/71	164 /92	23/18
5	P59	45	49.1	74/134	305/72	25/41
6	PAD	47	49.7	139/133	94/73	41/41
7	PG0654	34	33.7	69/44	334/130	23/15
	PG1374	34	35.7	-/38	55/162	-/17
8	HBP35	28	28.0	207/90	-/89	55/42
9	P27	20	19.7	41/-	75/-	23/-
10	PG1969	25	23.9	83/-	-/-	42/-
11	PG2198	30 inc CTD	21.0	85/-	-/-	27/-
12	PG2172	22 inc CTD	14.4	-/133	85/133	-/45
13	^e^PG0506	50–73	47.7–70.5	-/177	-/88	-/28
14	^e^PG2024	40–190	32–171.2	-/83	-/116	-/9
	^e^PG1844	40–190	53.2–177.6	-/127	-/110	-/18
15	Kgp_Cat_	57	53.2	-/91	-/110	-/6

^a^ Molecular weight calculated from predicted mature N-terminus to identified or predicted mature C-terminus [12, 19].

^b^ PMF, peptide mass fingerprinting. Mascot scores above 46 were significant according to Mascot (p<0.05).

^c^ For MS/MS, Mascot scores above 17 were significant according to Mascot (p<0.05).

^d^ Pro-forms of CPG70 and PG0553 were present in the CCF due to lack of active gingipains to process the prodomains.

^e^Incompletely processed gingipains. Data for only highest scoring spot is included.

**Table 2:** Identification of cleaved CTDs and their putative cleavage sites.

Protein Name	Accession	Identified semitryptic peptide and cleavage site^a^	Mascot Score^*b*^ WbaP/W50ABK*WbaP/HG66	CTD peptides^c^ WbaP/W50ABK*WbaP/HG66
RgpA	PG2024	VDYIPD/GVADVTAQKPYTLTVVGK	56/-/48	1/-/1
RgpB	PG0506	VKVEGT/SIADVANDKPYTVAVSGK	55/-/78	3/-/3
HBP35	PG0616	DKAEPT/ATEQIVATPSVK	-/59/35	2/5/4
CPG70	PG0232	EVILDD/SVEDIVAQTGIVIRPQNGTK	-/21/-	3/4/2
P59 (TapA)	PG2102	QWTHAN/GVEDIVMQEGSMK	73/90/-	4/5/1
Kgp	PG1844	ATLNIT/SLADVTAQKPYTLTVVGK ^e^	84/68^d^/-	1/1^d^/1
	PG0411	LNYTIT/SLDNIQSDTSLK	-/61/63	-/2/1
	PG0553	GSGISN/GVAQIENNNAVVAYPSVVTDR	-/50/-	-/2/1
	PG0626	RILNYQ/SLQEVEQEGIR	-/53/-	-/4/-
	PG0654	TDMQGN/ALTDVAVNESIK	19/107/86	3/6/4
	PG1374	YEGSPT/SNLAVDAPTVR	63/73/73	3/5/2
	PG1427	KIVNGT/AVEAIESSEEIR	-/53/-	-/1/-
P27	PG1795	HLKKGE/GVEAVLTNDK	-/86/-	-/4/-
	PG2172	YKGGGT/DLTNIGLGR	38/60/-	2/5/3
	PG2198	VTVTNS/SLSNVDGQAPYTLR	60/71/72	3/2/2
	PG2216	NKPVIT/SLAAPMSHEIR	49/55/55	3/2/2
	PG1030	KESFIT/SFISPTVVQGVDVYTLAGK	36/76/104	1/5/3
PAD	PG1424	not identified	-/-/-	1/4/3
	PG1548	not identified	-/-/-	2/2/-
	PG1798	not identified	-/-/-	-/2/1
	PG2100	not identified	-/-/-	-/4/-

^a^ The semi-tryptic peptides identified by MS/MS are shown underlined, together with the six amino acid residues immediately N-terminal to the proposed cleavage sites (/).

^b^ The Mascot score shown is for the underlined semi-tryptic peptide (shown in previous column). Scores above 35 were significant according to Mascot (p<0.05), however due to the consistent pattern of results, scores above 20 were deemed significant.

^c^ The total number of CTD peptides identified.

^d^ The peptide identified was from HagA since Kgp was not present in this mutant.

^e^ This Kgp sequence cannot be distinguished from an identical sequence found in the expected cleavage site sequence of HagA (PG1837).

### CTD proteins are modified with the peptides from the growth medium in W50ABK*WbaP

**Fig 7:**
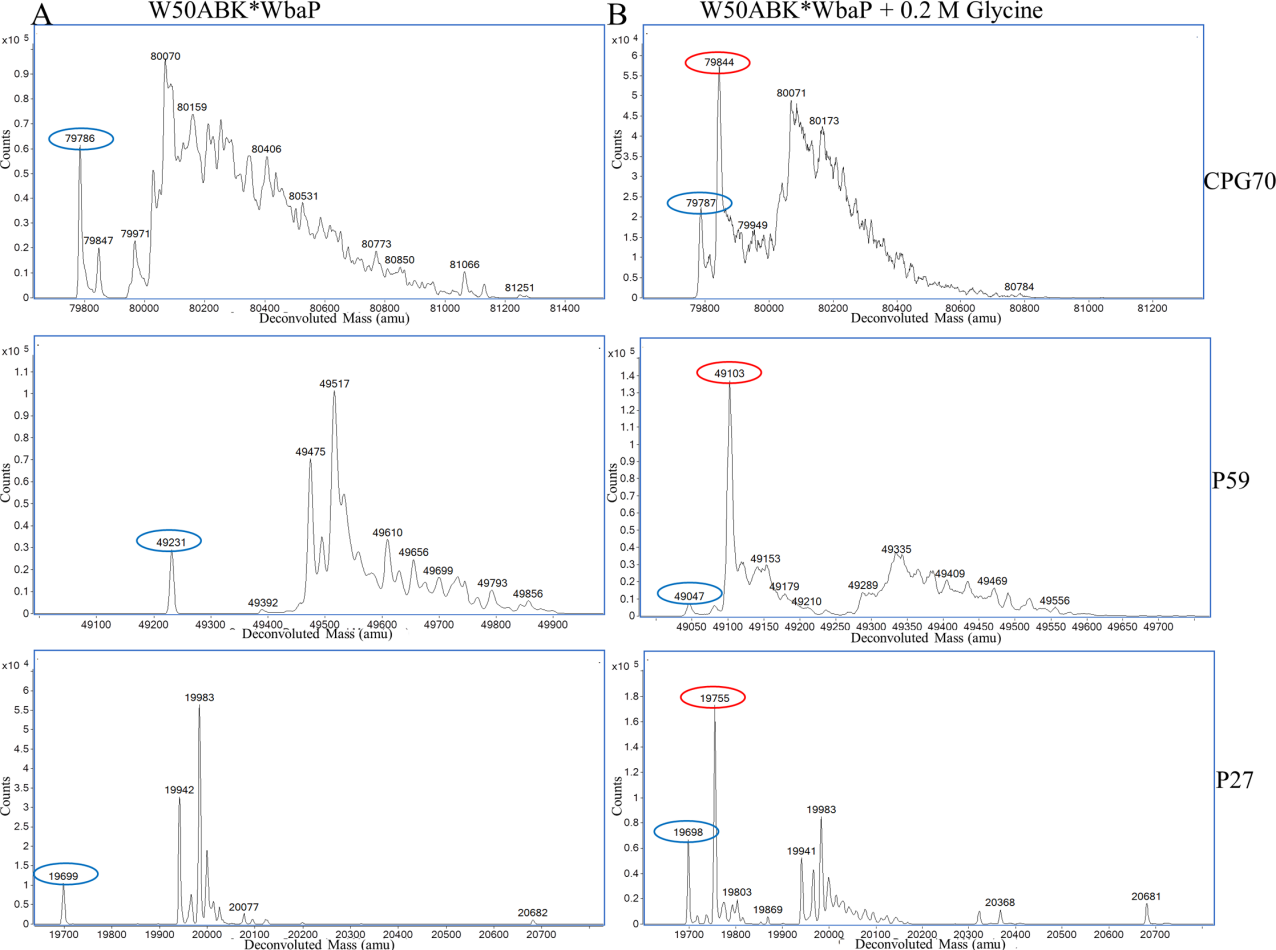
MS analysis of intact CTD proteins shows heterogenous modification of these proteins in W50ABK*WbaP. (A) CPG70, P59 and P27 were purified from the CF of W50ABK*WbaP and analysed by ESI-TOF for accurate molecular mass determination. Circled in blue are the expected molecular mass for each protein, except for P59. (B) W50ABK*WbaP was grown overnight in the presence of 0.2 M glycine. CPG70, P59 and P27 were purified from CF and analysed on ESI-TOF. Circled in blue are the expected molecular mass and circled in red are the expected molecular mass plus glycine (57 Da).

Although the CTD proteins in the CCF were not observed to be extensively modified we could not rule out that a residual modification was present. Furthermore, although the C-termini of the mature CTD proteins are inferred by the CTD cleavage sites, they have not been verified directly. The MS data obtained above from in-gel digests were inconclusive in regard to the position and modification status of the C-termini, and therefore selected CTD proteins, namely pro-CPG70 (PG0232), P59 (PG2102) and P27 (PG1795) were purified in-solution to allow a more comprehensive MS analysis to be undertaken. All three proteins were purified from the W50ABK*WbaP culture fluid using anion exchange and pro-CPG70 was purified further by gel filtration. The purity of the proteins was assessed by SDS-PAGE (S3 Fig). Purified pro-CPG70, P59 and P27 were first analyzed by ESI-TOF MS to determine their accurate molecular mass (Fig 7A). A compound of 79,785 Da was observed for pro-CPG70 (expected mass 79,786 Da) and 19,698 Da for P27 (expected mass 19,698 Da) suggesting the presence of unmodified pro-CPG70 and P27. However, a peak corresponding to unmodified P59 (expected mass 49,046) was not observed (Fig 7A), but rather the smallest mass detected was 49,231 Da indicating the presence of a modification or a sequence difference in this protein. The expected molecular masses were calculated from mature N-terminus to the mature C-terminus as predicted from the CTD cleavage sites (Table 2). However, in addition to these peaks, numerous other compounds up to 1,500 Da larger than the expected molecular mass were observed (Fig 7A). A common feature for all three proteins was a difference of approximately 285 Da between the highest peak observed and the peak of smallest mass (Fig 7A), indicating the same major modification occurring in all three proteins. Furthermore, the modified compounds together were much more abundant than the unmodified form for all three proteins (Fig 7A).

**Fig 8:**
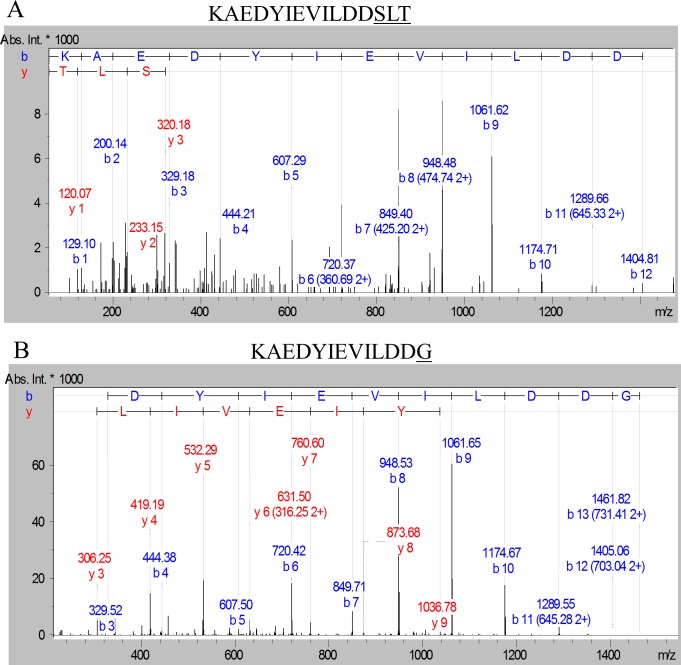
Modification of the C-terminal peptide of Pro-CPG70 with peptides or glycine. Purified Pro-CPG70 from W50ABK*WbaP was subjected to in-solution digest with trypsin and the tryptic fragments were analysed with LC-MS/MS (Orbitrap). (A) An example MS/MS spectrum of C-terminal peptide of mature Pro-CPG70 showing modification with ‘SLT’ at the C-terminus in the absence of glycine, see **Table 3** for more modifications. (B) An example MS/MS spectrum of C-terminal peptide of mature Pro-CPG70 showing modification with ‘G’ at the C-terminus in the presence of 0.2 M glycine in the growth medium.

To explore what the higher molecular weight compounds were, in-solution tryptic digestion was performed on the purified protein samples and analyzed by LC-MS/MS using the Orbitrap mass spectrometer which has higher mass accuracy than the ion trap instrument used previously for in-gel digests. The peptides matching the expected C-terminal peptides of mature Pro-CPG70 and P59 were identified. Manual searching of the MS/MS data for modified C-terminal peptides led to the identification of more than 13 different peptides from Pro-CPG70 (S4 Fig and Table 3). The accurate mass of each putative modification was calculated and used to search the Metlin database for matching metabolites. In each case, the modification mass was found to accurately match to a peptide, amino acid, or in one case ammonia (S4 Fig and Table 3). No matches to other compound classes such as sugars were observed. The most intense peak was of the unmodified C-terminal peptide, KAEDYIEVILDD, followed by the +GVQ (+284 Da) peak (S4 Fig and Table 3), consistent with the major +285 Da peak observed in the ESI-TOF data. Fig 8A shows an example MS/MS spectrum of one of the peptides detected that matched the expected C-terminal tryptic peptide of Pro-CPG70 (KAEDYIEVILDD) but had an extra three amino acid residues present (-‘SLT’-) which is evident in the spectrum. It appears that the CTD proteins are modified with peptides after cleavage of their CTD. The peptide modification at the C-terminus was also evident in the W50WbaP mutant. 2-D gel spots corresponding to P59 and Kgp were analysed using Orbitrap MS. Manual analysis of the spectra revealed multiple peptide modifications at the C-terminus of P59 and Kgp (S5 Fig). Together, these data suggest a sortase/transpeptidase type of reaction is occurring. Known sortases in Gram-positive bacteria recognize an LPXTG motif, present in proteins destined for secretion. The sortase cleaves the threonine-glycine peptide bond forming an intermediate thio-ester bond with the sortase active site cysteine that is susceptible to nucleophilic attack by either water or preferably by a free amine [50]. In the current study, in the absence of A-LPS this putative thio-ester bond reacted with peptides present in the growth medium (modified) or was hydrolysed by water (unmodified).

**Table 3:** Modified C-terminal peptides identified from CPG70 tryptic digests using the Orbitrap MS with and without the inclusion of 0.2 M glycine in the growth medium. CPG70 was purified from W50ABK*WbaP.

Mass Obs (Da)	^a^Delta Mass (Da)	^b^Best Match	^b^Error (ppm)	^c^Relative Intensity	^c^Relative Intensity (Gly)
1421.6930	0 (ref)	Water	0 (ref)	100	100
1705.8414	284.1484	G(VQ)	0	90	58
1664.8160	243.1230	G(VS)	4	78	64
1707.8204	286.1274	GTQ	0	30	11
1816.9090	395.2160	GLPQ	1	28	18
1688.8514	267.1584	GLP	0	27	17
1799.8934	378.2005	LQH	2	23	17
1708.8414	287.1484	S(VT)	1	20	13
1923.9314	502.2384	(GMWK)	4	20	-
1846.9210	425.2280	SLPQ	1	18	12
1549.7858	128.0929	K	13	17	-
1722.8574	301.1644	SLT	2	17	9
1577.7934	156.1004	R	3	15	-
1420.7088	-0.9842	Ammonia	2	13	12
1478.7134	57.0210	G	4	-	219

^a^ The delta mass is the observed mass minus the observed mass of the unmodified (water-cleaved) peptide (KAEDYIEVILDD, 1421.6930 Da). With the exception of the glycine-modified peptide, all observed masses are taken from the untreated sample.

^b^ The best match is the metabolite most closely matching the accurate delta mass value using the Metlin database (see Methods). In every case, the best match was a peptide or amino acid. For peptides, the order of amino acids was inferred from the MS/MS data, with the order of bracketed amino acids being uncertain. The mass error (in ppm) associated with this search is provided in the next column.

^c^ The intensity of the strongest MS peak, relative to the unmodified reference peak for the untreated sample. The values for the glycine-treated sample are provided in the next column.

### Addition of glycine in the growth media of W50ABK*WbaP and W50WbaP causes CTD proteins to be modified with glycine

**Fig 9:**
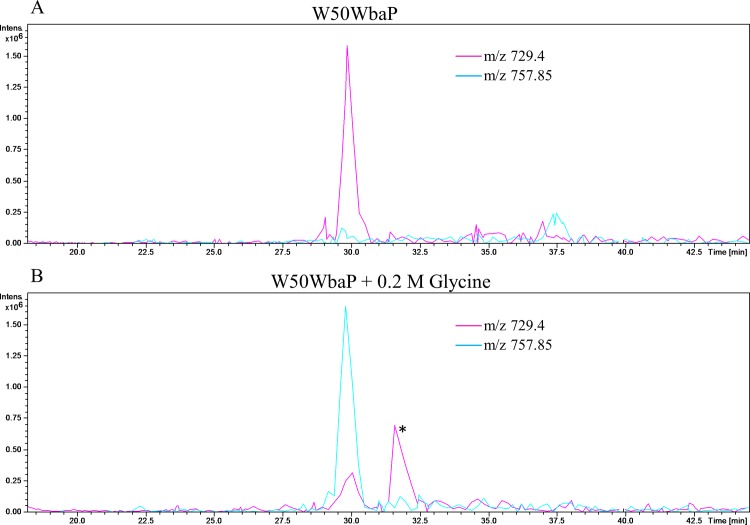
The C-terminal peptide of P59 is modified with glycine from the growth medium. W50WbaP was grown overnight in the (A) absence or (B) presence of 0.2 M glycine. The proteins in the CCF were subjected to SDS-PAGE separation and the band corresponding to P59 was excised and in-gel digestion was performed. Tryptic fragments were analysed by LC-MS/MS (Ion trap). Extracted ion chromatograms were plotted for m/z 729.4 (IVWSDTQWTHAN) and m/z 757.85 (IVWSDTQWTHANG). These peaks were identified as C-terminal peptide of P59 using Mascot search. * Peak corresponding to another protein.

If the modification of CTD proteins is via free amines present in the growth medium then addition of an amine such as glycine to the medium should lead to the glycine modification of the C-terminal peptide of CTD proteins. To test this prediction, W50ABK*WbaP was grown in the presence of 0.2 M glycine and Pro-CPG70, P59 and P27 were purified from the culture fluid as described above and analysed using ESI-TOF (Fig 7B). Peaks corresponding to glycine modification (+57 Da) were observed for all 3 proteins (Pro-CPG70- 79,844 Da, P59- 49,103 Da and P27- 19,755 Da) and were the highest peaks indicating that the glycine modified proteins were the most abundant species present (Fig 7B). Notably, other peaks that were detected in the untreated sample, such as 19,983 Da and 19,942 Da for P27, were also detected in the glycine treated P27 sample (Fig 7B). A similar observation was also made for Pro-CPG70 and P59. Of note, the expected theoretical mass of P59 (49,046 Da) was observed in the glycine treated sample compared with the untreated sample where a mass of 49,231 Da was detected (Fig 7B). Conceivably, the addition of glycine to the medium may have inhibited production of the other modification present in P59. To ensure that the glycine modification was at the C-terminus, in-solution digestion was performed on Pro-CPG70 and the tryptic fragments were analysed using LC-MS/MS (Orbitrap). CPG70 was selected because the next residue after the predicted C-terminal cleavage is not glycine (VILDD/S) whereas P59 has glycine as the next residue (THAN/G) (See Table 2). This distinction was crucial to ensure that the result we observed was due to modification rather than miscleavage by the peptidase. The MS/MS spectrum shows the modification with glycine at the C-terminus of Pro-CPG70 (Fig 8B). Of note, this glycine modified peptide peak was absent in the untreated sample and was twice the intensity of the unmodified peptide peak in the treated sample (Table 3). To verify that peptide modification also occurs in W50WbaP, W50WbaP was grown in the presence and absence of 0.2 M glycine and the proteins in the CCF from both conditions were separated on SDS-PAGE. Since the CPG70 C-terminal peptide could not be readily identified from in-gel digests, the band corresponding to P59 was excised and subjected to in-gel tryptic digestion and the tryptic fragments were analysed by mass spectrometry. In the absence of glycine, unmodified P59 C-terminal peptide, IVWSDTQWTHAN was identified with a Mascot score of 80, while in the presence of 0.2 M glycine, both IVWSDTQWTHAN (score = 69) and IVWSDTQWTHAN**G** (score = 39) were identified. When the ion chromatograms for these two doubly charged peptides were extracted, it could be seen that the glycine modified peptide peak of m/z 757.85 was approximately five times more intense than the unmodified peptide peak of m/z 729.4 (Fig 9B) in the glycine treated sample, whereas the m/z 757.85 peak in the untreated control was very small (Fig 9A). These data confirm the modification of mature CTD proteins with free amines from the growth medium in mutants lacking a functional WbaP protein.

### Addition of glycine into the culture medium of wild type *P*. *gingivalis* W50

To discover if excess glycine in the growth medium can compete with A-LPS dependent modification and therefore release surface proteins in the wild type, *P*. *gingivalis* W50 was grown in the absence or presence of 0.2 M glycine. The proteins in the CCF were subjected to SDS-PAGE and mass spectrometry analysis (S3 Table). In the presence of glycine, a total of 14 CTD proteins were identified compared to only 6 CTD proteins in the absence of glycine, However, the most abundant proteins identified in the presence of glycine were cytoplasmic/periplasmic proteins, suggesting increased cell lysis and making it difficult to determine whether glycine was able to release CTD proteins from the cell surface. Overall, the abundance of CTD proteins in the glycine treated CCF was very low suggesting that glycine may only compete weakly with A-LPS to modify the CTD proteins.

### In the wild type, CTD proteins are modified at the C-terminus with a potentially novel linker via a peptide bond

**Fig 10:**
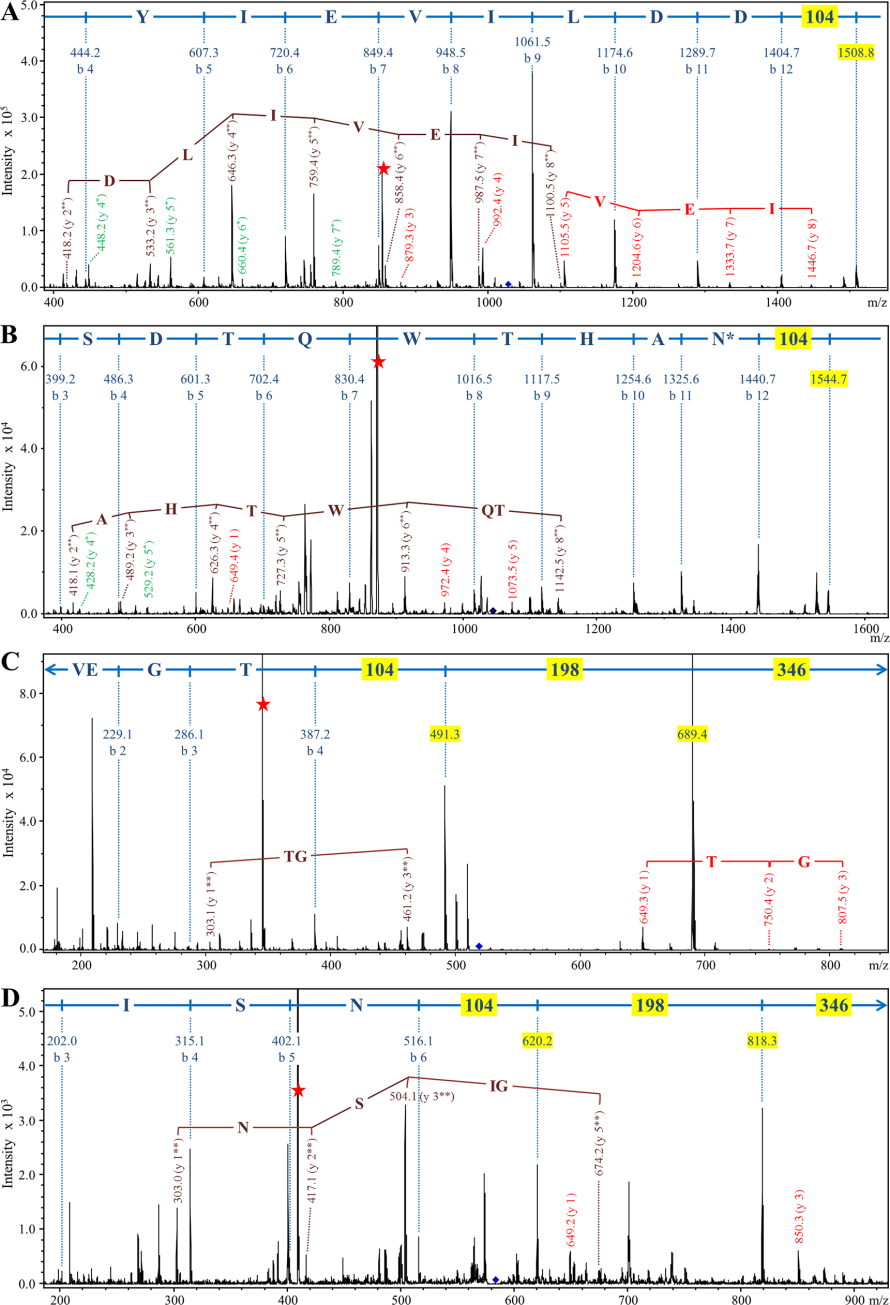
Modification of CTD proteins isolated from wild type W50 with the 648 Da linker. Modified CTD proteins were semi-purified, deglycosylated with TFMS, digested in-gel with trypsin and analysed by LC-MS/MS in positive ion mode using the ion trap MS. Each panel shows the MS/MS spectrum obtained for a C-terminal trytic peptide modified with the 648 Da linker. (A) CPG70, peptide sequence = KAEDYIEVILDD. (B) P59, peptide sequence = IVWSDTQWTHAN. (C) RgpB, peptide sequence = VEGT. (D) PG0553, peptide sequence = GSGISN. The b-ions and their corresponding sequence assignments are shown in blue with masses (104, 198 and 346) corresponding to components of the linker highlighted yellow. The y-ions are labeled such that the ion corresponding to the modification alone is y-1. Y-ions beginning with the intact (648 Da) modification are in red, and are labeled y 1, y 2 etc. Y-ions beginning with the 302 Da component of the linker are in brown and are labeled y 1**, y 2** etc. Y-ions beginning with the 105 Da component of the linker are in green and are labeled y 1*, y 2* etc. The intense peaks labeled with a red star are doubly charged ions that have lost the 346 Da component of the linker. N* denotes partial deamidation.

To identify the natural modification at the C-terminus of the wild type and to determine whether it is linked to the CTD proteins via a peptide bond, we partially purified modified CTD proteins from outer membrane vesicles by anion exchange (S6 Fig). After SDS-PAGE and MS analyses of the chromatographic fractions, fractions were combined to make five pools labeled A-E (S7 Fig). The target CTD proteins in each pool were A) CPG70 & PAD, B) RgpB, C) P59, D) RgpA_A4_ and P27, and E) Kgp_A5_. As the modification is very large and heterogenous, each pool was subjected to deglycosylation with TFMS to make it more amenable to MS analysis. The TFMS treated samples were separated on SDS-PAGE gels, subjected to in gel tryptic digestion and analysed by LC-MS/MS to identify the C-terminal peptides that were presumed to be modified. For CPG70, the database search of the resultant data allowing for ‘semi-tryptic’ cleavages gave a Mascot score of 1469 and a sequence coverage of 59% from mature N-terminus (YEWN…) to the most C-terminal peptide identified (YDVQ…SNVK). This peptide is adjacent to the expected C-terminal peptide according to the identified cleavage site (Table 2) suggesting a residual modification on the C-terminal peptide. An error tolerant Mascot search of the data also did not identify the expected C-terminal peptide suggesting that the modification was not present in the unimod database. Therefore, the MS/MS data for CPG70 was searched manually leading to the identification of the C-terminal peptide (KAEDYIEVILDD) with extensive matches to the N-terminal fragment ions (b-ions) up to and including the C-terminal residue (Fig 10A). The deduced MW of the modification was +630.3 Da. The same approach was used to identify the C-terminal peptide of P59 which exhibited an uninterrupted b-ion series from b-3 to b-12 which includes the expected C-terminal Asn residue (Fig 10B). Again, the deduced MW of the modification was +630.3 Da suggesting the same residual modification was present on both CTD proteins.

Accordingly, the modified C-terminal peptides of the other target CTD proteins along with three additional CTD proteins were identified by searching for the same modifications (Figs 10 and S8). Analyses of the MS/MS spectra of these peptides revealed a clear pattern. In each case the b-ion series appeared to continue with +104, and +198 peaks leaving +346 Da to reach the mass of the parent ion (Fig 10C and 10D). In addition, each doubly charged parent exhibited a neutral loss of m/z 173 (346 Da) indicated by a red star in Fig 10. In every case, b-ions consistent with modification of the C-terminal amino acid residue alone were observed. Other ions were also observed for many of the spectra and included ions at m/z, 199 (198+H^+^), 303 (198+104+H^+^), 649 (346+198+104+H^+^) and y-ion series starting with the modification at m/z 649 plus the mass of peptide fragments starting with the C-terminal amino acid residue (Fig 10B, 10C, and 10D). Since the 346 Da component was very labile, the y-ion series starting with m/z 105 (annotated in green) and m/z 303 (annotated in brown) were also observed in some cases confirming that the modification is linked to the C-terminal amino acid residue via the 104 Da entity. The accurate masses obtained for this 104 Da entity using Orbitrap LC-MS/MS were 104.059 and 104.060 (S8 Fig), which within the mass accuracy of the experiment (± 0.002 Da) and allowing for the possible presence of C, H, N, O, P, S atoms, could only be matched to the molecular formula C_3_H_8_N_2_O_2_. The presence of nitrogen is consistent with the expected peptide linkage.

**Fig 11:**
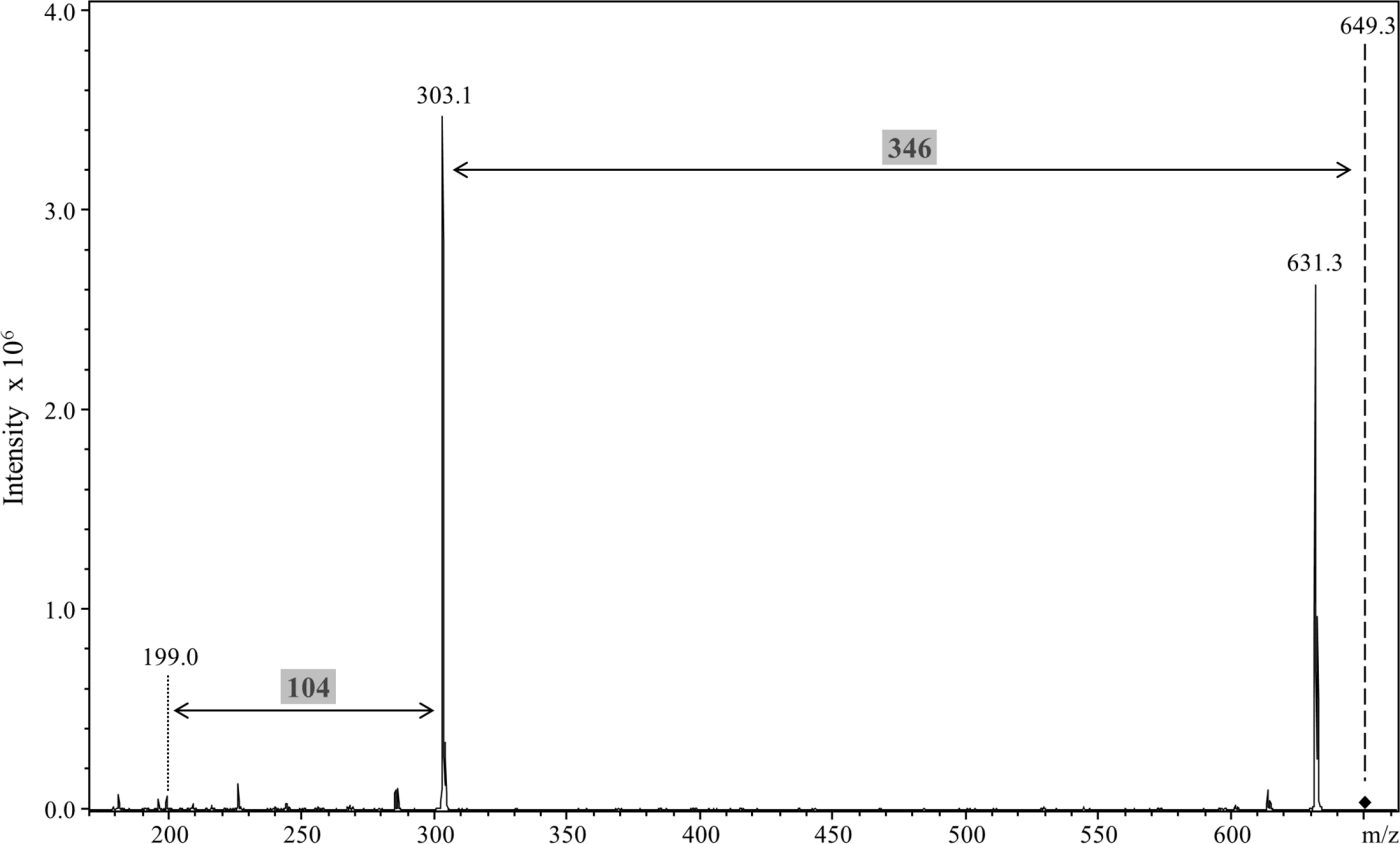
Release of the 648 Da linker by proteinase K cleavage. Modified CTD proteins were semi-purified, deglycosylated with TFMS, digested with proteinase K and analysed by LC-MS/MS in positive ion mode using the ion trap MS. A singly charged peak of m/z 649.3 was obtained and MS/MS analysis showed a major loss of 346 Da to produce the m/z 303.1 ion. An additional fragment ion at m/z 199 corresponded to a further loss of 104 Da.

To confirm that the modification is conjugated to the CTD proteins via a peptide bond, the deglycosylated CTD proteins were digested with the non-specific protease, proteinase K. LC-MS analyses of the proteinase K digested samples produced an intense signal at m/z 649, which is consistent with the mass of the modification released from the various peptides. MS/MS of the m/z 649 ion produced the characteristic neutral loss of 346 Da confirming its identity as the previously detected modification (Fig 11). The same result was obtained for CTD proteins from each pool confirming the same modification was present in all the CTD proteins purified. Release of the modification by proteinase K digestion suggests that it is linked via a peptide bond to the CTD proteins.

## Discussion

WbaP is one of the proteins required for APS synthesis and its proposed role is to link a sugar residue onto undecaprenyl monophosphate (UndP) [24]. Shoji et al. [24] using immunoblotting with specific antibodies demonstrated that a *wbaP* isogenic mutant lacked A-LPS but still produced O-LPS. In addition, they showed that the *wbaP* mutant lacked the modified forms of the CTD proteins, RgpB and HBP35 consistent with A-LPS being the modification that anchors CTD proteins to the cell surface. However, the exact mechanism of A-LPS conjugation to the CTD proteins is unknown. In this study, we further characterised WbaP mutants and wild type strains and identified a mechanism for A-LPS conjugation to the CTD proteins.

The electron dense surface layer (EDSL) of *P*. *gingivalis* is located peripheral to the outer membrane [21]. Mutants such as *PorT*, *LptO* and *PG0026* that are defective in the secretion of CTD proteins have no EDSL [19, 21]. Similarly mutants that lack A-LPS, such as PorR also display no EDSL [15]. However, to date the exact constituents of this EDSL are unclear. In this study we show a complete lack of EDSL in the W50WbaP mutant and a much reduced EDSL in the gingipain triple mutant ECR353. Previously, Chen *et al*. [21] showed a complete absence of EDSL on *P*. *gingivalis* that lacked all three gingipains. However, we have now found that the mutant that was analysed by Chen *et al*. had developed a spontaneous mutation in the *wbaP* gene. Therefore, the complete lack of EDSL could not be attributed to the absence of the gingipains alone but to the lack of A-LPS caused by the *wbaP* mutation. Since ECR353, a true triple gingipain mutant, had a much reduced EDSL, it can be concluded that cell attached gingipains do form a significant part of the EDSL, however, it appears that other CTD proteins and/or A-LPS may also be involved in the formation of this layer. The complete absence of EDSL in mutants defective in A-LPS may be explained by no attachment of CTD proteins to the surface of the outer membrane in these mutants. Alternatively, A-LPS itself, being highly phosphorylated and presumably associated with metal ions may also contribute directly to the EDSL.

The CTD proteins were accumulated in the clarified culture fluid (CCF) of both *wbaP* mutants with their CTDs cleaved. At this point of the study, it appeared that CTD cleavage occurred independently of modification, since normal cleavage appeared to be occurring, yet A-LPS modification was absent. CTD cleavage thus appeared to be independent of the presence of A-LPS, a conclusion also reached by others [51]. This conclusion was however difficult to reconcile with the co-accumulation of CTD proteins and A-LPS in the periplasm of secretion mutants [19, 21], the widespread occurrence of extensively modified T9SS substrates [12], and the combined secretion and modification of an artificial substrate–GFP [52]. These findings led us to propose that the T9SS was a coordinated or combined secretion and attachment system [12]. While, it has been known for some time that CTD proteins are secreted into the culture fluid of mutants lacking A-LPS, it was uncertain whether the proteins were transiting through the T9SS normally. The finding of normal CTD processing (Table 2) suggested that apart from modification and cell surface attachment, the secretion process was otherwise normal. However, MS analyses of the processed CTD proteins in the CCF of *wbaP* mutants demonstrated that simple CTD hydrolysis had not in fact occurred. While some CTD protein molecules were observed to have been simply cleaved (hydrolysed) at the correct amino acid residue, most molecules were found to be modified with peptides from the growth medium (S4 Fig and Table 3). In each case, the most C-terminal residue observed for the natural sequence that was also common to all modified peptides identified was Asp738 for CPG70 and Asn455 for P59 (unmodified C-terminal peptides ending in …VILDD and …WTHAN, respectively).

Therefore, CTD cleavage occurs at the site we have determined, and in the *wbaP* mutants peptides from the growth medium are added to the new C-terminus thereby extending the length of the protein. In addition to peptides, single amino acids including glycine, arginine and lysine were also observed to modify the C-terminus. Glycine has only two reactive groups, namely a primary amine and a carboxylic acid. In many of the peptide modifications, it was a glycine residue that was found to have reacted with the C-terminus, and therefore the observed bond between the peptide and the C-terminus must be via the amine, and therefore most likely a peptide bond. Further evidence for the nucleophilic attack being from an amine comes from the detection of an amidated C-terminal peptide of CPG70 which is the result of nucleophilic attack from an ammonia molecule (Table 3).

The linkage of one protein/peptide to another is referred to as a transpeptidation reaction, and in this context is reminiscent of the sortases of Gram-positive bacteria. Sortase A is a transpeptidase responsible for the attachment of secreted proteins to the cell wall in Gram-positive bacteria [50]. In *Staphylococcus aureus*, proteins targeted to the bacterial surface contain an N-terminal Sec leader sequence and a C-terminal cell wall sorting signal that bears an LPXTG motif [53]. Upon recognition of this signal, sortase A cleaves between threonine and glycine residues [54] to form an acyl-enzyme intermediate [50]. The active site cysteine of sortase A bonds with the carbonyl of the threonine residue of the target protein. This intermediate is then resolved by nucleophilic attack from an amine of the pentaglycine cross-bridge of the peptidoglycan, therefore tethering the protein to the cell wall and regenerating the sulfhydryl group in the enzyme’s active-site [50].

The peptide modifications of CTD proteins observed in the *wbaP* mutants may therefore be generated by a sortase-like protein in *P*. *gingivalis*. The extension of this, is that the natural A-LPS modification observed in the wild type is also likely to be conjugated to the C-terminus of CTD proteins via a peptide bond by the same sortase-like protein. Analysis of the CTD proteins in the wild type confirmed this proposal. In total, ten different CTD proteins were shown to be modified with a 648 Da linker, including the three gingipains, RgpA, RgpB and Kgp. The data conclusively showed that the modification was linked to the C-terminal residue, either Thr (RgpB, Kgp, PG1030, HBP35, PAD), Asp (RgpA, CPG70), Glu (P27) or Asn (P59, PG0553) consistent with modification at the carboxyl terminus rather than the side chain and in agreement with the determined CTD cleavage sites (Table 2). The component of the linker bonded to the CTD protein has an accurate mass of 104.059 Da and a deduced molecular formula of C_3_H_8_N_2_O_2_. The presence of nitrogen is consistent with forming a peptide (amide) bond to the protein’s carboxyl terminus. A peptide linkage was confirmed by its successful cleavage with proteinase K to release the intact 648 Da linker (Fig 11). Comparison of the MS data with the published structures of O-LPS and A-LPS [55] does not provide any matches as there are no components that have C_3_H_8_N_2_O_2_. The remaining components of 198 and 346 Da also cannot be readily matched to known LPS components suggesting that the linker is an additional component of A-LPS whose structure is yet to be determined. Recently, Shoji et al., identified the putative presence of a Wbp pathway in *P*. *gingivalis* that included the enzymes, WbpA, WbpB, WbpD and WbpE that together synthesise a di-acetylated glucuronic acid [49]. Deletion of any of the genes for this pathway leads to the loss of A-LPS even though the published structure of A-LPS contains neither di-acetylated sugars, nor uronic acid derivatives [55]. The authors therefore suggested that the full structure of A-LPS may not yet be determined. We therefore propose that the 648 Da linker is a novel component of A-LPS that allows it to be conjugated to the CTD proteins. Since the linker was isolated after deglycosylation, the attachment point to A-LPS could be a lipid A sugar, a core sugar or an APS sugar. The exact structure of the linker and its attachment to the known portion of A-LPS is under current investigation in our laboratories.

What we observe in the *wbaP* mutants therefore is the result of the sortase-like protein being unable to utilise the correct substrate for modification (A-LPS). In the absence of A-LPS, the sortase-like protein appears to be able to utilise a range of amine-containing molecules that are present. If this is the case, it would be expected that the findings observed in this study for *wbaP* mutants would be replicated in other mutants lacking A-LPS. *P*. *gingivalis* strain HG66 has long been known to secrete soluble (unmodified) CTD proteins into the culture fluid, and in this study we confirmed that the CTDs of these proteins are cleaved at the usual sites (Table 2). The HG66 strain was recently found to lack A-LPS due to a mutation in the *wbpB* gene [49] and therefore its CTD proteins should also be modified with peptides from the growth medium. The crystal structure of RgpB that was purified from the HG66 strain was described to contain Tyr^1^ through to Ser^435^ potentially with additional residues at the C-terminal end that would extend into a large crystal cavity [56]. The presence of Ser^435^ in the crystal appeared to contradict the experimentally determined cleavage site (Thr^434^). Ser^435^ was however not in the final FFRCMK structure, and a check of the available electron density map revealed that the side-chain of Ser^435^ was not resolved, and could not be distinguished from other small amino acids such as Gly. Hence, the crystal structure data does not in fact contradict cleavage at Thr^434^, nor does it contradict the presence of variable peptide additions to the sequence beyond Thr^434^.

In the work of Zhou *et al*. certain mutations introduced close to the cleavage site of RgpB, caused RgpB to be released into the culture fluid in a soluble, unmodified state leading the authors to conclude that the modification process was distinct from CTD cleavage [51]. However in the light of the present study, it appears more likely that these unmodified forms were released by the action of other proteinases besides PG0026, such as the Lys-gingipain, Kgp. This is plausible since there are several Lys residues in the vicinity of the cleavage site of RgpB (Lys^422^, Lys^427^, Lys^430^ and Lys^443^) that may have become exposed to Kgp due to the sizeable mutations employed. Significantly, the authors concluded that their mutations had altered the conformation of the local structure. Since we have shown here that modification may be occurring in the same step as cleavage through the action of a sortase-like protein, modification would not occur (or remain) if Kgp cleaved the recombinant RgpBs prior to transpeptidation or if the proteolysis was N-terminal to the modified Thr^434^ residue.

The following outlines the evidence for CTD cleavage and modification via a sortase-like mechanism in *P*. *gingivalis*. (i) The CTD proteins have a C-terminal signal that is cleaved prior to modification of the mature protein via a peptide bond. The modification enables the attachment to the cell surface in *P*. *gingivalis*. This process is reminiscent of the way in which certain cell surface proteins in Gram-positive bacteria that also exhibit a C-terminal signal, are cleaved and modified via a peptide bond through the action of a sortase, enabling them to be tethered to the cell wall. (ii) In the absence of the natural substrate, A-LPS, CTD proteins are cleaved and modified with peptides/amino acids (in preference to water) via a peptide bond. (iii) The cleavage site of sortase A (T/G) [54] is similar to the cleavage site in CTD proteins (X/(GSA) [12]. In both cases, a small residue is preferred after the cleavage site. (iv) The amino acid preference shown in peptides from the growth medium (GS)(VLI) (Table 3) reflects the cleavage site sequence (GSA)(VLI) (Table 2) [12], suggesting the same binding pocket is used. Similarly, the Gram-positive sortase substrate linked via its pentaglycine crossbridge to the cell wall effectively substitutes a crossbridge Gly residue for the Gly residue in the sorting signal [57]. This is consistent with the action of a single enzyme (a sortase) used for cleavage and modification rather than two separate enzymes. Further to this, sortases are used *in vitro* to catalyse the transpeptidation of a wide variety of substrates such as fluorophores, biotin, proteins and lipids via an oligoglycine peptide linker [58]. (v) In addition to transpeptidation products we also observed unmodified C-terminal peptides reflective of proteolysis. Sortases are also known to be promiscuous with water, the intermediate acyl-enzyme can be resolved with hydrolysis [59]. Together, these observations point to the cleavage/modification of CTD proteins by a sortase-like protein. In *P*. *gingivalis*, we have shown PG0026 to be the C-terminal peptidase [19]. PG0026 is a cysteine proteinase of the gingipain family (C25) with its catalytic domain sharing 20% identity with the catalytic domain of RgpB including the conserved Cys/His catalytic dyad [19]. Sortases are cysteine proteinases of the peptidase family C60 and in addition to the catalytic Cys/His dyad also have a conserved Arg residue [57]. Multiple alignment of PG0026 (PorU) orthologs also reveals a conserved Arg residue (Arg 722), only 32 residues C-terminal to the catalytic Cys (S9 Fig). This conserved Arg is not present in the Arg- and Lys-specific gingipains. PG0026 is therefore the best candidate to be the sortase-like protein in *P*. *gingivalis*, its active site is presumably a little different to the gingipain proteinases such that it prefers an amine as the nucleophile rather than water.

**Fig 12:**
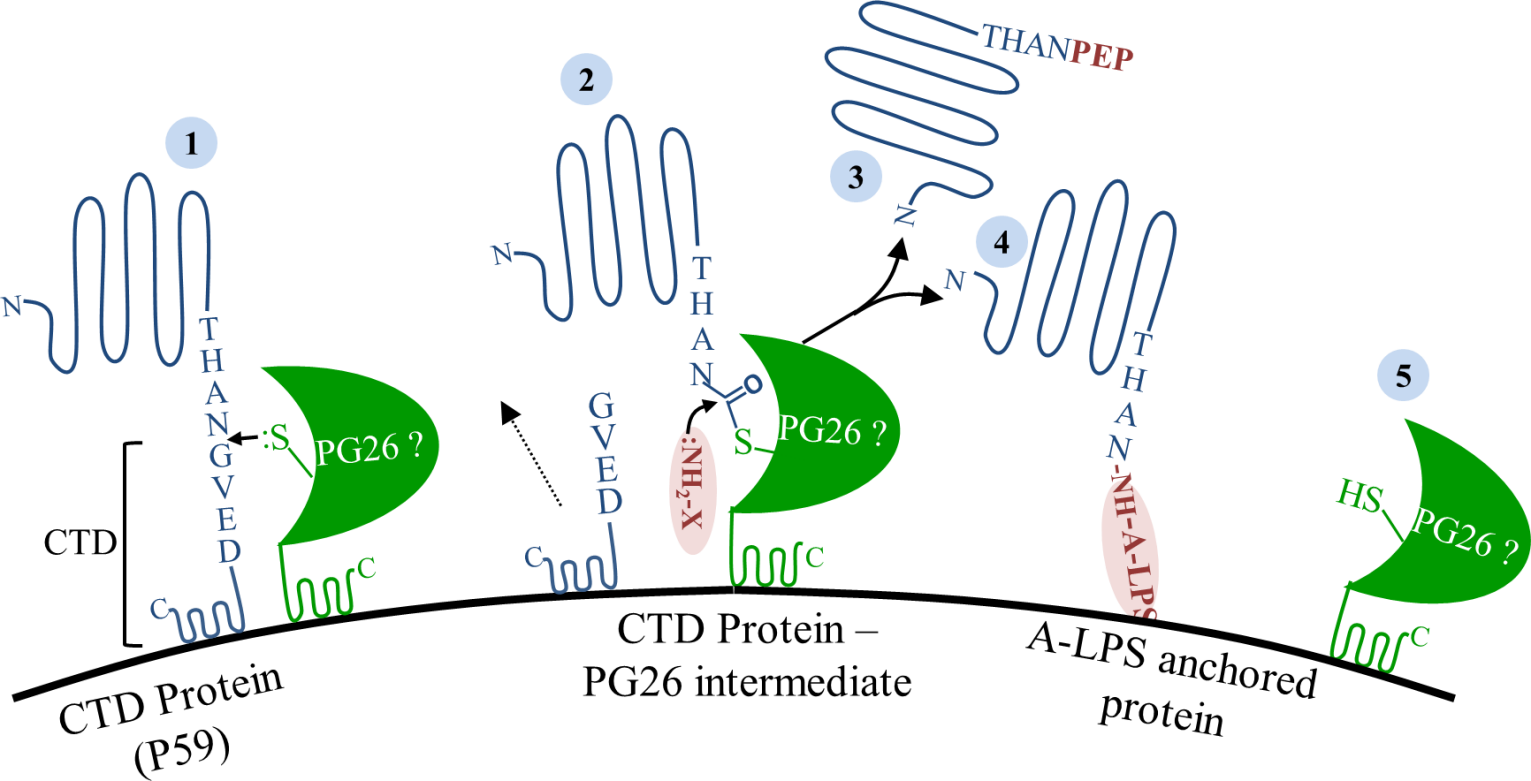
Model of sortase-like mechanism in *P*. *gingivalis*. CTD proteins translocate to the cell surface via the T9SS. (1) On the surface, proposed sortase PG0026 cleaves the CTD peptide, (2) resulting in the formation of a thioester enzyme intermediate and release of cleaved CTD peptide into the culture fluid. (3) In the absence of A-LPS, nucleophilic attack from the free amino group of peptides in the growth media resolves the acyl-enzyme intermediate and releases CTD protein modified with peptide into the culture fluid. (4) In the presence of A-LPS, nucleophilic attack from the free amino group on the A-LPS at the thioester bond resolves the acyl-enzyme intermediate and synthesizes an amide bond between CTD proteins and A-LPS resulting in the anchorage of CTD proteins to the cell surface. (5) Active site of PG0026 is regenerated.

Fig 12 shows a proposed model of the cleavage of CTD proteins and their attachment to the cell surface by the putative PG0026 sortase in *P*. *gingivalis*. It is proposed that PG0026 cleaves the CTD and forms an intermediate acyl-enzyme with the mature C-terminus of the CTD protein. This intermediate is then resolved by nucleophilic attack from a free amine present in A-LPS thereby attaching the CTD proteins to the cell membrane (Fig 12). In the absence of A-LPS, the acyl-enzyme intermediate is attacked by a free amine from the growth medium thereby releasing the CTD proteins into the culture fluid (Fig 12). The acyl-enzyme intermediate can also be resolved by hydrolysis with water and while this hydrolysis was observed for CPG70, P59 and P27 the preferred nucleophile was an amine. In the wild type, the level of hydrolysis would be even less, since the natural substrate is available explaining why a high proportion of gingipain activity is cell or vesicle associated in wild type strains such as W50 and 33277 [9, 19, 21].

In conclusion, we have for the first time localized a common modification of CTD proteins which is likely to be a portion of A-LPS. The modification is localised to the mature carboxyl terminus of CTD proteins via a peptide bond which is generated after cleavage of the CTD secretion signal. In the absence of A-LPS, the mature carboxyl terminus of CTD proteins is instead linked to peptides/amino acids from the growth medium. Together, the results suggest that a sortase-like mechanism is occurring in *P*. *gingivalis* for the cleavage and modification of the CTD proteins. PG0026 is the likely candidate to be the sortase in *P*. *gingivalis*, however, this needs to be directly demonstrated. To our knowledge, this is the first biochemical evidence for a sortase-like mechanism in Gram-negative bacteria.

## Supporting Information

S1 Fig No accumulation of CTD proteins in periplasm of W50ABK*WbaP. Periplasmic fractions of LptO [21] mutant, WT and W50ABK*WbaP were separated on SDS-PAGE gel. Banding pattern in WT and W50ABK*WbaP is similar. Major bands from W50ABK*WbaP were identified as *bone-fide* periplasmic proteins [35]. Mascot scores are shown in the brackets for the identified proteins.
(TIF)

S2 Fig Absence of A-LPS in HG66 strain. Immunoblot analysis of whole cell culture lysates from WT and HG66 with anti-APS (mAb 1B5) antibody. Image on the right of shows the corresponding commassie stained gel to show loading control.
(TIF)

S3 Fig Purification of CPG70, P59 and P27 from culture fluid of W50ABK*WbaP. Culture fluid from W50ABK*WbaP was concentrated and loaded onto an anion exchange column and the fractions were analysed by SDS-PAGE. P59 and P27 did not require further purification. For CPG70, the fractions containing CPG70 from anion exchange were further purified using a gel filtration column. Fractions were analysed by SDS-PAGE.
(TIF)

S4 Fig MS/MS spectra of modified C-terminal peptides of Pro-CPG70 and P59. Purified CPG70 and P59 from W50ABK*WbaP was subjected to in-solution digest with trypsin and the tryptic fragments were analysed with LC-MS/MS (Orbitrap). MS/MS spectra of C-terminal peptides of mature Pro-CPG70 and P59 showing modification at the C-terminus with various peptides.
(PDF)

S5 Fig MS/MS spectra of modified C-terminal peptides of P59 and Kgp from W50WbaP *P*. *gingivalis*. 2-D gel spots corresponding to P59 and Kgp from the CCF of W50WbaP were subjected to in-gel digestion with trypsin and the tryptic fragments were analysed with LC-MS/MS (Orbitrap). MS/MS spectra of C-terminal peptides of mature P59 and Kgp showing modification at the C-terminus with various peptides.
(PDF)

S6 Fig Q-Sepharose chromatography of modified CTD proteins. Proteins solubilised in 1% Zwittergent were separated by anion exchange chromatography using a Q-Sepharose column equilibrated in 20 mM Bis-Tris/5 mM CaCl_2_/50 mM NaCl/0.05% Zwittergent, pH 6 and eluted with a linear gradient of 50–500 mM NaCl. Fractions were pooled into 5 groups as indicated.
(TIF)

S7 Fig SDS-PAGE of Q-Sepharose fractions. After in-gel digestion and MS identification of selected bands, the fractions were pooled into 5 groups, A-E as shown. RgpA_A4_ and P27 were found within the approximate region 40–65 kDa, and Kgp_A5_ in the region 35–55 kDa.
(TIF)

S8 Fig Modification of CTD proteins isolated from wild type W50 with the 648 Da linker. Modified CTD proteins were semi-purified, deglycosylated with TFMS, digested in-gel with trypsin and analysed by LC-MS/MS in positive ion mode using the ion trap MS and then also by Orbitrap MS. Each panel shows the MS/MS spectrum obtained for a C-terminal trytic peptide modified with the 648 Da linker. The proteins shown are HBP35, PG1030, RgpA_A4_, Kgp_A5_, Peptidylarginine deiminase (PAD) and P27. The accurate mass of the 104 Da entity is shown for PG1030 and P27 Orbitrap MS/MS spectra. See also Fig 10.
(PDF)

S9 Fig Multiple alignment of PorU (PG0026). The sequence of PorU was obtained from the ATCC33277 strain (PGN_0022) since the N-terminal sequence of PG0026 from the W83 strain started in the wrong place [19]. 16 full length orthologs were randomly chosen to give a broad distribution across the Bacteroidetes phylum and aligned using Clustal W. The active site Cys and the conserved Arg residues are highlighted and marked with an arrow.
(PDF)

S1 Table Mass spectrometry identification of proteins in the CE of WT and W50ABK*WbaP. (XLSX)

S2 Table Mass spectrometry identification of proteins in the CCF of WT, W50ABK*WbaP and W50WbaP. (XLSX)

S3 Table Mass spectrometry identification of proteins in the WT CCF in the absence and presence of glycine. (XLSX)
