# Supplementary figures and images for: Porphyromonas gingivalis Type IX Secretion Substrates Are Cleaved and Modified by a Sortase-Like Mechanism
Source: PLoS Pathog. 2015 Sep 4;11(9):e1005152. doi: 10.1371/journal.ppat.1005152 (PMC4560394; doi:10.1371/journal.ppat.1005152)

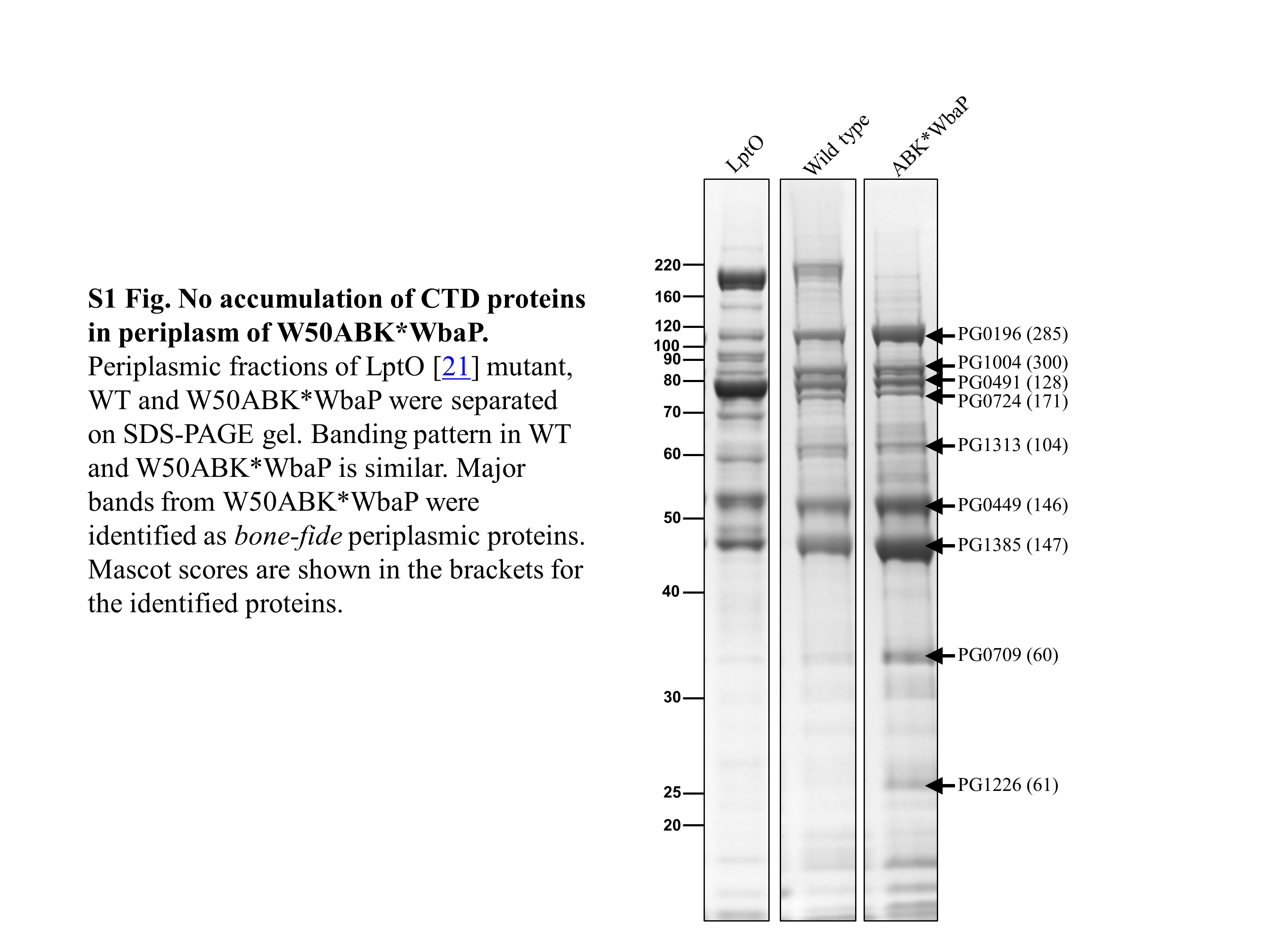

Supplement: S1 Fig — Periplasmic fractions of LptO [21] mutant, WT and W50ABK*WbaP were separated on SDS-PAGE gel. Banding pattern in WT and W50ABK*WbaP is similar. Major bands from W50ABK*WbaP were identified as bone-fide periplasmic proteins [35]. Mascot scores are shown in the brackets for the identified proteins. (TIF) [file ppat.1005152.s001.TIF]

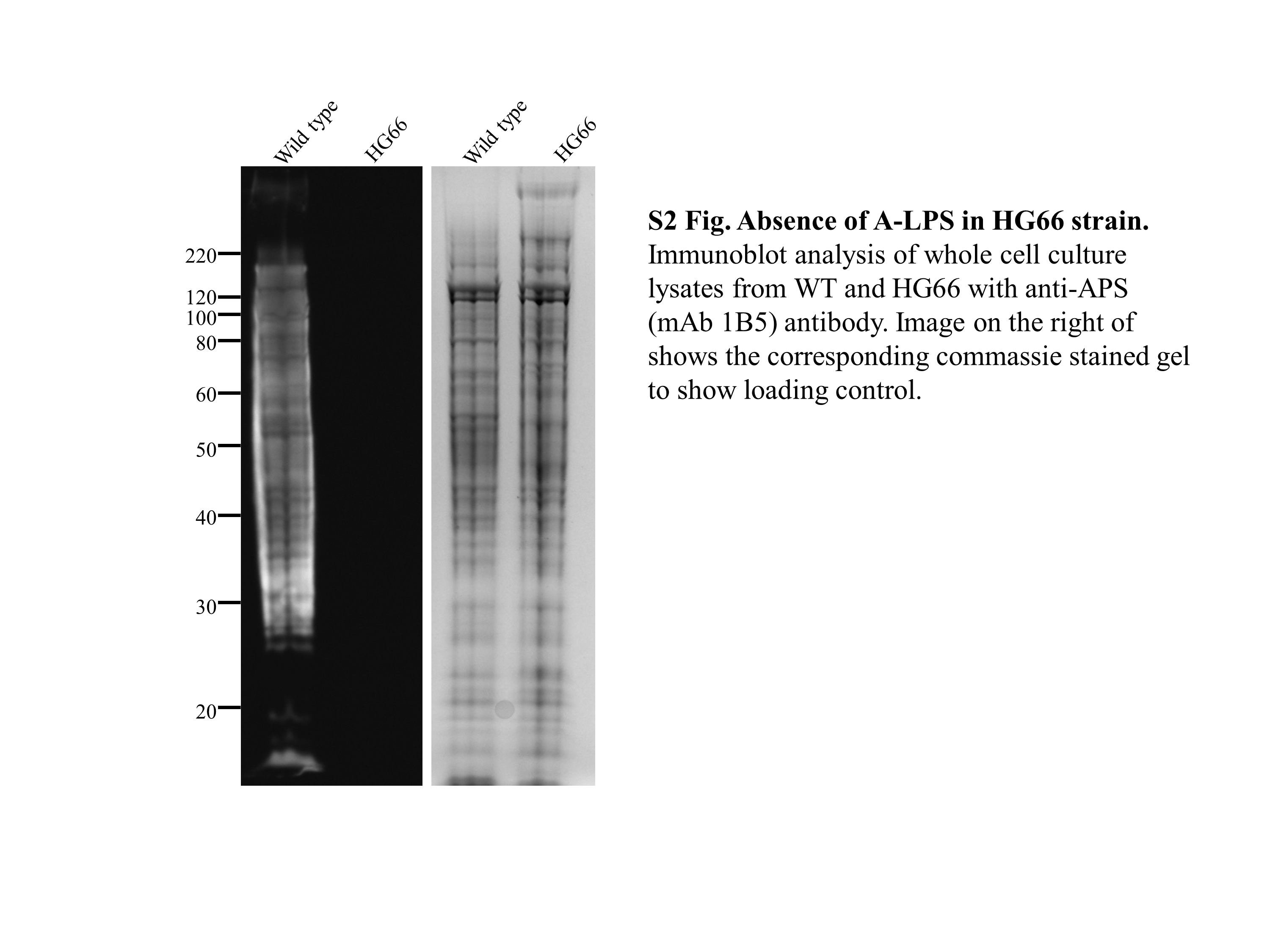

Supplement: S2 Fig — Immunoblot analysis of whole cell culture lysates from WT and HG66 with anti-APS (mAb 1B5) antibody. Image on the right of shows the corresponding commassie stained gel to show loading control. (TIF) [file ppat.1005152.s002.TIF]

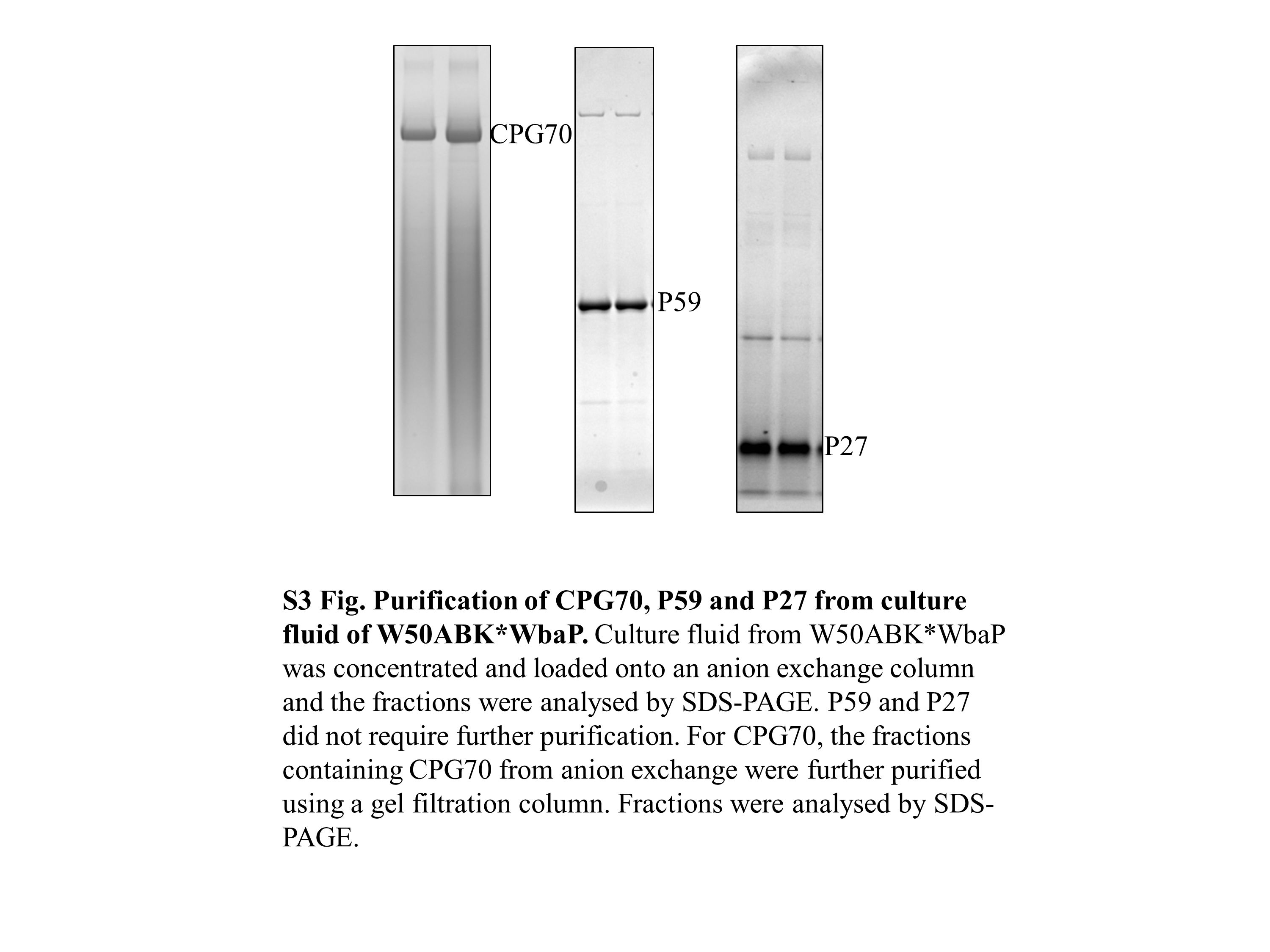

Supplement: S3 Fig — Culture fluid from W50ABK*WbaP was concentrated and loaded onto an anion exchange column and the fractions were analysed by SDS-PAGE. P59 and P27 did not require further purification. For CPG70, the fractions containing CPG70 from anion exchange were further purified using a gel filtration column. Fractions were analysed by SDS-PAGE. (TIF) [file ppat.1005152.s003.TIF]

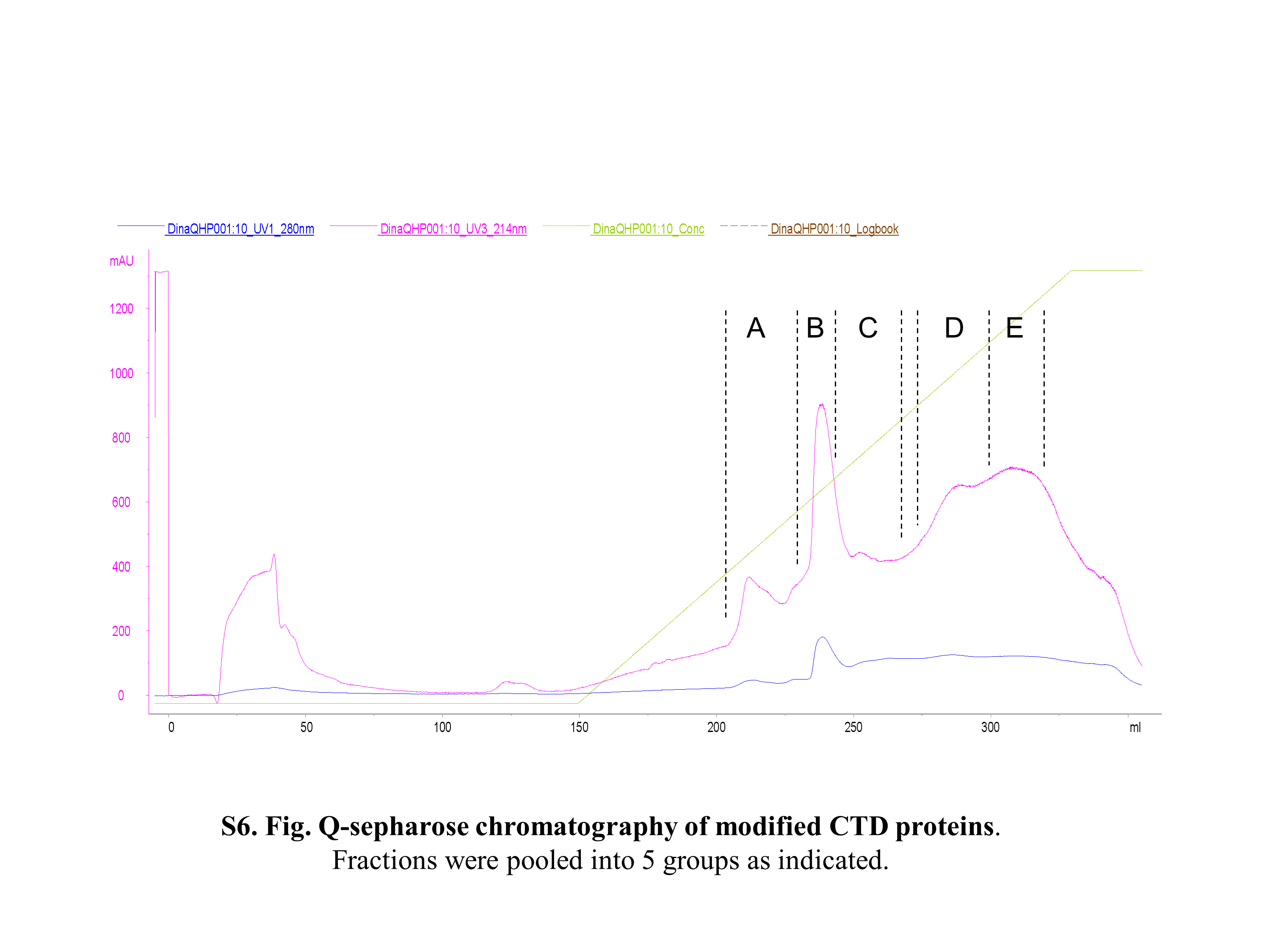

Supplement: S6 Fig — Proteins solubilised in 1% Zwittergent were separated by anion exchange chromatography using a Q-Sepharose column equilibrated in 20 mM Bis-Tris/5 mM CaCl2/50 mM NaCl/0.05% Zwittergent, pH 6 and eluted with a linear gradient of 50–500 mM NaCl. Fractions were pooled into 5 groups as indicated. (TIF) [file ppat.1005152.s006.TIF]

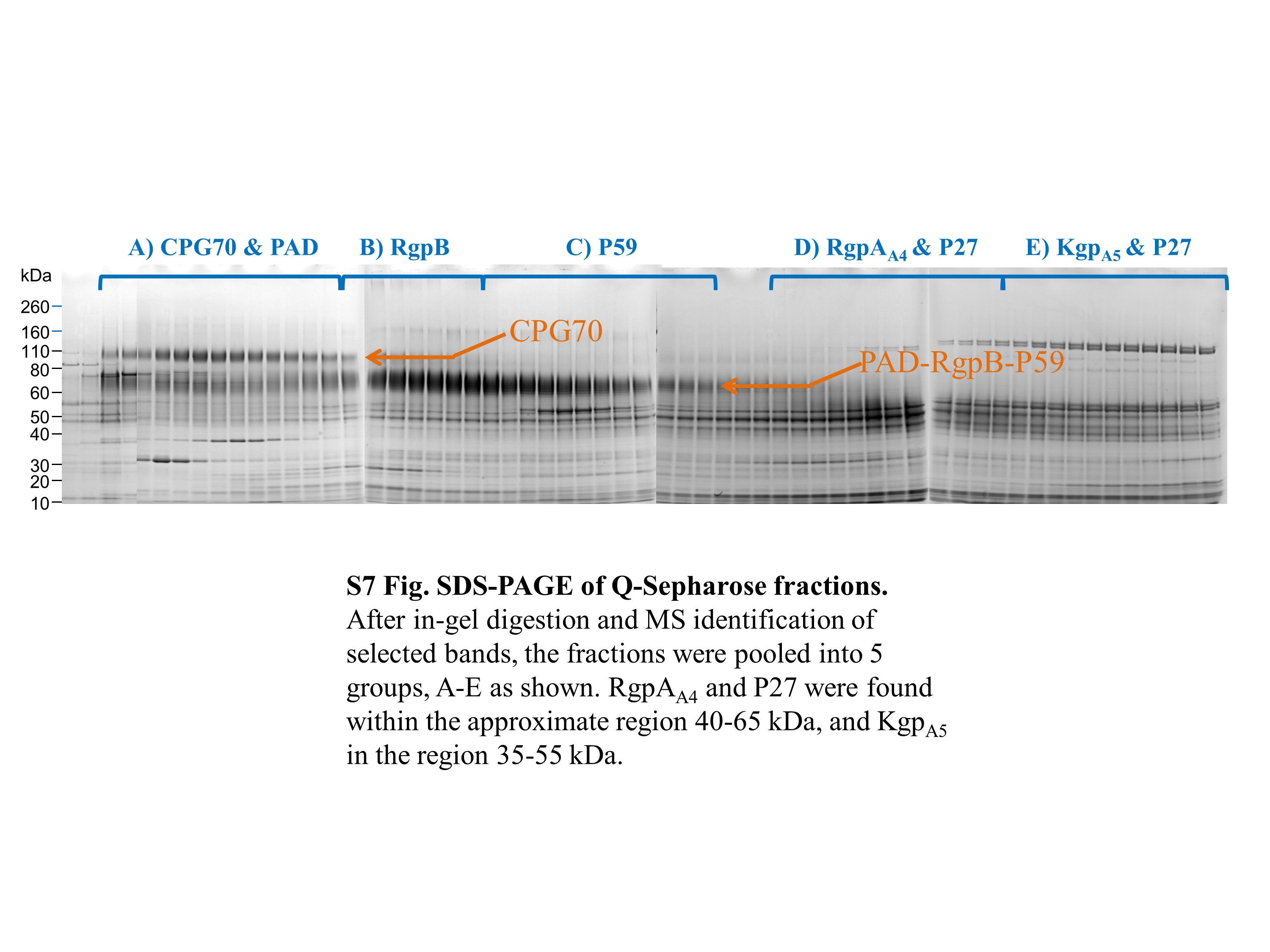

Supplement: S7 Fig — After in-gel digestion and MS identification of selected bands, the fractions were pooled into 5 groups, A-E as shown. RgpAA4 and P27 were found within the approximate region 40–65 kDa, and KgpA5 in the region 35–55 kDa. (TIF) [file ppat.1005152.s007.TIF]
