# Supplementary material for: Porphyromonas gingivalis Type IX Secretion Substrates Are Cleaved and Modified by a Sortase-Like Mechanism
Source: PLoS Pathog. 2015 Sep 4;11(9):e1005152. doi: 10.1371/journal.ppat.1005152 (PMC4560394; doi:10.1371/journal.ppat.1005152)

Supplementary Fig. 4

MS/MS spectra of modified C-terminal peptides of CPG70 and P59

KAEDYIEVILDD (unmodified CPG70 C-terminal peptide)

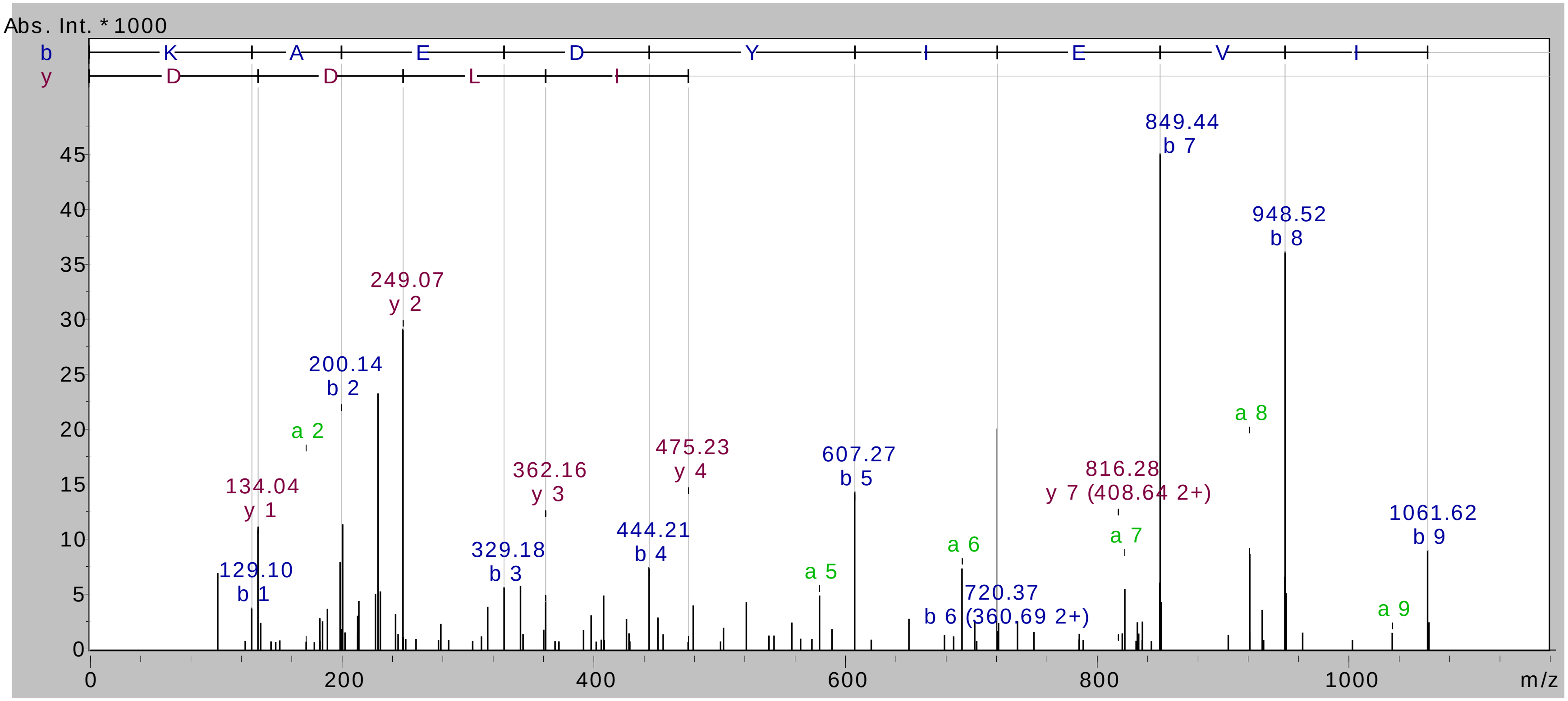

KAEDYIEVILDDG(VQ)

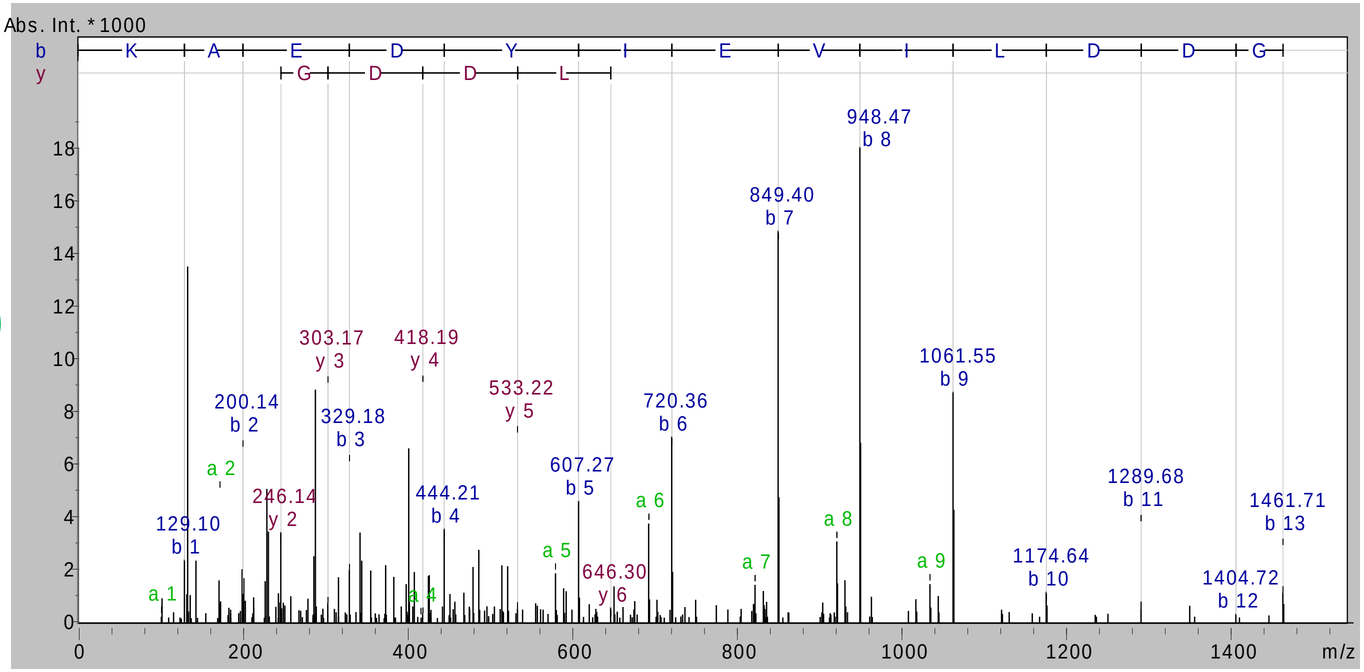

KAEDYIEVILDDGVS

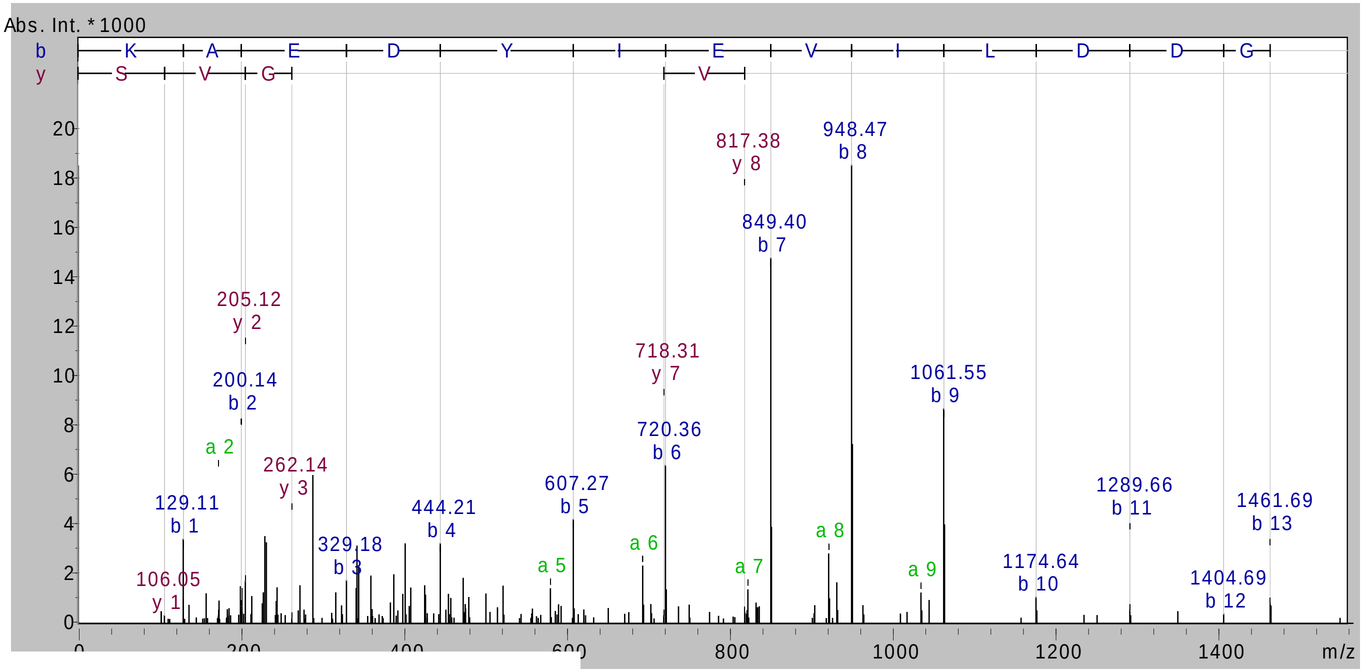

KAEDYIEVILDDGTQ

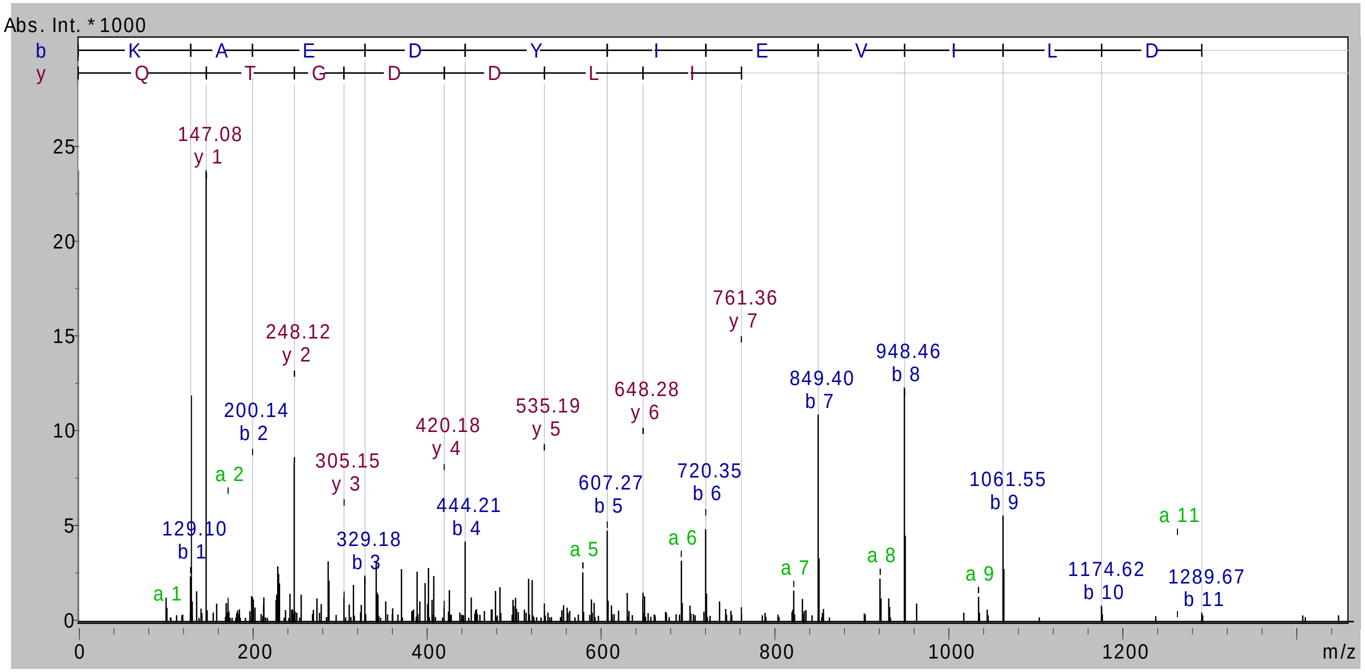

KAEDYIEVILDDLPQ

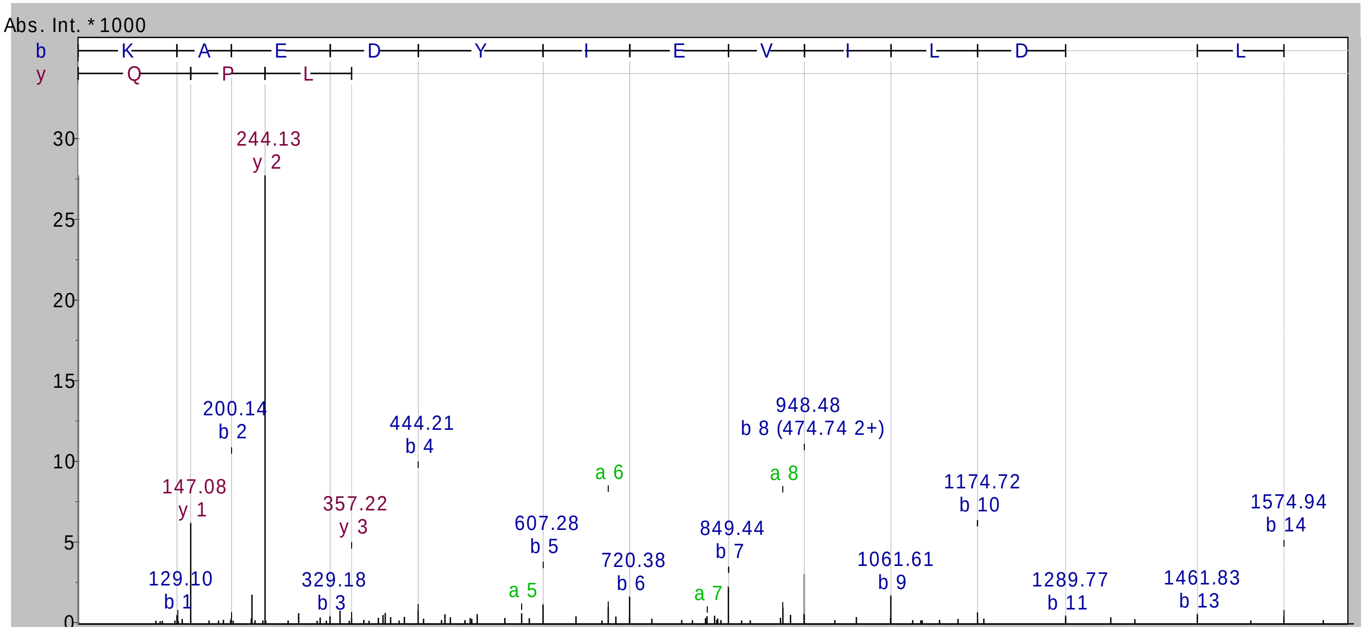

KAEDYIEVILDDGLP

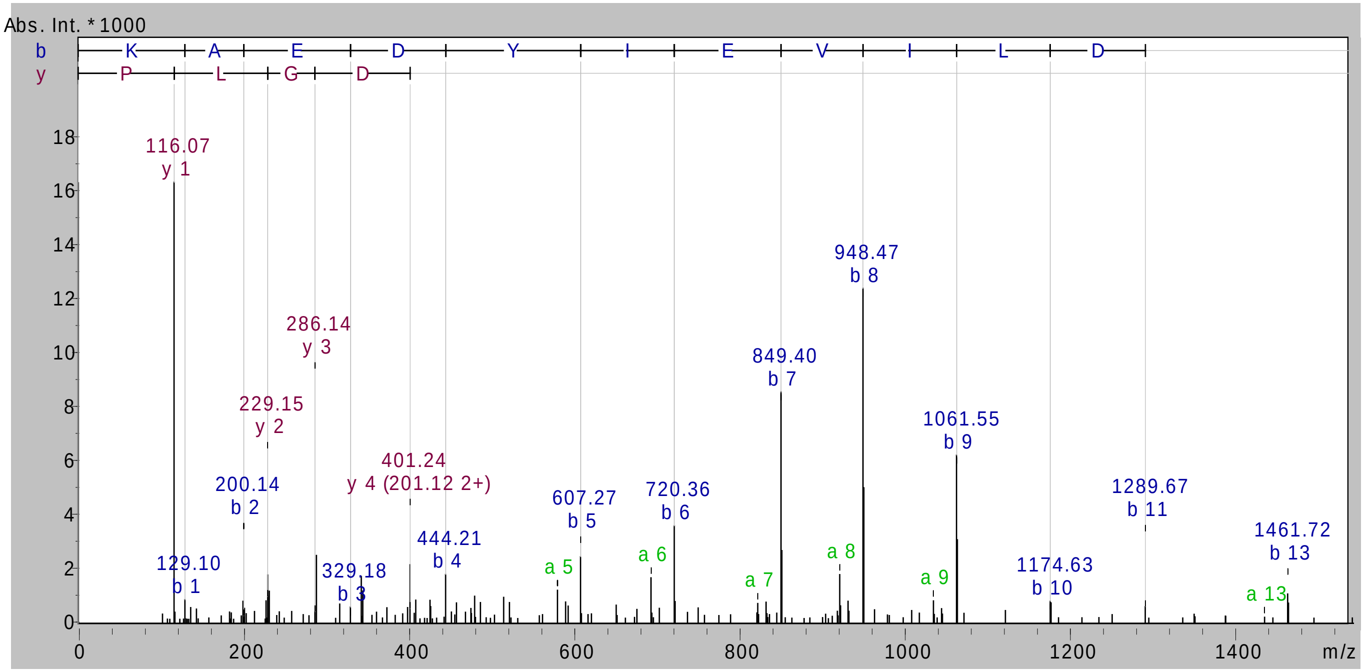

KAEDYIEVILDDQLH

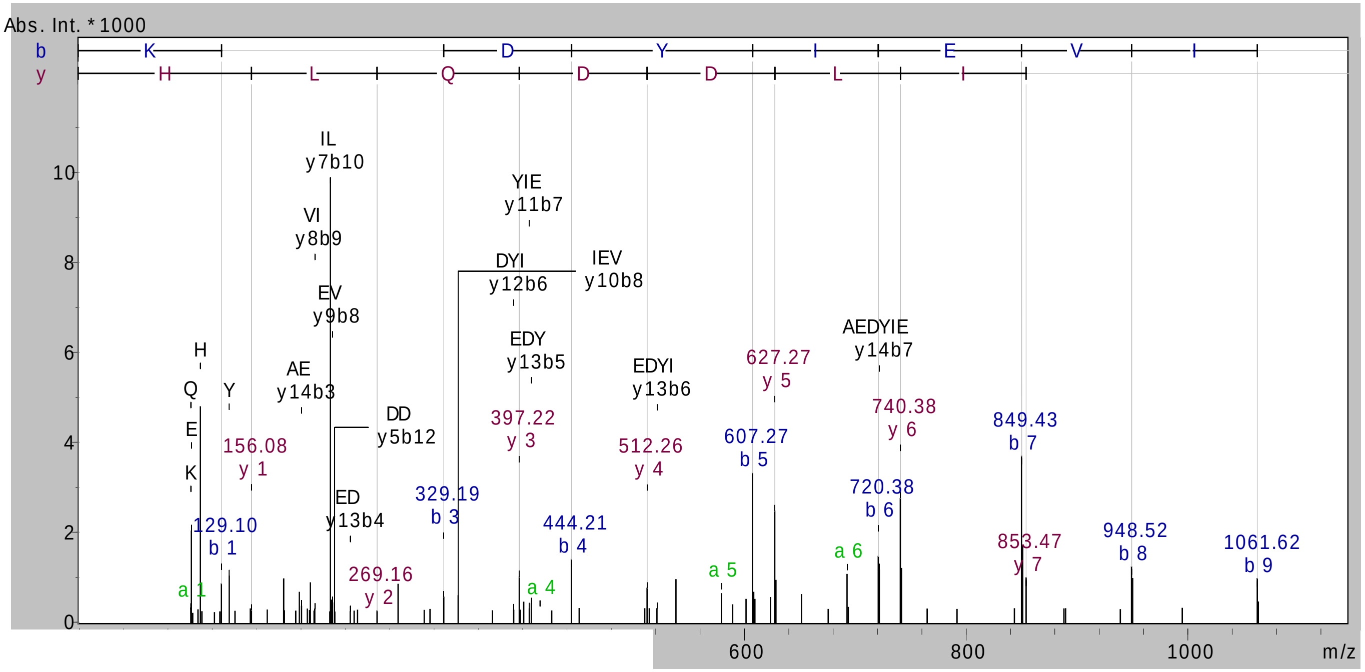

KAEDYIEVILDDS(VT)

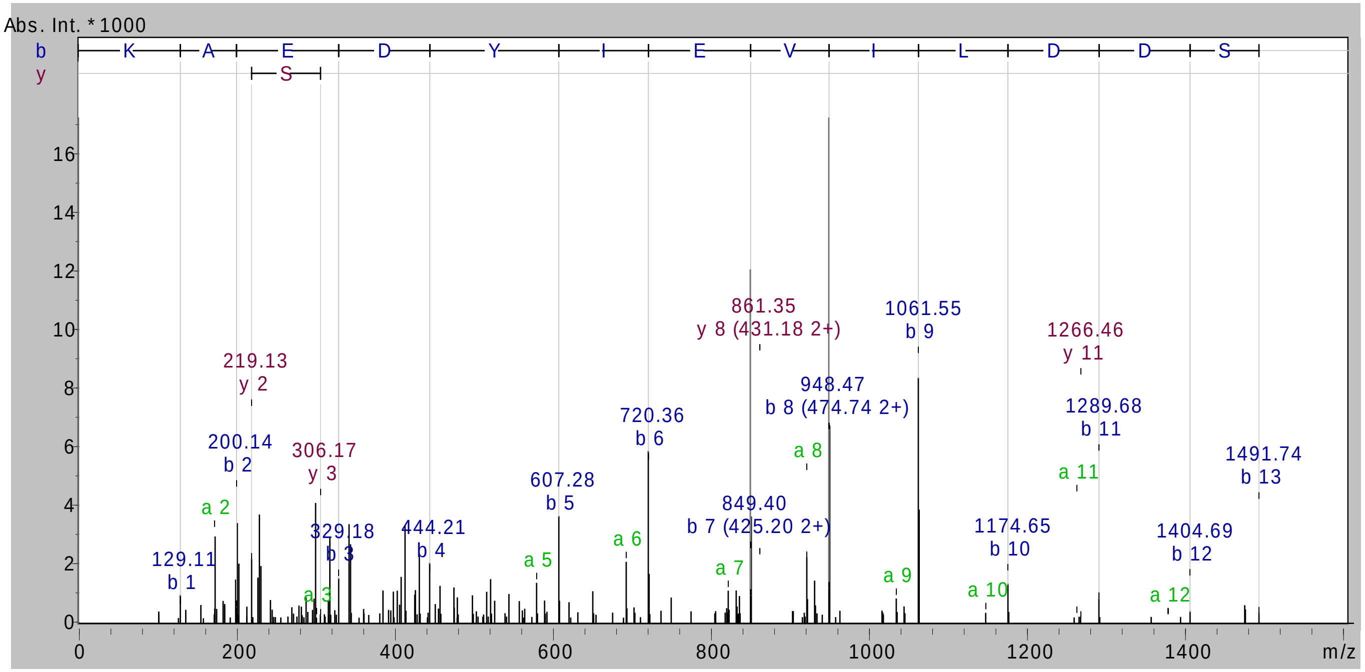

KAEDYIEVILDD(GMWK)

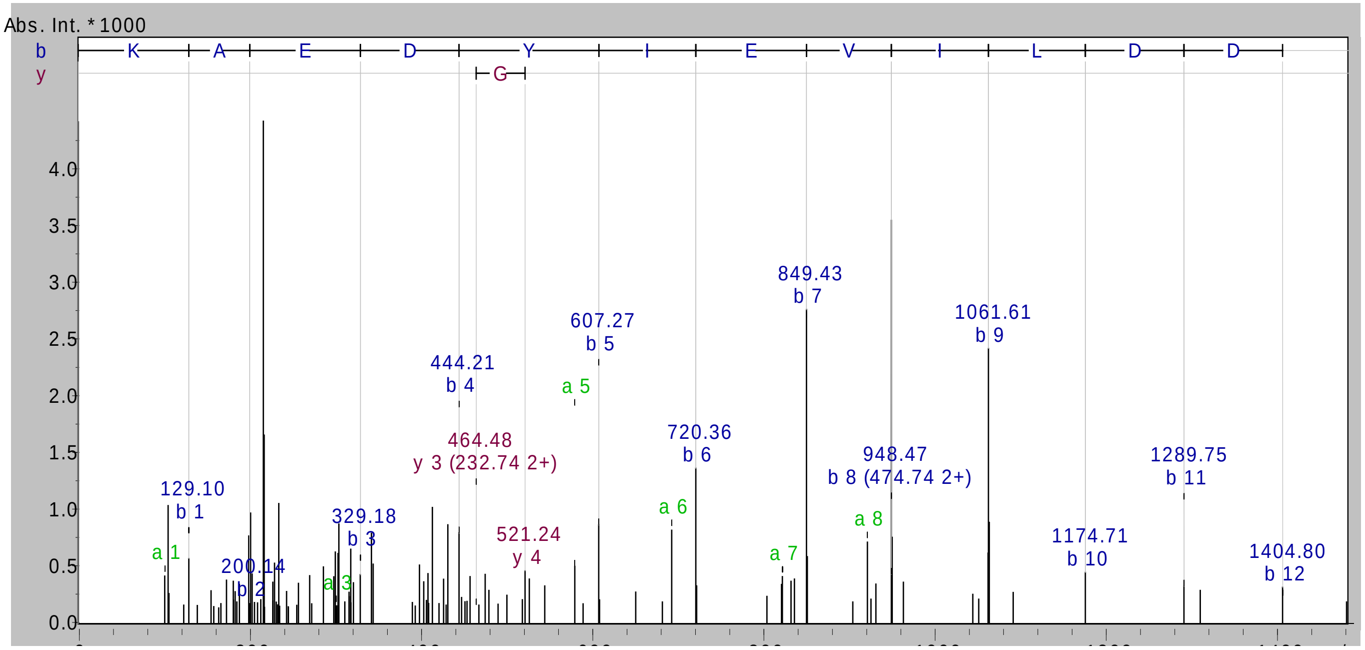

KAEDYIEVILDDSLPQ

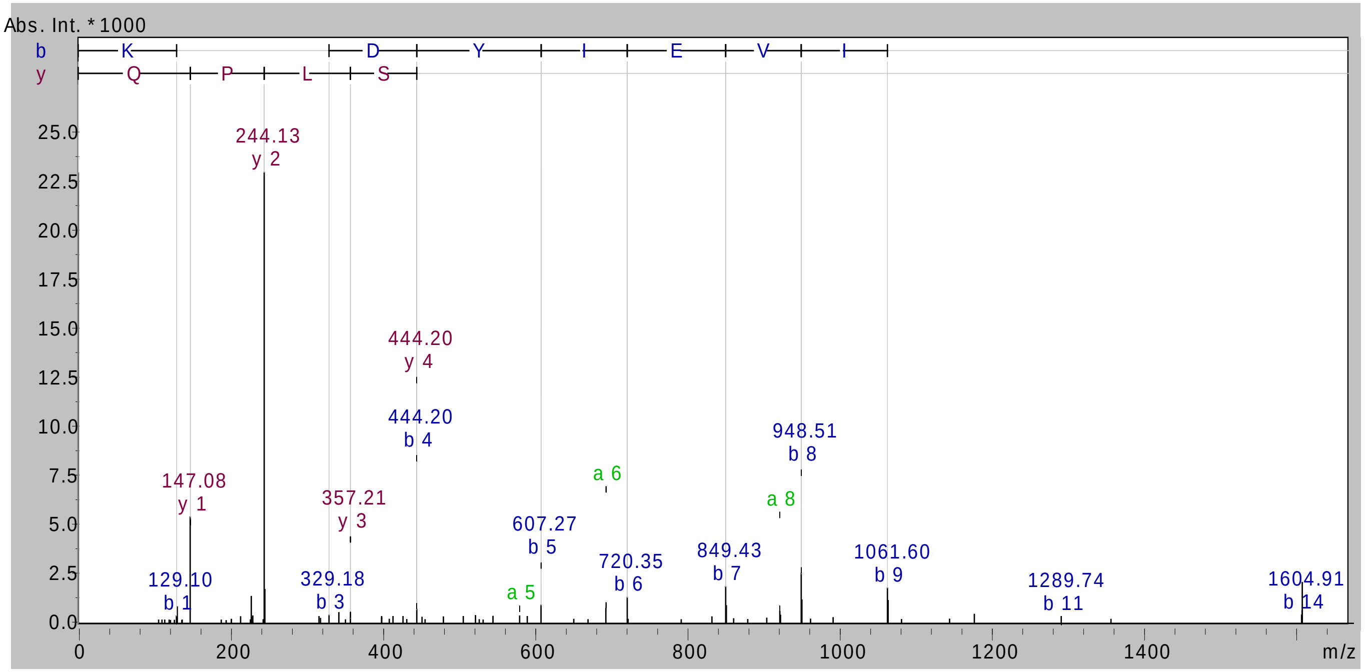

KAEDYIEVILDDK

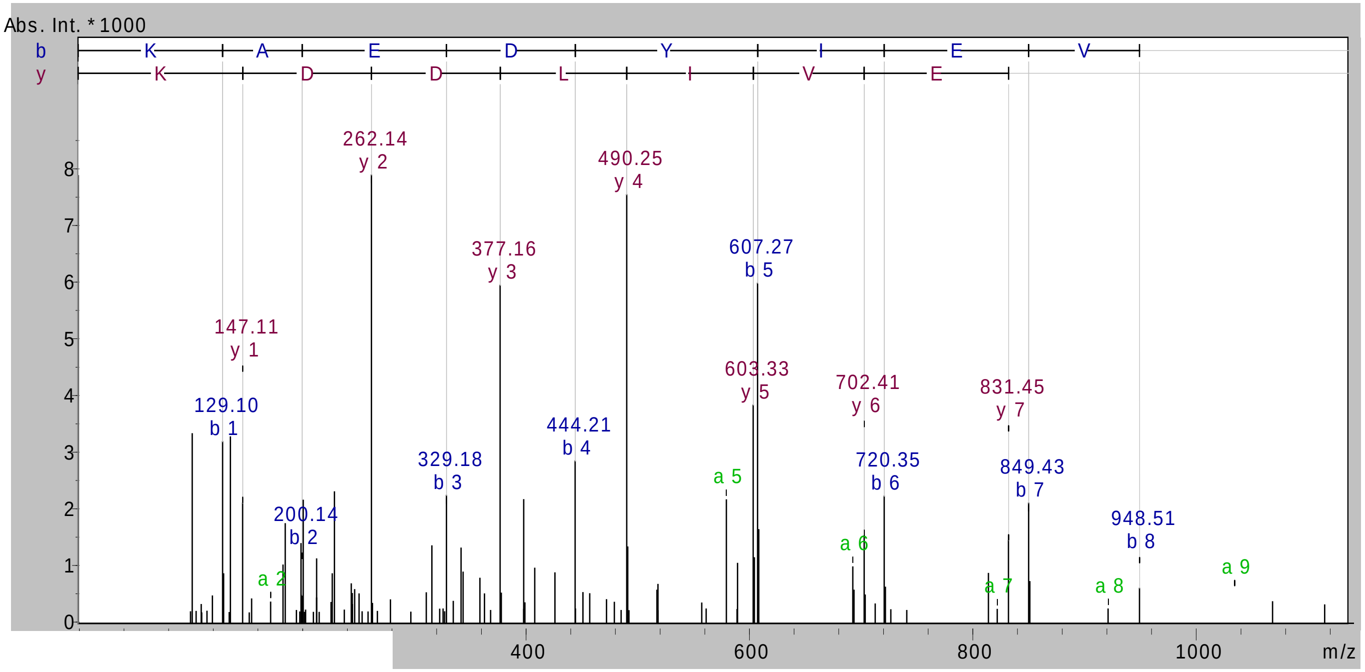

KAEDYIEVILDDR

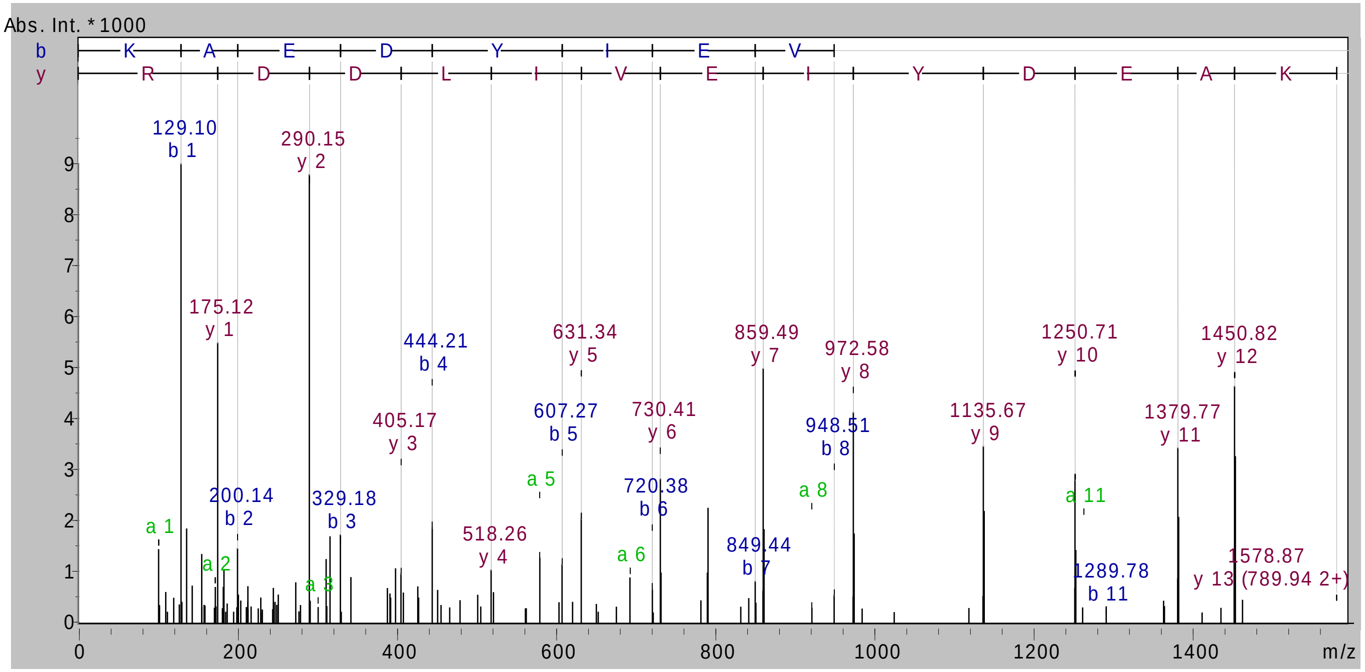

KAEDYIEVILDD-NH_2

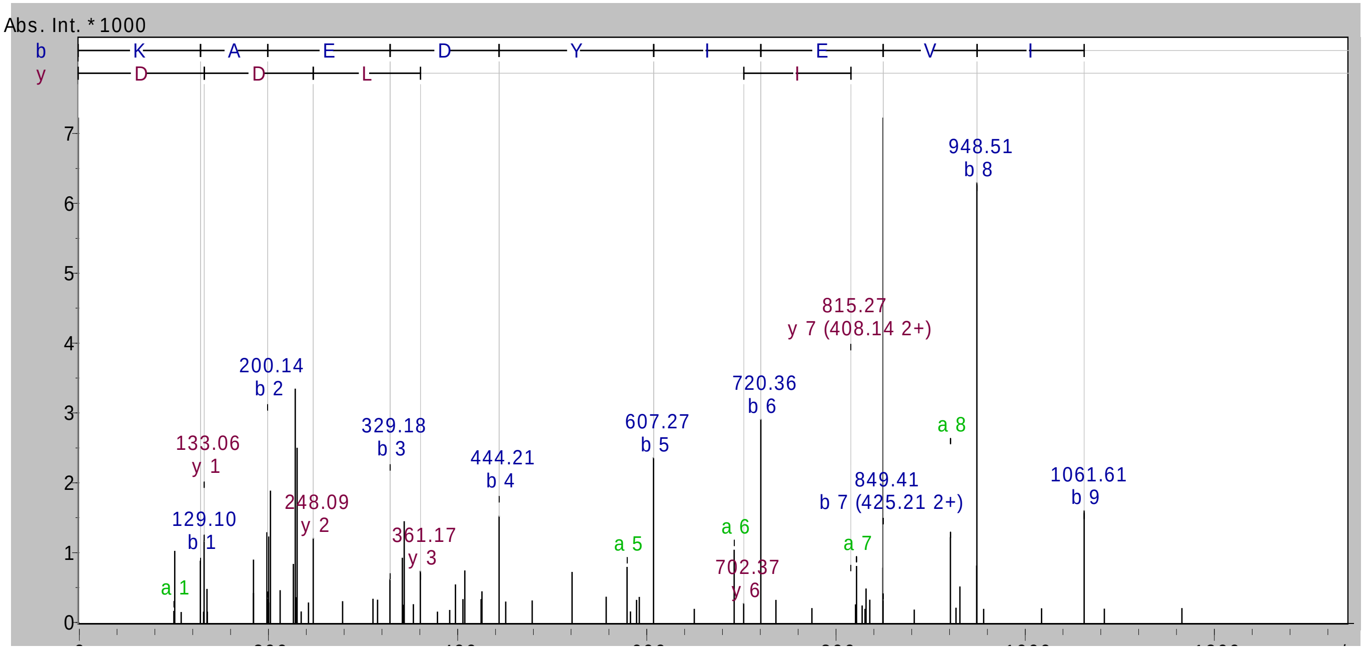

P59 C-TERMINAL PEPTIDES

IVWSDTQWTHAN (unmodified)

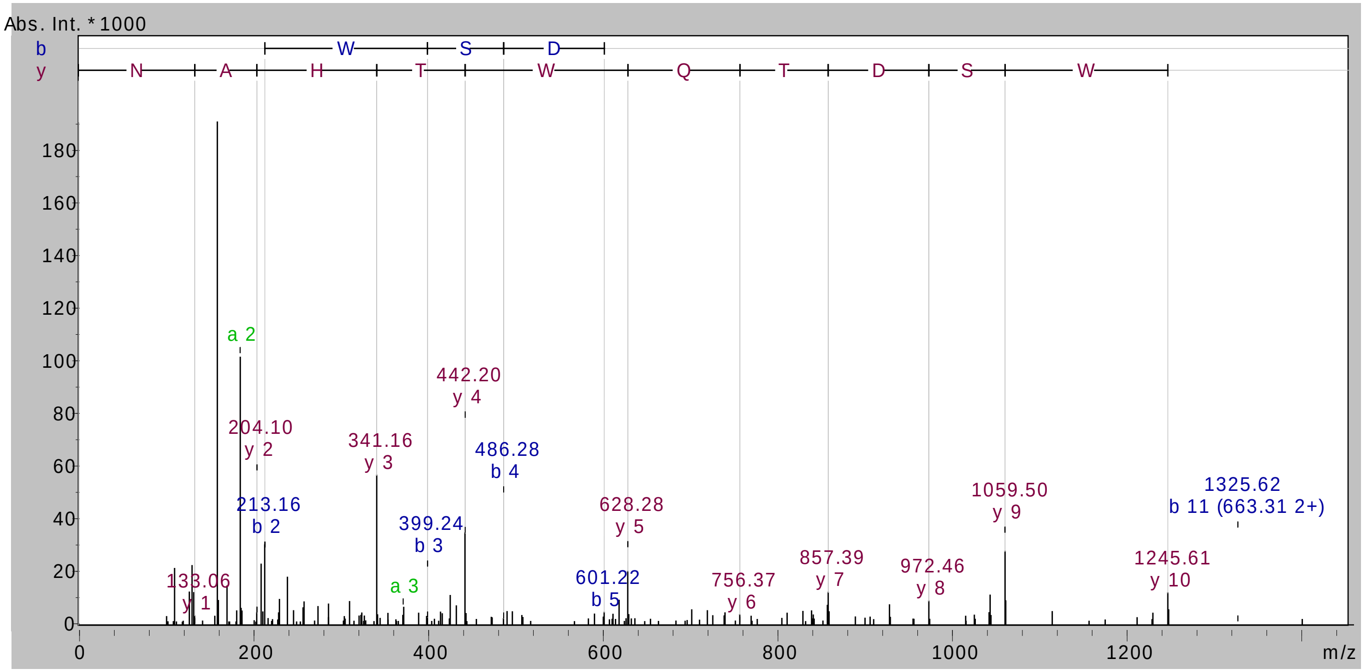

IVWSDTQWTHANG(VQ)

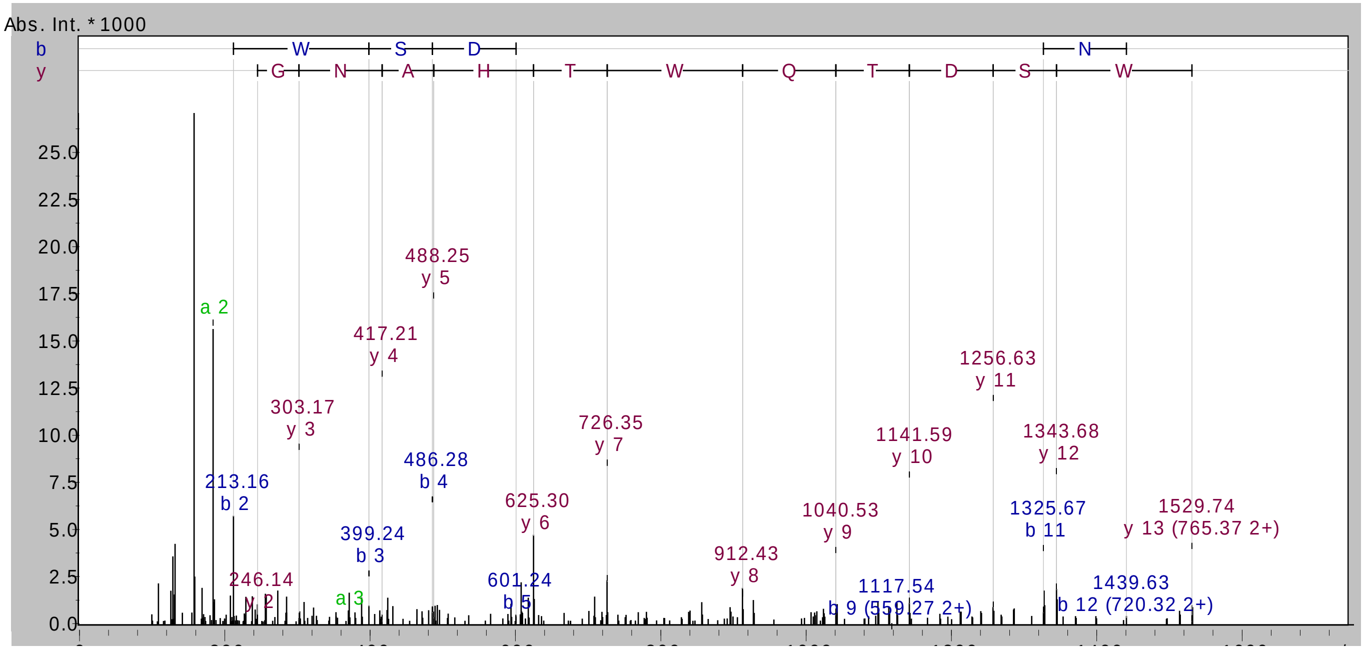

IVWSDTQWTHANGVS

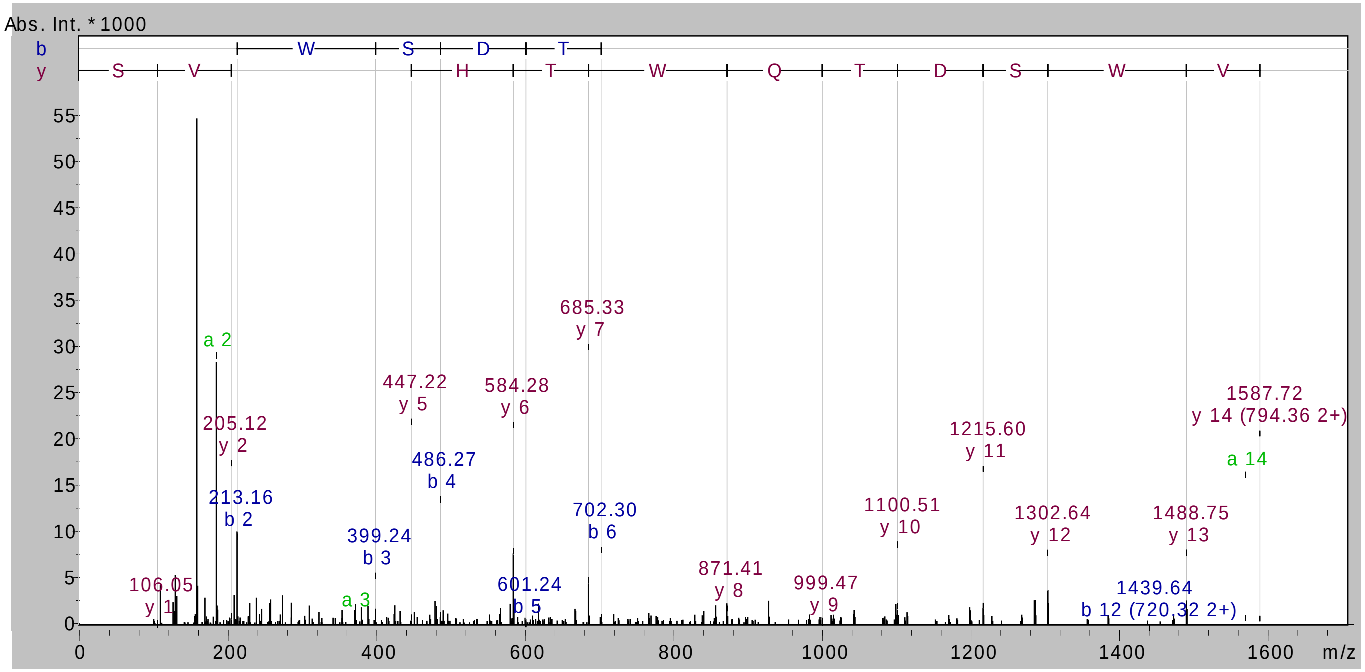

IVWSDTQWTHANR

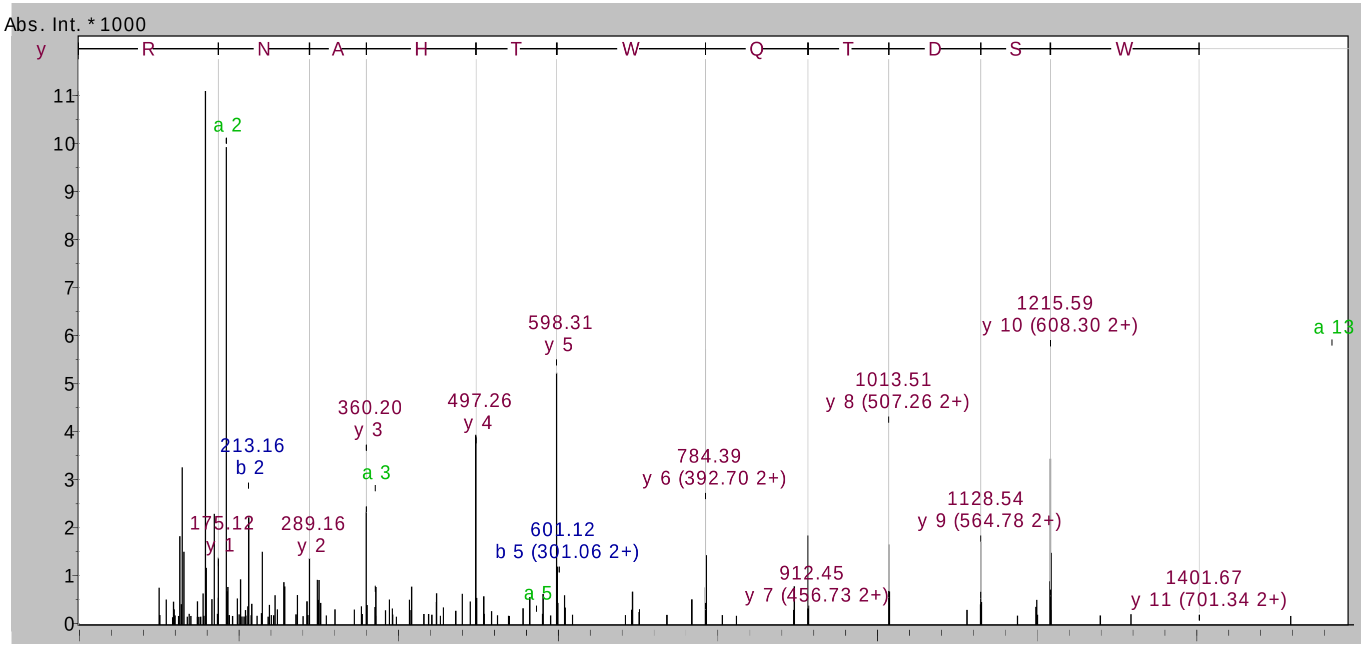

Supplement: S4 Fig — Purified CPG70 and P59 from W50ABK*WbaP was subjected to in-solution digest with trypsin and the tryptic fragments were analysed with LC-MS/MS (Orbitrap). MS/MS spectra of C-terminal peptides of mature Pro-CPG70 and P59 showing modification at the C-terminus with various peptides. (PDF) [file ppat.1005152.s004.pdf]
