# Supplementary material for: Porphyromonas gingivalis Type IX Secretion Substrates Are Cleaved and Modified by a Sortase-Like Mechanism
Source: PLoS Pathog. 2015 Sep 4;11(9):e1005152. doi: 10.1371/journal.ppat.1005152 (PMC4560394; doi:10.1371/journal.ppat.1005152)

Supplementary Fig. 5

# MS/MS spectra of modified C-terminal peptides of P59 and Kgp from W50WbaP *P. gingivalis* strain

# (P59)IVWSDTQWTHANGVK

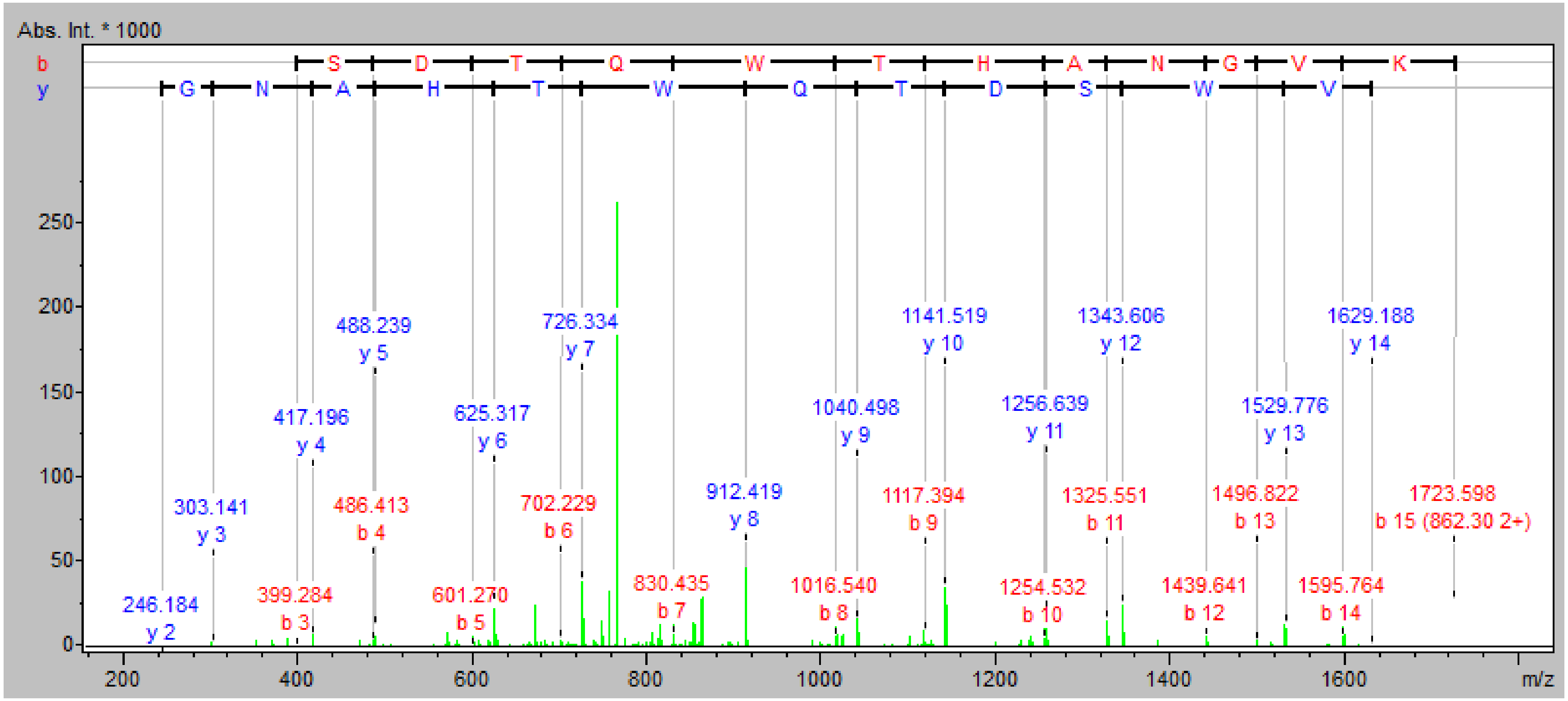

# (P59)IVWSDTQWTHANGI

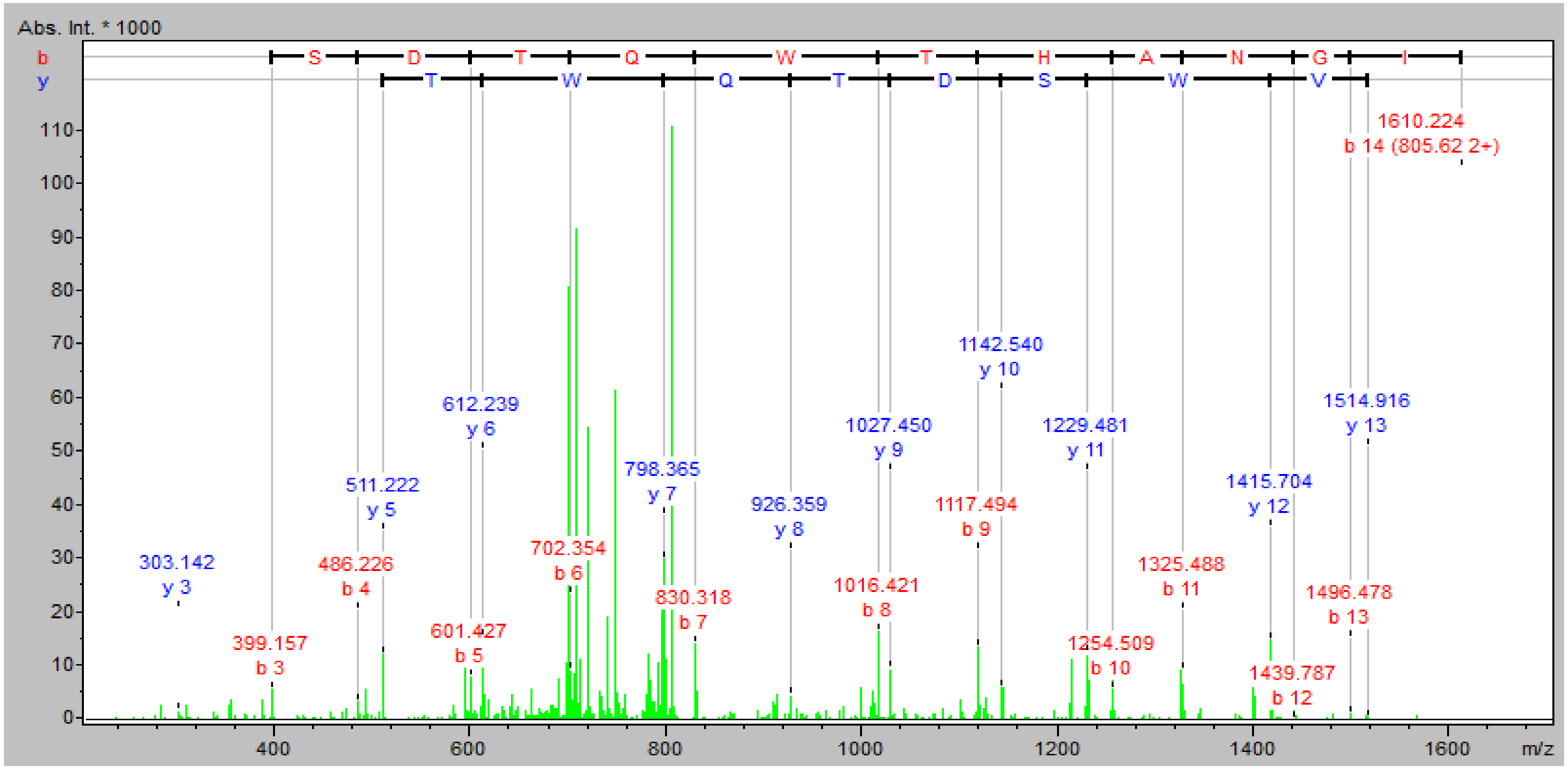

# (P59)IVWSDTQWTHANGF

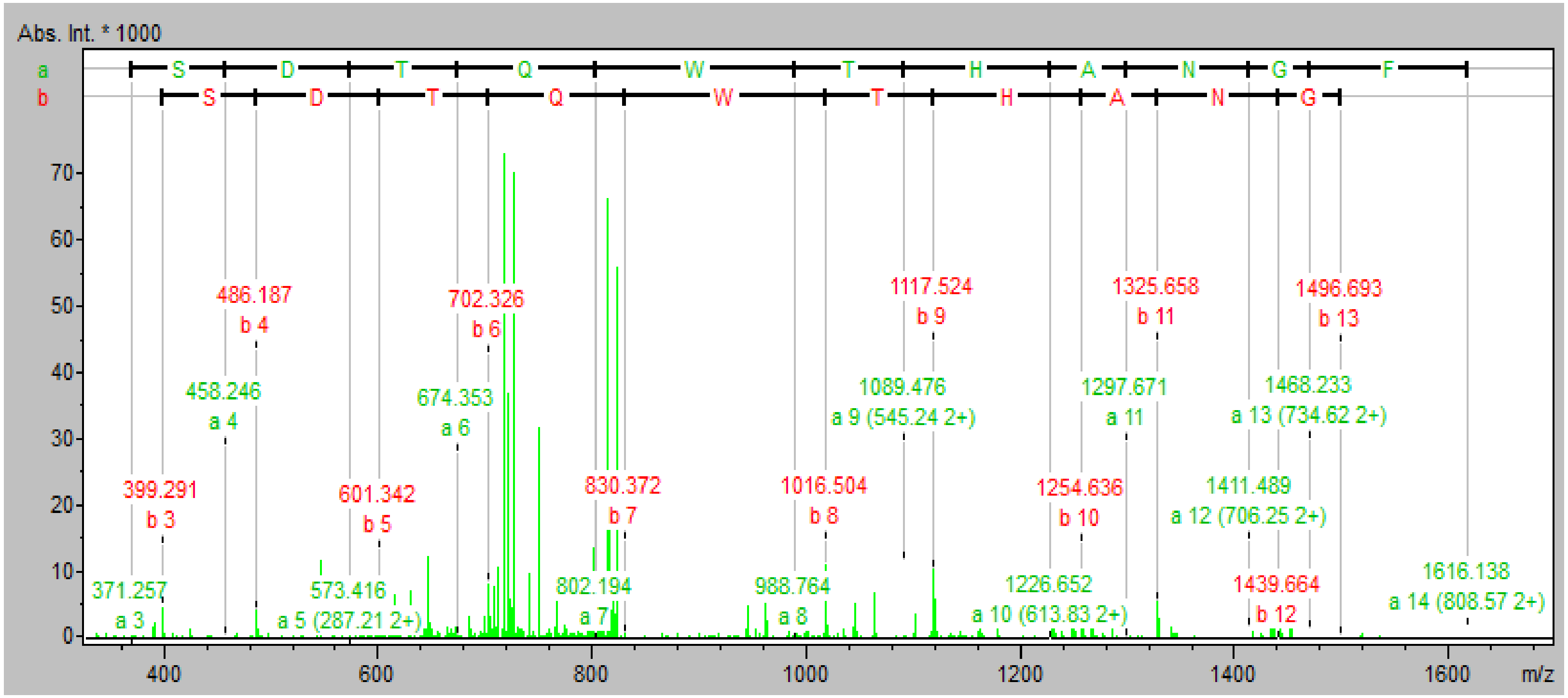

(Kgp)VVYPNGESAIETATLNITSK

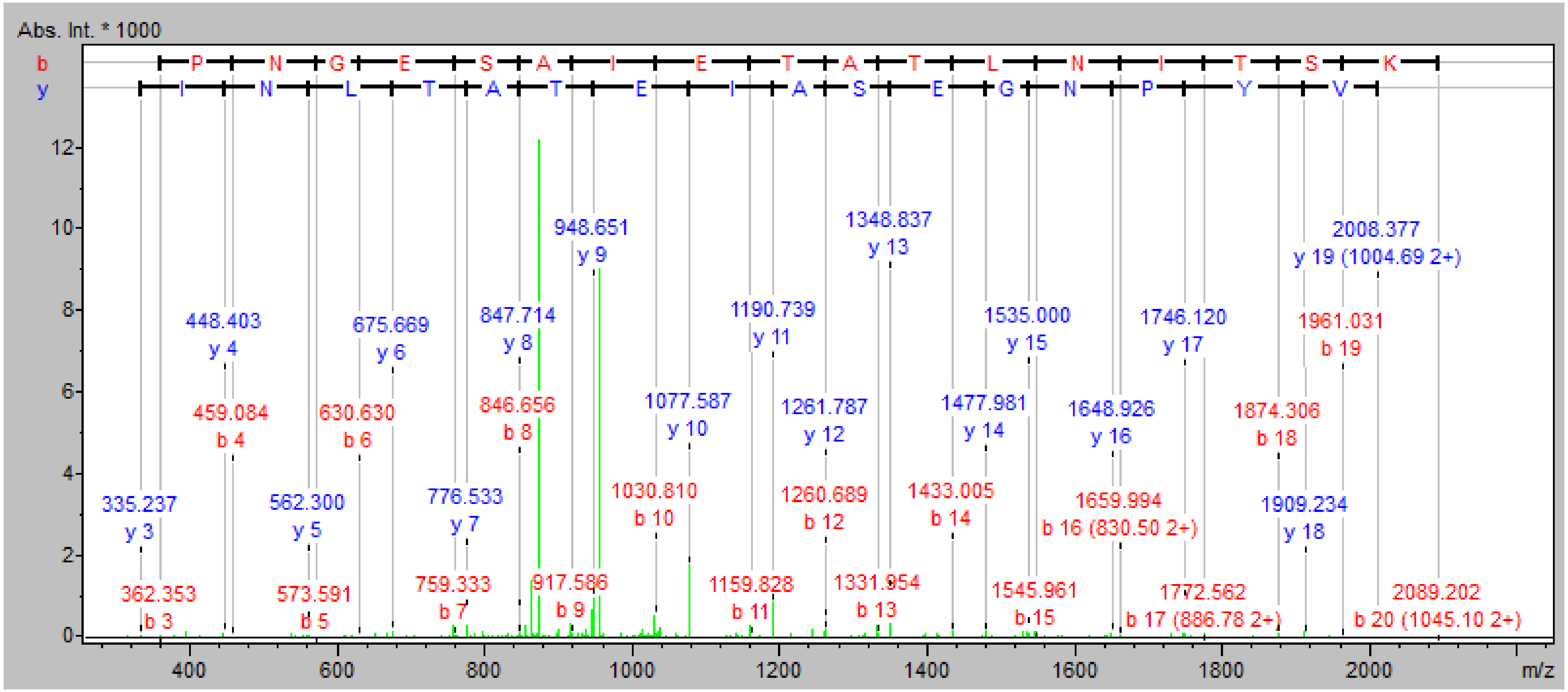

(Kgp)VVYPNGESAIETATLNIT<u>R</u>

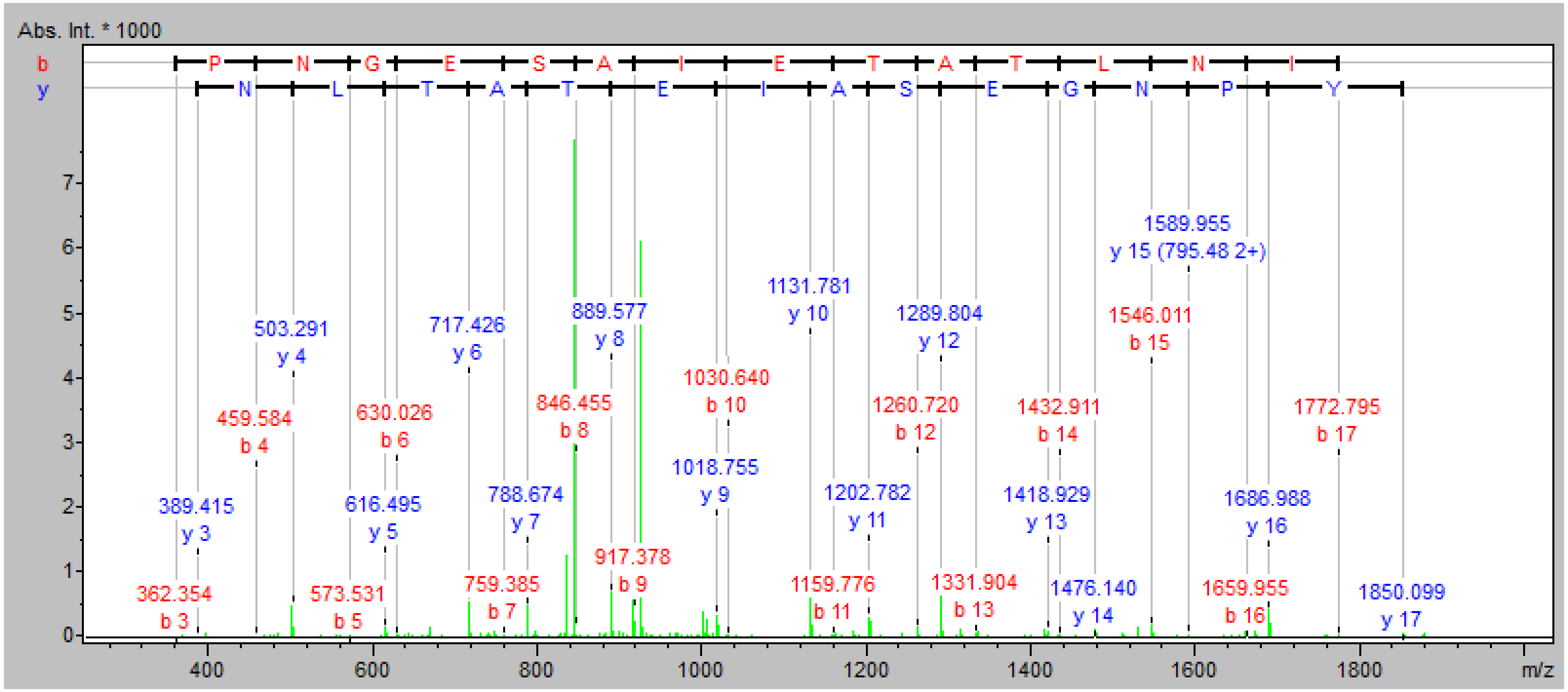

(Kgp)VVYPNGESAIETATLNIT<u>SR</u>

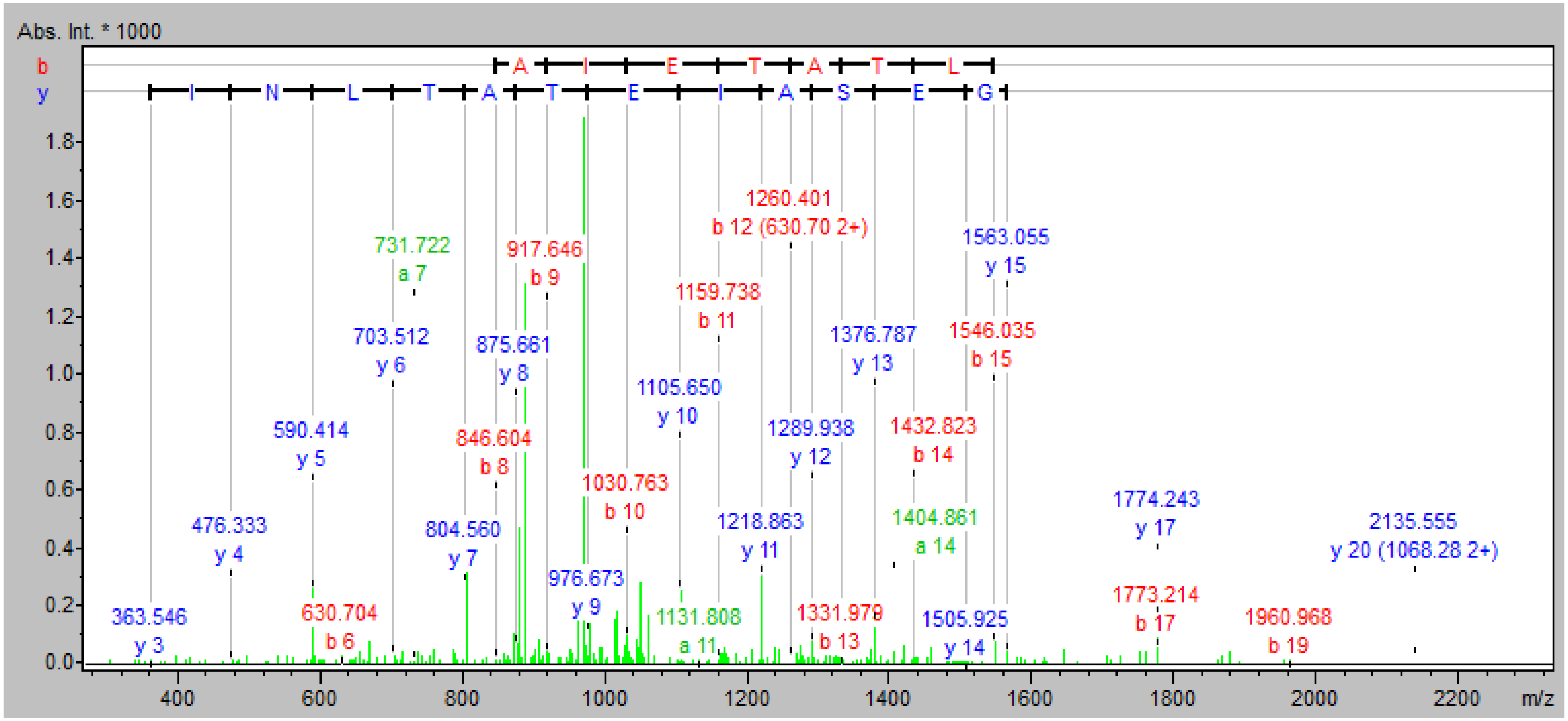

(Kgp)VVYPNGESAIETATLNITGKI

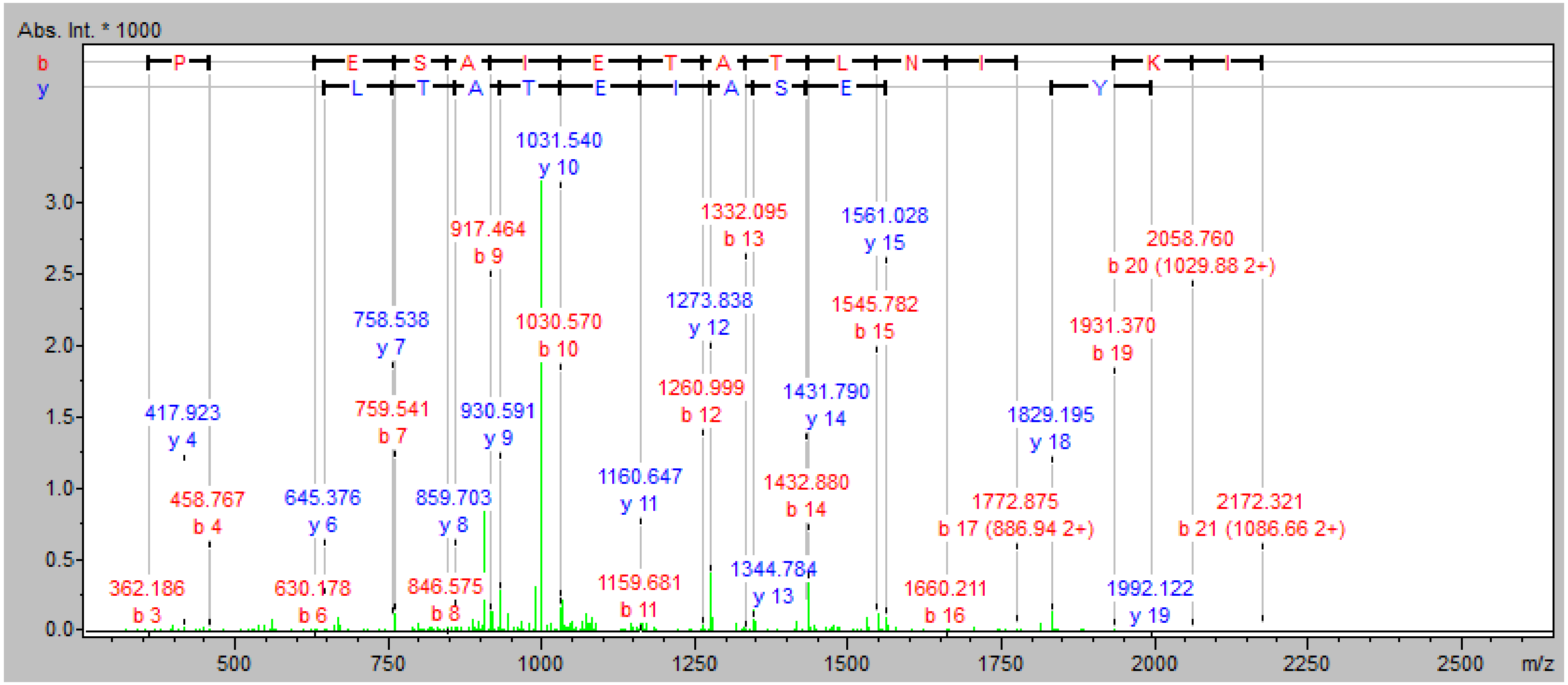

Supplement: S5 Fig — 2-D gel spots corresponding to P59 and Kgp from the CCF of W50WbaP were subjected to in-gel digestion with trypsin and the tryptic fragments were analysed with LC-MS/MS (Orbitrap). MS/MS spectra of C-terminal peptides of mature P59 and Kgp showing modification at the C-terminus with various peptides. (PDF) [file ppat.1005152.s005.pdf]
