# Supplementary material for: Porphyromonas gingivalis Type IX Secretion Substrates Are Cleaved and Modified by a Sortase-Like Mechanism
Source: PLoS Pathog. 2015 Sep 4;11(9):e1005152. doi: 10.1371/journal.ppat.1005152 (PMC4560394; doi:10.1371/journal.ppat.1005152)

Supplementary Fig. 8

HBP35: AEPT-Mod630

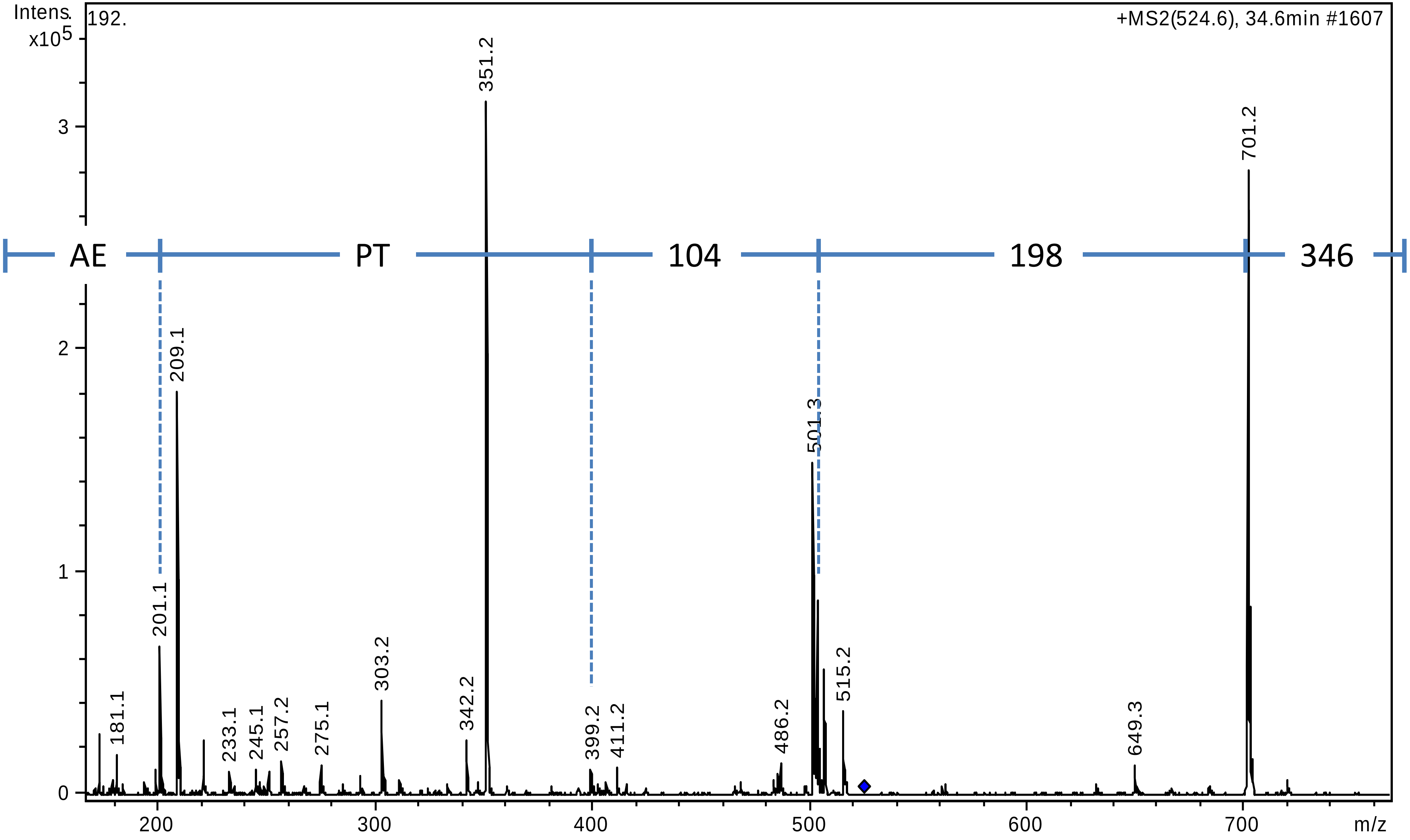

PG1030: ESFIT-Mod630

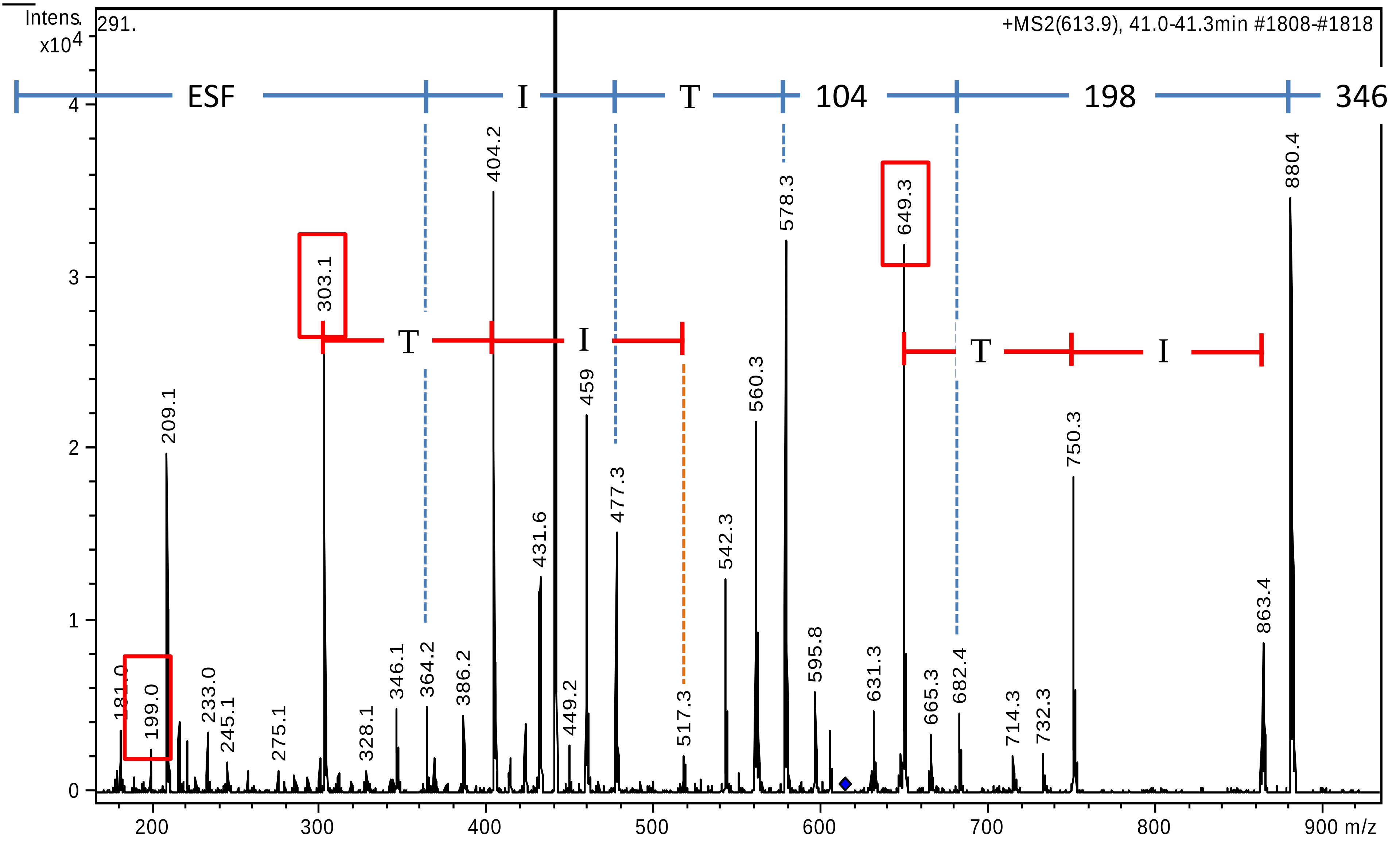

RgpA$_{A4}$: VCVDYIPD-Mod630

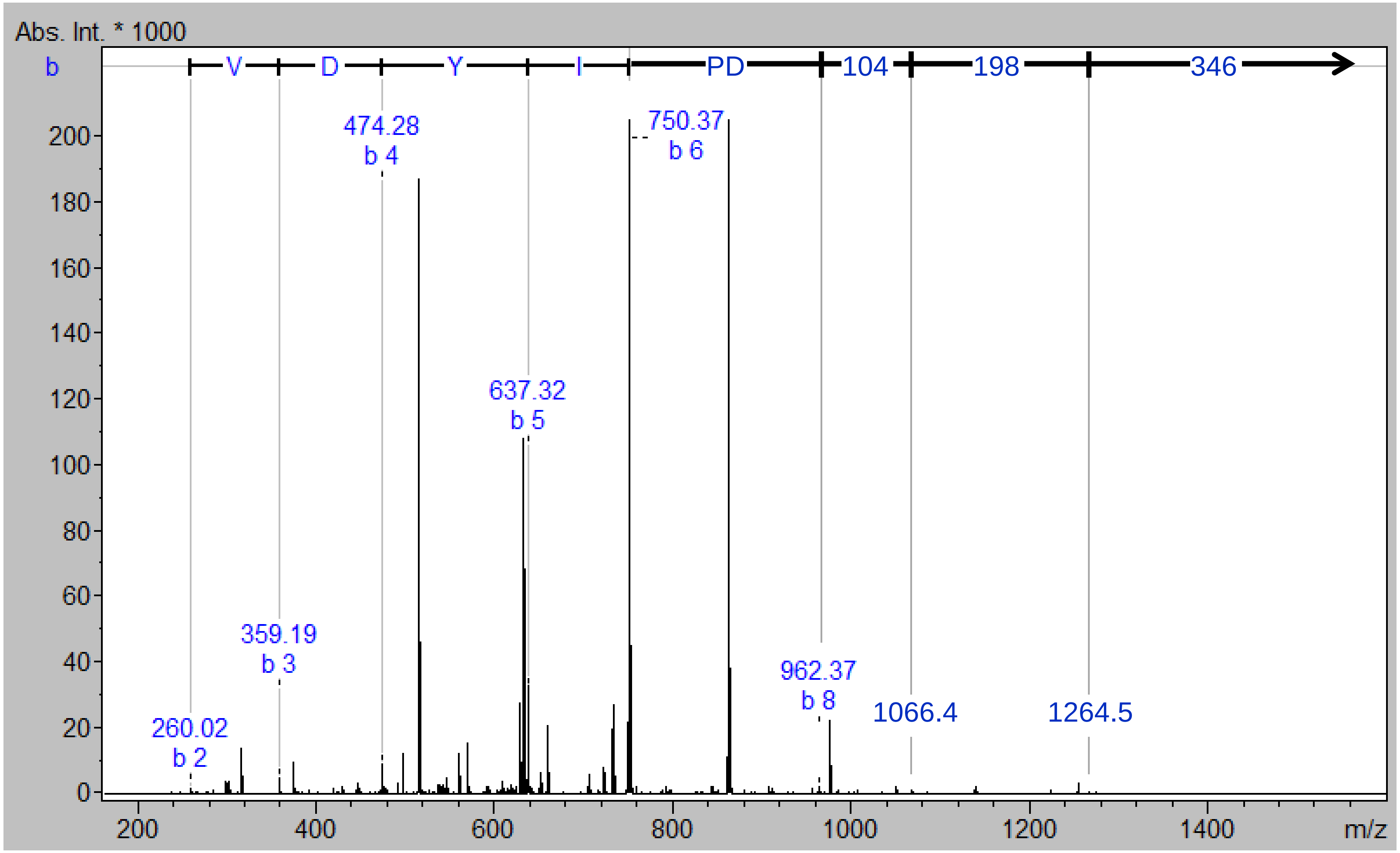

Kgp_{A5}: VVYPNGESAIETATLNIT-Mod630

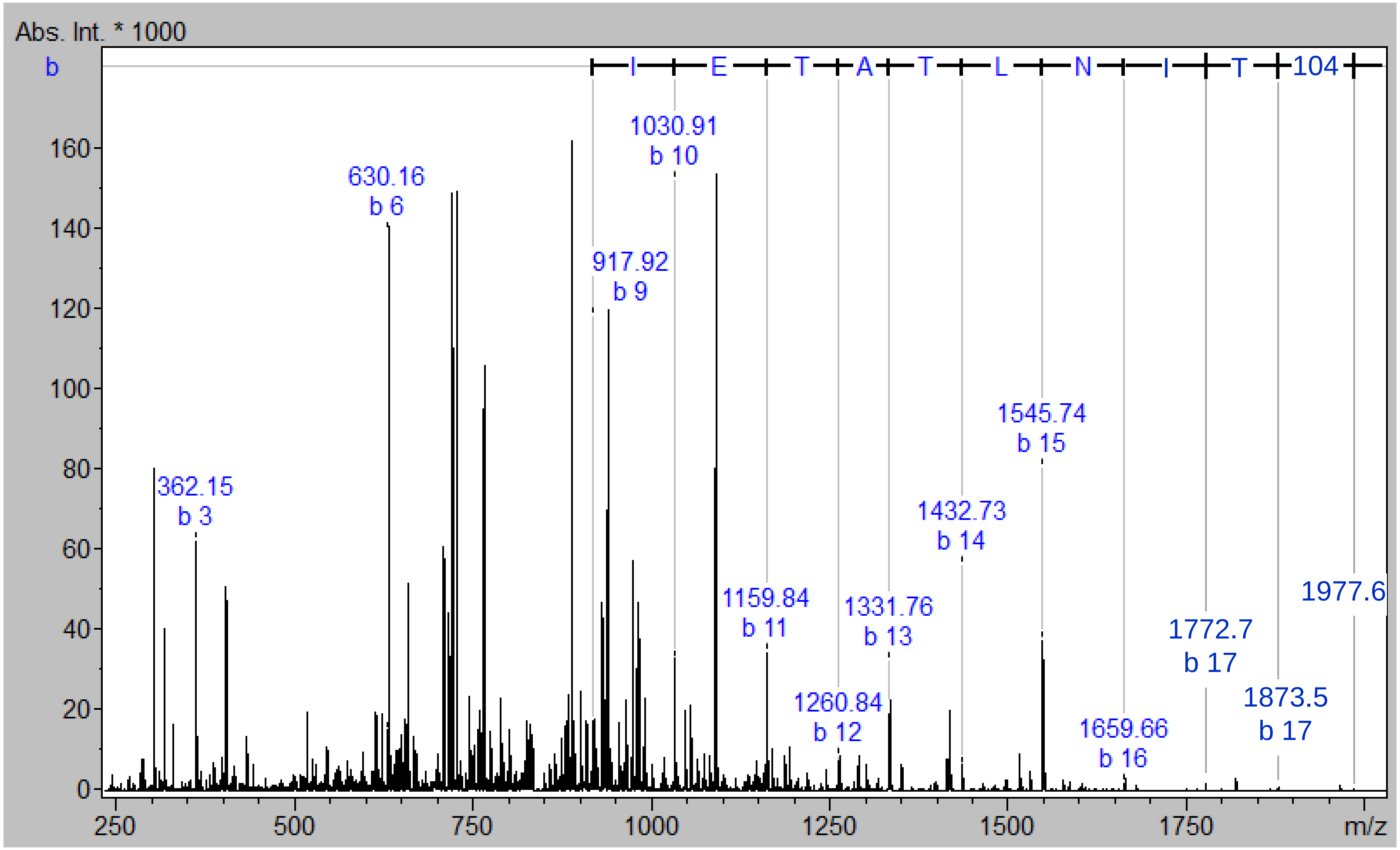

PAD: FTCMNET-Mod630

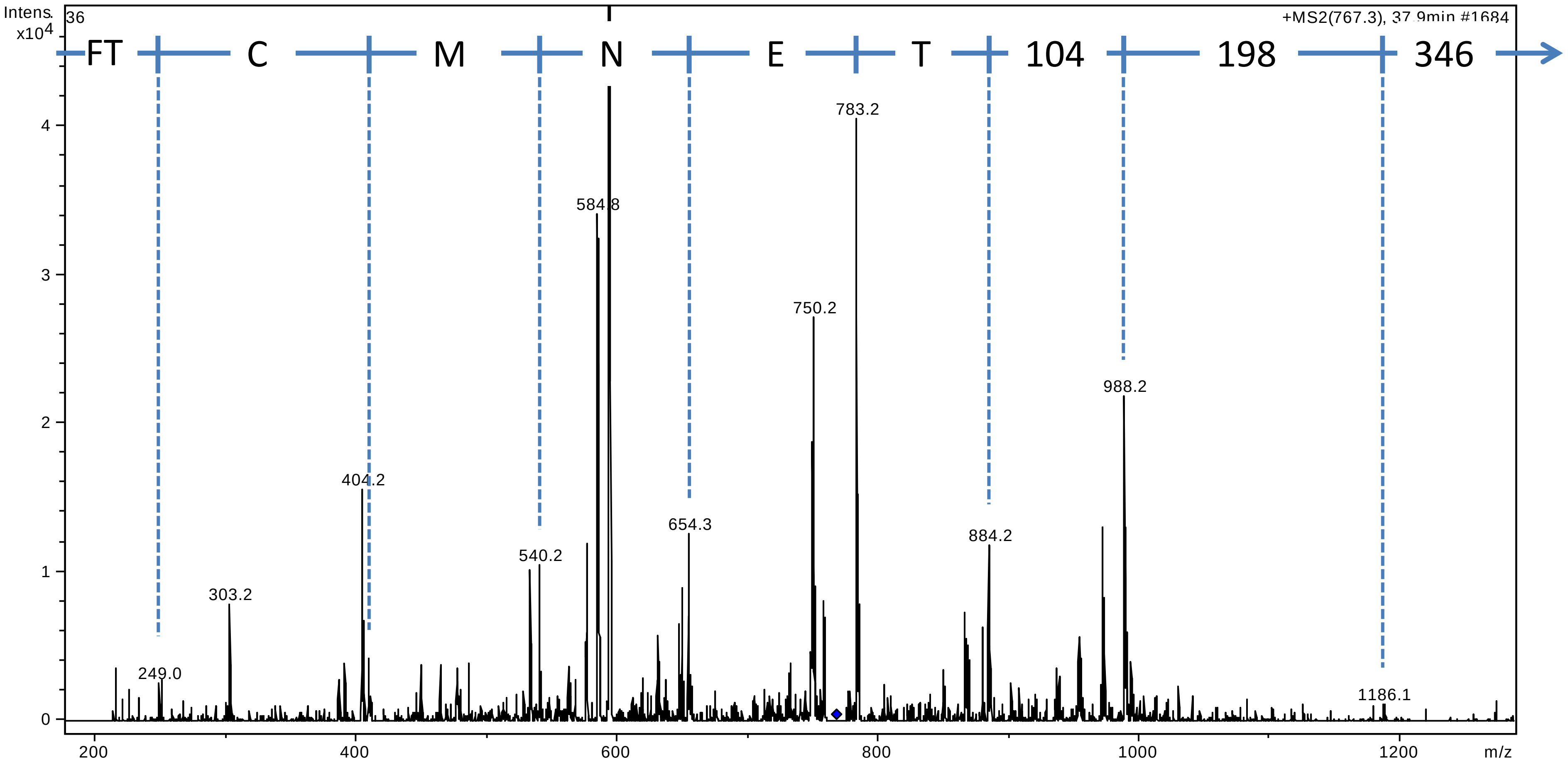

P27: KGE-Mod630

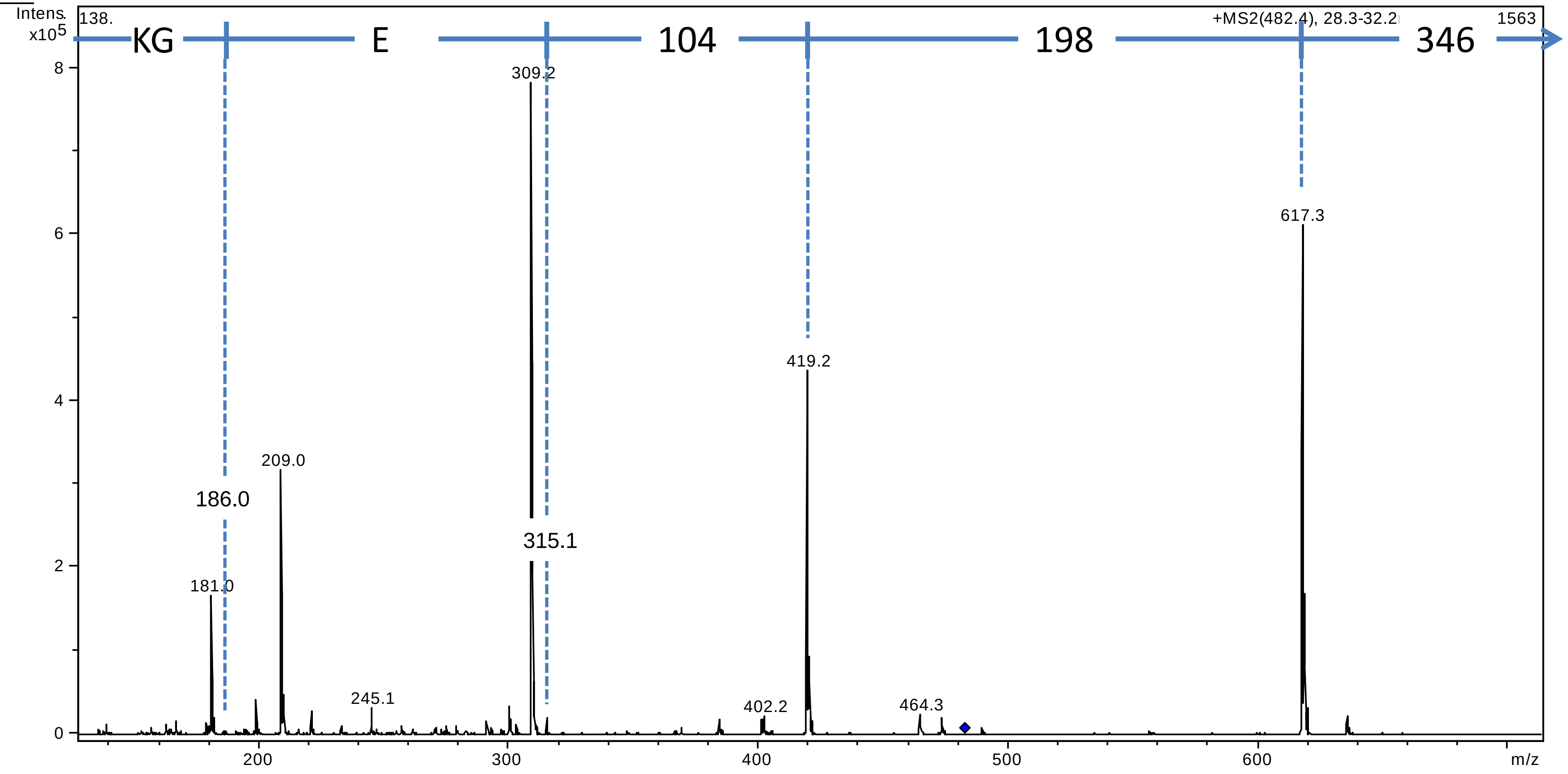

Orbitrap-P27: KGE-Mod630

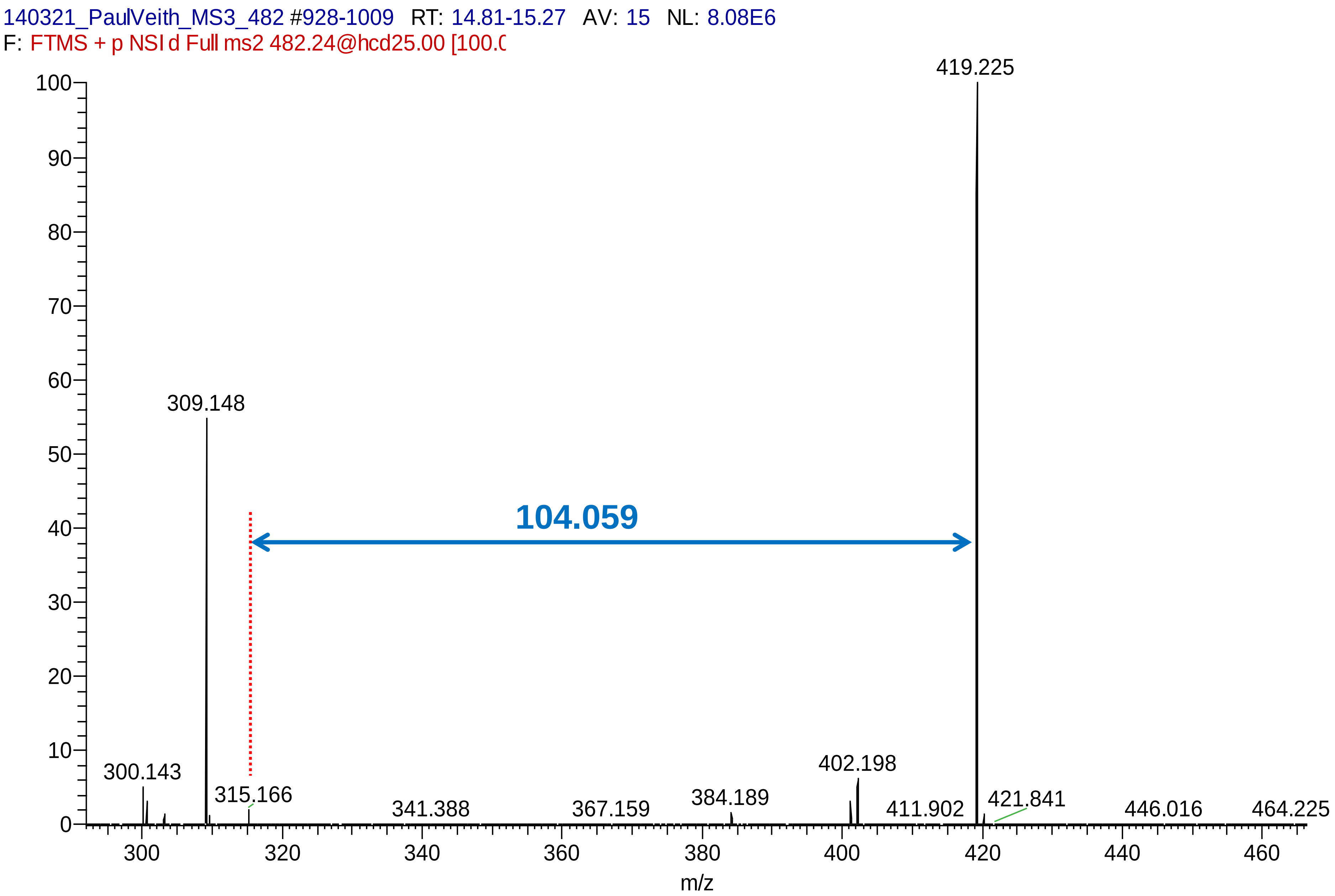

Orbitrap-PG1030: ESFIT-Mod630

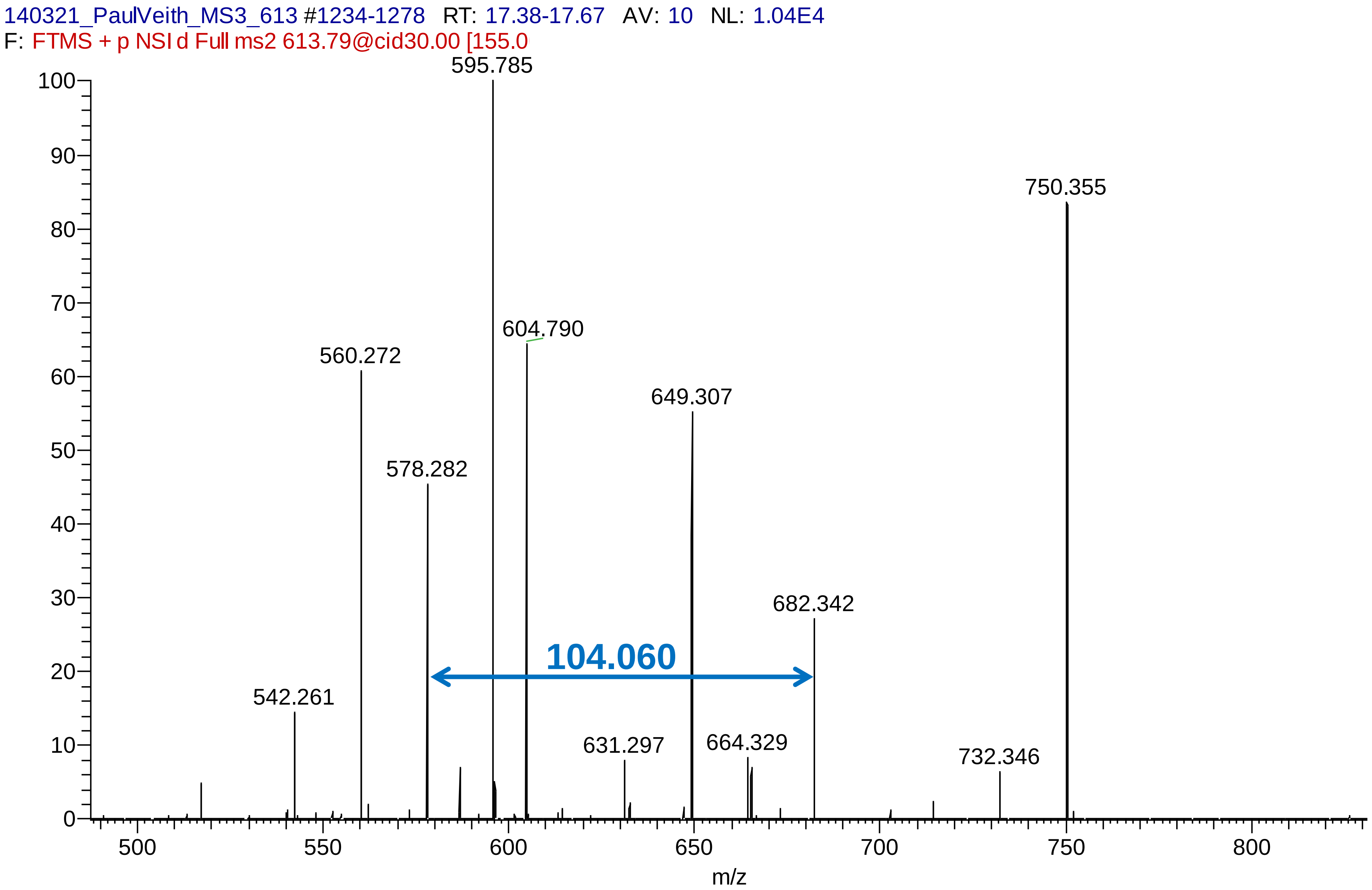

Supplement: S8 Fig — Modified CTD proteins were semi-purified, deglycosylated with TFMS, digested in-gel with trypsin and analysed by LC-MS/MS in positive ion mode using the ion trap MS and then also by Orbitrap MS. Each panel shows the MS/MS spectrum obtained for a C-terminal trytic peptide modified with the 648 Da linker. The proteins shown are HBP35, PG1030, RgpAA4, KgpA5, Peptidylarginine deiminase (PAD) and P27. The accurate mass of the 104 Da entity is shown for PG1030 and P27 Orbitrap MS/MS spectra. See also Fig 10. (PDF) [file ppat.1005152.s008.pdf]
