# Supplementary material for: Porphyromonas gingivalis Type IX Secretion Substrates Are Cleaved and Modified by a Sortase-Like Mechanism
Source: PLoS Pathog. 2015 Sep 4;11(9):e1005152. doi: 10.1371/journal.ppat.1005152 (PMC4560394; doi:10.1371/journal.ppat.1005152)
Supplement: S9 Fig — The sequence of PorU was obtained from the ATCC33277 strain (PGN_0022) since the N-terminal sequence of PG0026 from the W83 strain started in the wrong place [19]. 16 full length orthologs were randomly chosen to give a broad distribution across the Bacteroidetes phylum and aligned using Clustal W. The active site Cys and the conserved Arg residues are highlighted and marked with an arrow. (PDF) [file ppat.1005152.s009.pdf]

Supplementary Fig. 9

Multiple alignment of PorU (PGN_0022) (top line) with 16 randomly chosen full-length homologs from a diverse range of species within the Bacteroidetes phylum.

```
gi|188993886|ref|YP_001928138.     --------------------------------------------------
gi|229496468|ref|ZP_04390183.1     --------------------------------------------------
gi|262384061|ref|ZP_06077197.1     --------------------------------------------------
gi|150009174|ref|YP_001303917.     --------------------------------------------------
gi|255015781|ref|ZP_05287907.1     --------------------------------------------------
gi|154494061|ref|ZP_02033381.1     --------------------------------------------------
gi|218258224|ref|ZP_03474626.1     --------------------------------------------------
gi|224536225|ref|ZP_03676764.1     -------MFIKRCWLLCCFIAFLLPVSAQEFITLNWQELSSAQTLP-VVT 42
gi|189463715|ref|ZP_03012500.1     -------MFIKRCWLLCCFIALLLPVSAQEFITLNWQELSSAQTLP-VVT 42
gi|282881188|ref|ZP_06289875.1     -------------------------MAQAHFFNLTANDVKIDSVLP-YFS 24
gi|282877309|ref|ZP_06286140.1     ----------MIGFLSLLCLS---LSAQERFFNLTAADVKIDSRLP-HFS 36
gi|258648534|ref|ZP_05736003.1     ----------MRKFLIIVFCLNALLSAAQHFIDLPAAKYPLSAALAPHYT 40
gi|124008749|ref|ZP_01693438.1     MKYITSL------IFACLLLFQHPTQAQNF-------------------- 24
gi|110639610|ref|YP_679820.1|      MSFLKSIGIFLLGIFCVPVLVHAQQSSATFDIEWLGVNHTITLLNGTTIT 50
gi|163754141|ref|ZP_02161264.1     MKKTLTF------LIGLLFISASAQN-KHFDLTWEDSPKEFLKENSTTAL 43
gi|150025454|ref|YP_001296280.     MKKIIFT------IVLFAFQMLFSQESATIAISWADG-KYSVTENYAVTI 43
gi|254494946|ref|ZP_01052732.2     --------------------------------------------------

gi|188993886|ref|YP_001928138.     --------------------------------------------------
gi|229496468|ref|ZP_04390183.1     --------------------------------------------------
gi|262384061|ref|ZP_06077197.1     --------------------------------------------------
gi|150009174|ref|YP_001303917.     --------------------------------------------------
gi|255015781|ref|ZP_05287907.1     --------------------------------------------------
gi|154494061|ref|ZP_02033381.1     --------------------------------------------------
gi|218258224|ref|ZP_03474626.1     --------------------------------------------------
gi|224536225|ref|ZP_03676764.1     RELPLGKDFRSFTYQVEIEFPEYQKLNRSEVAALEMRLDSLRQLPNENVA 92
gi|189463715|ref|ZP_03012500.1     RELPLGNAFRSFTYQVEIEFPEYQKLSRSEVAALEMRLDSLRQLPNEDVA 92
gi|282881188|ref|ZP_06289875.1     HSFELNECYADSIYTVSILYPEFIDMTTADVARYQQLSGAP--------- 65
gi|282877309|ref|ZP_06286140.1     HSFALGDQYANSFYSVSILYPEFIDMTSADIARYRQLSDAP--------- 77
gi|258648534|ref|ZP_05736003.1     TQISVTDSVG-----IAITFPEYQPVTPKEQQVLKKYFKQ---------- 75
gi|124008749|ref|ZP_01693438.1     --------------------------------------------------
gi|110639610|ref|YP_679820.1|      YPVAKDAFVNLDGVLVL-----QKDVVGKVSGTVVIENVTFFAGDSSSKS 95
gi|163754141|ref|ZP_02161264.1     PAFNEEYYVNND-DGTISFIAQWDDDRYVNKNTIQITNVAYGTISNQNLG 92
gi|150025454|ref|YP_001296280.     PNFKSDSFIYDDVKKSIFFSKKFETTFAIDEESLNLSDVVFEAIAEEKLG 93
gi|254494946|ref|ZP_01052732.2     --------------------------------------------------

gi|188993886|ref|YP_001928138.     ----------MKR---ILPIVAFLS---------------LFLALALPAK 22
gi|229496468|ref|ZP_04390183.1     ----------MKKRNSLLSVVPFPTDYHHKLIRFALIIGSFFLLFLGMGE 40
gi|262384061|ref|ZP_06077197.1     --------------------------------MRRLIYIFIIGLLLCSYT 18
gi|150009174|ref|YP_001303917.     --------------------------------MRRLIYIFIIGLLLCSYT 18
gi|255015781|ref|ZP_05287907.1     --------------------------------MRRLIYIFIIGLLLCSYT 18
gi|154494061|ref|ZP_02033381.1     --------------------------------MKRILCVLLFVFGLLTSA 18
gi|218258224|ref|ZP_03474626.1     --------------------------------MKRILCALLFVFGLLASA 18
gi|224536225|ref|ZP_03676764.1     FREGLPASPQINSFIKVSAHRGFLSISFVPVVFREGSYQRLNSFKLSVNS 142
gi|189463715|ref|ZP_03012500.1     FRKGLPASPQINTFTKISAHHGFLSISFVPVVFREGSYQRLNSFKLSVKS 142
gi|282881188|ref|ZP_06289875.1     ----LPALPIPQHKIAVNRKRGLLQVGFSPLVYRNHRYQILVSFMLRIEA 111
gi|282877309|ref|ZP_06286140.1     ----LPALPPIRQRLALDRKKGSLEVDFCPLVFRDGKYQILVSFMLRLEA 123
gi|258648534|ref|ZP_05736003.1     ----LGDTPEVKTSFGKSRKEGLLDISIFPFIKRDKRYYRLVSFKLELDS 121
gi|124008749|ref|ZP_01693438.1     --------------------------------------------------
gi|110639610|ref|YP_679820.1|      VLPALNVPENRTVTYAVRHNDGVTTRLYIPILQKDGAGNWAQLSSVSVQY 145
gi|163754141|ref|ZP_02161264.1     GIDKRRLPTSIEAKIVNSSARGKNAHILIVSPLINDNGVIKKVLSFDVNY 142
gi|150025454|ref|YP_001296280.     DLDRSKIPERLNATIVNVNARDINYGLLTFSPIIKEGGTYKRVLSLSFGY 143
gi|254494946|ref|ZP_01052732.2     --------------------------------MIKKG-----FLLLSCMF 13

gi|188993886|ref|YP_001928138.     AQRAMG-------------------KTADRSLMASGHWVKIRVDASGVYR 53
gi|229496468|ref|ZP_04390183.1     YVFAQN-------------------KVDESSPLATGRWVKISVDKSGLYT 71
gi|262384061|ref|ZP_06077197.1     WADGS--------------------RYASKSLLSEGKWVKIRVDKTGIYK 48
gi|150009174|ref|YP_001303917.     WADGS--------------------RYASNSLLSEGKWVKIRVDKTGIYK 48
gi|255015781|ref|ZP_05287907.1     WADGS--------------------RYASKSLLSEGKWVKIRVDKTGIYK 48
gi|154494061|ref|ZP_02033381.1     WADSS--------------------RYASESVLNSGKWVKIQVAEDGIYK 48
gi|218258224|ref|ZP_03474626.1     WADGS--------------------RYASESVLSSGKWVKIQVAEDGIYK 48
gi|224536225|ref|ZP_03676764.1     FLKKDRIGEETATRNLKTTLSEVSLKDCTTSLLASGRWTKISVRNTGVFK 192
gi|189463715|ref|ZP_03012500.1     YPKTDGIGEGTATRGSGTTLTRASLKDCTTSLLASGRWTKIGVRNTGVFK 192
gi|282881188|ref|ZP_06289875.1     KPLMPS-----MKRATAKTRASSNSRYADHSVLASGKWAKIRVPSTGVYQ 156
gi|282877309|ref|ZP_06286140.1     QPRKQS-----LRHAAVQTRAGGASRYAAHSVLASGKWAKIRVPASGVYR 168
gi|258648534|ref|ZP_05736003.1     IALRTSQ-DKRLFNKQLNAASGAANRYAPHSVLATGKWVKIHVGGEGIYE 170
gi|124008749|ref|ZP_01693438.1     ---------------------------ASSSVLGTGTWYKIGTTQQGIYK 47
gi|110639610|ref|YP_679820.1|      SLTSNYDETPVRTDVYSRANANARTTNTTGSVLASGDWYKVAVSESGIYK 195
gi|163754141|ref|ZP_02161264.1     SFLARRPNN----------SIAKNTAAVTSSVLASGNFYRFSVQESGIHR 182
```

```
gi|150025454|ref|YP_001296280.      TAGASAVLYR--------QNITTATSQVESSVLKTGNWRKFYVEKSGVYA 185
gi|254494946|ref|ZP_01052732.2      LMQKS-------------------IAQVSSSVLASGDWYKFSVDTTGVFK 44
                                                                  * :  * : :. .   *:.

gi|188993886|ref|YP_001928138.      LTDEQLRANG----FSDPSKVGVFGYGGGVLPEDLS---RIT-TDDLPPV 95
gi|229496468|ref|ZP_04390183.1      LSYDKLRQMG----FSNPEAVGVYGRGGRLLSEDLQ---KASSVAGLPQV 114
gi|262384061|ref|ZP_06077197.1      LSYADLKNMG----FSDPSKVSVHGYGGWPLDEDF----SKEYIDDVPST 90
gi|150009174|ref|YP_001303917.      LSYADLKNMG----FSDPSKVSVHGYGGWPLDEDF----SKEYIDDVPST 90
gi|255015781|ref|ZP_05287907.1      LSYADLKNMG----FSDPSKVSVHGYGGWPLDEDF----SKEYIDDVPST 90
gi|154494061|ref|ZP_02033381.1      LTAADLKKMG----FSNLDKVAVYGYGGWPLDEDF----STTYIDDVPEV 90
gi|218258224|ref|ZP_03474626.1      LTATDLKKMG----FSNLDKVAVYGYGGWPLDEDF----STTYIDDVPEV 90
gi|224536225|ref|ZP_03676764.1      ITNAELKKMG----FSRPEKVRVFGYGGYLLSQRF----NEHPAADLPEV 234
gi|189463715|ref|ZP_03012500.1      ITNAELKKMG----FSRPEKVRVFGYGGYLLSQRF----NEHPAADLPEV 234
gi|282881188|ref|ZP_06289875.1      LTDELIRQAG----FTDLQKVKVYGYGGRLQNEQL-VSEELIATDDLKEV 201
gi|282877309|ref|ZP_06286140.1      LTDDLIRKAG----FTDINKVKVYGYGGRLQNEKL-VAEDLVATDDLKEV 213
gi|258648534|ref|ZP_05736003.1      LSATKLKEMG----FADINRVKVFGYGGRVLPAVFDFSSADRLMDDLEEV 216
gi|124008749|ref|ZP_01693438.1      IDYNFLKDAGVDVDNINPRNIQLYGNVGGMLPQSNSASRVDDLQENSIWV 97
gi|110639610|ref|YP_679820.1|       ITPSYLSSIGINTSSLDPSMIQLYGNGGGMLPQANSDARADDLVENAVFI 245
gi|163754141|ref|ZP_02161264.1      ISADFLNQIGLNTGNINPRKIKIYGNGGRMIPHVNSENEYYDVTENAISV 232
gi|150025454|ref|YP_001296280.      ISKAFLSGLGFNVN-VDPRTIKIYGNGGRMIPLLNSVPYPTDLAENAIQF 234
gi|254494946|ref|ZP_01052732.2      IDRNLLQQIGISTNNLDPKKIHIYGNGGQLLPVLNSDFRYDDLQENAIYI 94
                                    :    :   *          : :.*  *                 .

gi|188993886|ref|YP_001928138.      P-----VLRQGNALYFYAVGPVTWFYNPAKTT-MEHTVNTYSTHGYYFLS 139
gi|229496468|ref|ZP_04390183.1      P-----LLRHNNAIYFYGEGTTHWYYDTAQKC-YRHVTNHYTRLGYYLLS 158
gi|262384061|ref|ZP_06077197.1      P-----VWRGSDYLLFYGKGPVKWEYDSSSQT-FVHTNNPYSLYGYYFVT 134
gi|150009174|ref|YP_001303917.      P-----VWRGSDYLLFYGKGSVKWEYDSSSQT-FVHTNNPYSLYGYYFVT 134
gi|255015781|ref|ZP_05287907.1      P-----VWRGSDYLLFYGKGPVKWEYDSSSQT-FVHTNNPYSLYGYYFVT 134
gi|154494061|ref|ZP_02033381.1      A-----VWRSADYLLFYGKGPRKWEYSSSDKS-FIHTNNPYSNYGYYFVT 134
gi|218258224|ref|ZP_03474626.1      A-----VWRNADYLLFYGKGPRKWEYSSSDKS-FIHTNNPYSNYGYYFVT 134
gi|224536225|ref|ZP_03676764.1      P-----LLRLSDGVLFYGRGTVSWTPDSQNTY-FVRERNFYSDEGYYFLT 278
gi|189463715|ref|ZP_03012500.1      P-----LLRLSDGVLFYGRGTVSWKLSSDNTY-FVRERNFYSDEGYYFLT 278
gi|282881188|ref|ZP_06289875.1      P-----TCWVDGRRLFYAYGTVS--WDSNTAT-RRTRN-PYSDTGYYFLT 242
gi|282877309|ref|ZP_06286140.1      P-----TCWVDGRRLFYAYGPVS--WDSNTAT-RRTRN-PYSDYGYYFLT 254
gi|258648534|ref|ZP_05736003.1      P-----LYRRNGSVLFYAEGTVRKMWSPTRLK-WTHKNNTYARYAYYFVT 260
gi|124008749|ref|ZP_01693438.1      AGQDDGNFDANDYILFYAQGAVEWTYN-ATNKLFEHTQNIYSDSVFCFLT 146
gi|110639610|ref|YP_679820.1|       SEDGNGVFDNDDYILFYAESPHTWNYNPAGGTTFTHTLNLYSDQNFYFLT 295
gi|163754141|ref|ZP_02161264.1      VGEEDGEFNANDYILFYAEGSRKWNKDSQ------THINAYTDKTYYYIT 276
gi|150025454|ref|YP_001296280.      VGENDGVFNEGDFILFYAEGVDNYNNDSD------THVNLYADKSYYYVT 278
gi|254494946|ref|ZP_01052732.2      DGEQDGSFDTNDFILFYAKGPHDWEVDLANET-ARHRQNIYSDKAYYFIT 143
                                               .   **. .      .             *:   :  ::

gi|188993886|ref|YP_001928138.      DAAG-APLQMSQYTG-GGASAEALIDYYDELMLHEQELYSPKE--SGRDL 185
gi|229496468|ref|ZP_04390183.1      DAATPGPLLMEERKPTASQAAPALTSTYDALVLHERDIFSPKQ--SGRML 206
gi|262384061|ref|ZP_06077197.1      DATP-TN-DMTSVAQASG--ASTRITTYDDYLLHEQELVSVNQ--SGREF 178
gi|150009174|ref|YP_001303917.      DATP-TN-DMTSVAQASG--ASTRITTYDDYLLHEQELVSVNQ--SGREF 178
gi|255015781|ref|ZP_05287907.1      DATP-TN-DMTSVAQASG--ATNRITTYDDYLVHEQELVSLAK--SGREL 178
gi|154494061|ref|ZP_02033381.1      EKET-AGRTMEKAASAAG--ATLQVTTFDDYVLHEEELVSVNS--SGREL 179
gi|218258224|ref|ZP_03474626.1      EKET-AGRTMEKAVSADG--ATLQVTTFDDYVLHEEELVSVNS--SGREL 179
gi|224536225|ref|ZP_03676764.1      DRED-IPEMETEIFSSLKETSANRLTTFNSFAIYEKDAYSWAG--TGRQL 325
gi|189463715|ref|ZP_03012500.1      DRED-IPEMEVEVLSSLKEPSANRLTTFNSYALHEKEVYSWAS--TGRQL 325
gi|282881188|ref|ZP_06289875.1      QSED-TPVVLDSTAFVHS--FYPSPDY--YHTLYEVDGYSWYH--GGRNL 285
gi|282877309|ref|ZP_06286140.1      QTDD-ALTSVDSTAFLAS--FYPSPDD--YHTLYEVDAHAWYA--GGRNL 297
gi|258648534|ref|ZP_05736003.1      EGE--QPLALNRIAATQT--PDTTLDATISQVVLDDDAFCWYE--GGTEM 304
gi|124008749|ref|ZP_01693438.1      IGSN-EGKRVQSQENLSG--TTQTLTTYDDYQVYEKDERTILEQGSGREW 193
gi|110639610|ref|YP_679820.1|       VNQQ-PGKRISSQGSLGG--AAQTVSSFDEHFFTETDQYNIMD--SGREW 340
gi|163754141|ref|ZP_02161264.1      ADGSDDGKRIPEAVQPTAT-PDITINQYDDYQFHEEDNVNIVK--LGRRW 323
gi|150025454|ref|YP_001296280.      SSAS-IGKRITPILESPQP-PDAIFTTYDEYQYHEIDKTNLAH--LGRRW 324
gi|254494946|ref|ZP_01052732.2      VNET-DGKRIQAETPISSV-ATNAFTTFDDYTFFEREERSILA--VGTQW 189
                                                                      : :         *

gi|188993886|ref|YP_001928138.      YGESFSAVNTRTVKFPLRGNTRSSGE-LGTVFSYIAKARSAGGGREMSLS 234
gi|229496468|ref|ZP_04390183.1      FGEPLLGGQPKRIIVNLGHGEQAGNA-IKINYAYMARPSTQG---FFTLS 252
gi|262384061|ref|ZP_06077197.1      FGEDFSGARPRTISTFSSIPGITDAD-GKVTMRFISRINSGSGTASLSIN 227
gi|150009174|ref|YP_001303917.      FGEDFSGARPRTISTFSSIPGITDAD-GKVTMRFISRINSGSGTASLSIN 227
gi|255015781|ref|ZP_05287907.1      YGEDFSGATPRTLSTFSSIPGITNED-AKVTMSFVSKVTSGTGVASLSIN 227
gi|154494061|ref|ZP_02033381.1      YGESFTSTLSRDFT--ISVPGITNDE-GKATLSFIS---RGNGTITMNVD 223
gi|218258224|ref|ZP_03474626.1      YGESFTSTLSRDFT--ISVPGITDDE-GKATLSFIS---RGNGTITMNVD 223
gi|224536225|ref|ZP_03676764.1      Y-EDYDYVAGNTKSYTLNLPGIVPEAAGWLTTVFAARSIDASTSYSVSVN 374
gi|189463715|ref|ZP_03012500.1      Y-EDYDYATGNTKNYTLSLPGIVPEDSVWLTTVFAARSIGASTYYSVAVN 374
gi|282881188|ref|ZP_06289875.1      F-HPKAIDVAESATYTIPSLASKGKAKMAVNVSAGSAS-----RISVMHN 329
gi|282877309|ref|ZP_06286140.1      F-DPNPIPAGSSKTFIIPSKADQGSAKMSVNLSAGTAS-----LATVTYQ 341
gi|258648534|ref|ZP_05736003.1      Y-DSYDFANGATHAYKLNTPFYNGKRNAEVEIAFGAAAQKKALQVNVQLN 353
gi|124008749|ref|ZP_01693438.1      YGEVF-DFETNQSFTFDTPGLLANAPLTLTSFVMGRSALDTKFSVSLNGV 242
gi|110639610|ref|YP_679820.1|       YGIQFTQYVPSQNFSFNITGILPNADLKLKSAVLSRSYVSTRFVYKVNNI 390
gi|163754141|ref|ZP_02161264.1      FG-EVFNIETEQTFDFTIPNLITTEPVTIGIKPAAISNGTTTMTTAINGQ 372
```

```
gi|150025454|ref|YP_001296280.      FG-EPFNINNNQNFDFSFPNIVTTNPVSVKVQVGAVSTNNSSFSIKVNNL 373
gi|254494946|ref|ZP_01052732.2      FFNEDFNIENTQSFTIPFTNAVSGENITVRTRGVSNSVVASTMDVVVNGQ 239
                                    :                                             .    

gi|188993886|ref|YP_001928138.      ANGILIFSDPFSMT---SNEVSNSYLAGKKRRLYRSTP------------ 269
gi|229496468|ref|ZP_04390183.1      LDGRELAKDPISLS---EDHTTRDFLAG-IYHFRPNVT------------ 286
gi|262384061|ref|ZP_06077197.1      DSELLDITIPSIQT---VSSNVRSYTKAIPGTTTALWK------------ 262
gi|150009174|ref|YP_001303917.      DSELLDITIPSIQT---VSSNVRSYTKAIPGTTTALWK------------ 262
gi|255015781|ref|ZP_05287907.1      QSNVLDVSIR---------SNREEYVKALEGVSTAVWK------------ 256
gi|154494061|ref|ZP_02033381.1      GNALISGSVS---------VPSDEYEVARELYRERAWT------------ 252
gi|218258224|ref|ZP_03474626.1      GNSVISGSVS---------VPSDEYEVARELYRERAWT------------ 252
gi|224536225|ref|ZP_03676764.1      GEPKGNITLASID------SDNQYYTRATSATINASWQ------------ 406
gi|189463715|ref|ZP_03012500.1      GKVRGNATLASIS------SDNQYYTRATSASISTSWL------------ 406
gi|282881188|ref|ZP_06289875.1      GKQLGELAIA--------------LSKNDKGNETSGVY------------ 353
gi|282877309|ref|ZP_06286140.1      NAELGTLNIR--------------LREHDSGNEAGAVY------------ 365
gi|258648534|ref|ZP_05736003.1      NSDLGTFSISR------------YYGETESARETRSKY------------ 379
gi|124008749|ref|ZP_01693438.1      T--LGTQSIA-------AQPTGTYDVKGINNVRDFTLN-------TSQLS 276
gi|110639610|ref|YP_679820.1|       ANLIPDQYLP-------ILTTEEHADLGRDSFIIDTLN-------AASLG 426
gi|163754141|ref|ZP_02161264.1      P----AQDINGNSAFNFSFSPINTSTLATQHVVSGATQLRQGLIVDQVNL 418
gi|150025454|ref|YP_001296280.      N----VGNIN--------LFGVSSYALGSEGAFS-----------NTINT 400
gi|254494946|ref|ZP_01052732.2      D----AYSIN--------YPATNPSSLTKAFALERS---------ASLNN 268


gi|188993886|ref|YP_001928138.      MNSLVNELRLDANYSMTGDAVNLDFIEVATQNDLRYD-GAPMHIRRFSNL 318
gi|229496468|ref|ZP_04390183.1      VTSSSRNLALDASYRPAGDNAYVDFLEFIIEQRLHYTTGQQTDFRRIQ-- 334
gi|262384061|ref|ZP_06077197.1      GSKSEKNNVVVSYSSSGHTNVRLDYIRMQFVRTLRPYGASTFFRSLTS-- 310
gi|150009174|ref|YP_001303917.      GSKSEKNNVVVSYSSSGHTNVRLDYIRMQFVRTLRPYGACTFFRSLTS-- 310
gi|255015781|ref|ZP_05287907.1      GEKSEKNSVVVSYNSASHNNVRLNYVRLHFKRTLQPYGAYTFFRSIAS-- 304
gi|154494061|ref|ZP_02033381.1      ADKGETVKVNIGYSTTGHKNVHLNYFRLQMKRQLKVYDNYTFFRSLSA-- 300
gi|218258224|ref|ZP_03474626.1      ADKGETVKVNIGYSTTGHKNVHLNYFRLQMKRQLKVYDPYTFFRSLSA-- 300
gi|224536225|ref|ZP_03676764.1      GTKSENTVVTITHTRPSGTSGRLDYIALNYMRALTLNTPYLTFRSLAS-- 454
gi|189463715|ref|ZP_03012500.1      GTESENTIVTVIHTRPSGTSGRLDYIALNYIRSLTLNAPYLTFRSLAS-- 454
gi|282881188|ref|ZP_06289875.1      QIERTSMTDTVKLTTRSGSPSRLDYISFIWQEPLPAP------------- 390
gi|282877309|ref|ZP_06286140.1      EINRTGTTDSVKISTNSGGPIRLDYISFTWQKPLPAP------------- 402
gi|258648534|ref|ZP_05736003.1      SVANLKEENTFNFSVGGNSSARLNYISISYARRLDATDQAYAFLPATI-- 427
gi|124008749|ref|ZP_01693438.1      GLSQLTIDLLYD-KNGGSSSGYLNYLRVNYQRQLQLYNNQTTFRAIAS-- 323
gi|110639610|ref|YP_679820.1|       SNTSLSIGMTLT-GGIATSMAHLNYLEVVFEKSLRLYGNYTSFRSSKS-- 473
gi|163754141|ref|ZP_02161264.1      SSENLSITLTYQNNGVAGSVGYLDYITVEARRMLTGIGSQFTFRYNDA-- 466
gi|150025454|ref|YP_001296280.      TSSNISVNLNYNNNGVPSGIGYLDYITLKAKSNLKGYGKQFRFSVDAV-- 448
gi|254494946|ref|ZP_01052732.2      TSENITVSITYNSNGNPSANAFLDYIEVLGTKNLSYSDFQFSFRNFDQ-- 316
                                                          :::. .     *                

gi|188993886|ref|YP_001928138.      PVLGGESCRFVISEVPESLVVLQANSSLTASLVPVKTVGDKTIEFVAPPK 368
gi|229496468|ref|ZP_04390183.1      --EGSDALLYQVSGLPQNGIILATSPESSLPYRVQTTQSGGTHSFTTQAL 382
gi|262384061|ref|ZP_06077197.1      ---VGNASRFVISEANSNTLVFDVTDALN-VKRVEADLNGSELSFTIPAG 356
gi|150009174|ref|YP_001303917.      ---VGNASRFVISEANSNTLVFDVTDALN-VKRVEADLNGSELSFTIPAG 356
gi|255015781|ref|ZP_05287907.1      ---IGNVSRFVVQGANANTLVFDVTDAQR-VRLMETELNGSELTFSIPAG 350
gi|154494061|ref|ZP_02033381.1      ---RGNASRFVIQGADASTLVFDVTDGVN-PQQMETSLNGTELSFSIPAS 346
gi|218258224|ref|ZP_03474626.1      ---RGNASRFVIQGADAGTLVFDVTDGVN-PQQMETSLNGTELSFSIPAS 346
gi|224536225|ref|ZP_03676764.1      ---IHKETTFVLSGATASTVVWDVTNPAN-IGRIEGSFADGTYTFTIPAG 500
gi|189463715|ref|ZP_03012500.1      ---INKETTFVLSGATASTVVWDVTDPAN-ICRMEGSFVGGTYTFTIPAG 500
gi|282881188|ref|ZP_06289875.1      ------------------------------------HLKG---------- 394
gi|282877309|ref|ZP_06286140.1      ------------------------------------QLKG---------- 406
gi|258648534|ref|ZP_05736003.1      ---MQEPVRLHIANATQTTQVWQIGRAKEPLAQVAGTLKGNTLEVDIDDS 474
gi|124008749|ref|ZP_01693438.1      -LEQP-LTAFSVANVGNGAIVWDISNPLH---PVNQPYTLSGSNAVFGAS 368
gi|110639610|ref|YP_679820.1|       -LLQP-TTQYSISNASGTENVWDVSNPLN---VVNQAYDFTGSAIEFGSS 518
gi|163754141|ref|ZP_02161264.1      -RNILGVGEFVISNAASIEQVWDVTDIWN---VTKITNDALANNFSFKAP 512
gi|150025454|ref|YP_001296280.      -SSSIGICEYQISNAASIGQVWDITDIYN---VSTKFNTGQAQ-FSLKTF 493
gi|254494946|ref|ZP_01052732.2      -ATASGTISYEITNGANAFQVWDVSNSIT---PKRIQNTSSGNNFTFNAN 362


gi|188993886|ref|YP_001928138.      GQDRRTINTFYAVDLSQASAPEILGAVPNQNLHGEEIPD-------LIIV 411
gi|229496468|ref|ZP_04390183.1      DREGQPYN-FLACSWQDAYTPNVVGEVANQNIHSAPIPD-------LIIL 424
gi|262384061|ref|ZP_06077197.1      R---LREFVLVQTNQT-FPSPEVVGEVASSNLHGLEQRD-------MIII 395
gi|150009174|ref|YP_001303917.      R---LREFVLVQTNQT-FPSPEVVGEVASSNLHGLEQRD-------MIII 395
gi|255015781|ref|ZP_05287907.1      T---LREFVLVRTDQS-FISPEVVGEVENQNLHALEQKD-------MIII 389
gi|154494061|ref|ZP_02033381.1      AS--LREFVVVKPSQ--IKAPVTVGEVVNQNLHALPQQD-------MIII 385
gi|218258224|ref|ZP_03474626.1      TS--LREFVAVKPSQ--IKAPVTVGEVTNQNLHALPQQD-------MIII 385
gi|224536225|ref|ZP_03676764.1      E---LREFVAITPEAGGFDTVENVGGVANQNLHSLEATD-------MIII 540
gi|189463715|ref|ZP_03012500.1      K---LREFVAITPEASGFDTVETVGNIANQNLHSLGATD-------MIII 540
gi|282881188|ref|ZP_06289875.1      ---------------VNFPVPEFVQAVTNQDLHAHGFAD-------MVII 422
gi|282877309|ref|ZP_06286140.1      ---------------TSFPVPEYVYNITNQDLHGDGFVD-------MVII 434
gi|258648534|ref|ZP_05736003.1      N-----RRYIIVDVAKAYPAPSVDGQIENQDLHADGAVD-------MVII 512
gi|124008749|ref|ZP_01693438.1      TATALKQFVVFQGSDFPTPESVQT--LPNQNLHALLP---------TNLV 407
gi|110639610|ref|YP_679820.1|       -STSLKEFIIFSGNNFSAPTFIER--VSNQNLHDITSGN------IPDMI 559
gi|163754141|ref|ZP_02161264.1      -MGEVREYIALDTSQFLIPSIEPNSLLVNQDLKGSVFSQ-----GDVEYI 556
```

```
gi|150025454|ref|YP_001296280.      -MGEAKKYIAIDNVDFYTPLKDLNTNVFNQDLKGTIFFTKQGVFEDIDYL 542
gi|254494946|ref|ZP_01052732.2      -AGELKEYVVLDANDFYTPETVENARVTNQDLHG---------LKDINYL 402
                                                              : ..:::                :

gi|188993886|ref|YP_001928138.      STQ--ALLPEADRLATYRREKNGLKVLVVLQEQVFNEFSGGTPDATAYRL 459
gi|229496468|ref|ZP_04390183.1      SPE--AFLSEAERLAEFHRSVDGMQVLVVGETALFNEFNAGTPDATAYRL 472
gi|262384061|ref|ZP_06077197.1      SAP--SLVQQAERLAVAHREKDGLTVEVVTPEAIYNEFSSGTPDATAYRR 443
gi|150009174|ref|YP_001303917.      SAP--SLVQQAERLAVAHREKDGLTVEVVTPEAIYNEFSSGTPDATAYRR 443
gi|255015781|ref|ZP_05287907.1      AAK--AFQTQAERLAEEHRATDGLTVEVVDPQSIYNEFSSGAPDATAYRR 437
gi|154494061|ref|ZP_02033381.1      AQP--NFTTQAERLAEAHRTKDNLTVRVVTPESIYNEFSSGTPDATAYRR 433
gi|218258224|ref|ZP_03474626.1      AQP--NFTTQAERLAEAHRTKDNLTVRVVTPESIYNEFSSGTPDATAYRR 433
gi|224536225|ref|ZP_03676764.1      APDRKDLLAQAERLAQAHREKDGLSVLVLTAPQIYNEFSSGTPDGTAYRR 590
gi|189463715|ref|ZP_03012500.1      SPDRKDLMTQAERLAQAHREKDGLSVLVLAAPQIYNEFSSGTPDGTAYRR 590
gi|282881188|ref|ZP_06289875.1      IPSSQKLKTQAERLKAFHEKHDGLRVQIVSANQLYNEFSSGTPDANAYRR 472
gi|282877309|ref|ZP_06286140.1      IPTSQKLHTQAERLKAFHEQNDGMRVRIVPADELYNEFSSGTPDANAYRR 484
gi|258648534|ref|ZP_05736003.1      IPQSGKLLAQAERLAEAHKKQ-GLRVKVVRADQLYNEYSSGTPDVAAYRR 561
gi|124008749|ref|ZP_01693438.1      IIVPEVFLSEAERLAAFRRTNDGLSVTVVKLSQVYNEFSSGKQDVTALRD 457
gi|110639610|ref|YP_679820.1|       IVTHPDFQSQAQRLANFRTTNDGLRVYVCTTEQIYNEFGSGKKDLTAIRD 609
gi|163754141|ref|ZP_02161264.1      IITPDAFVSQAERLADFHRNNSGMIVKVVTLASIYNEFNTGNPDIGAIRN 606
gi|150025454|ref|YP_001296280.      IITPASLSSQAERLANFHRINSNLNVKVINLENLYEEFSSGKQDIGAIRN 592
gi|254494946|ref|ZP_01052732.2      VITNSDLANEAQRLVDYHQTNSNLVGKVVLLDEIYNEFSSGSKDITGIRD 452
                                          :  :*:**   :    .:   :     :::*:. *  *  . *

gi|188993886|ref|YP_001928138.      FAKMFYDRWKANAPVGETFPMQMLLFGDGAHDNRKVSVAWQKPYLQQTEF 509
gi|229496468|ref|ZP_04390183.1      MAKYFYDRWMAAHPGEKQSAQQLLLFGDGAADNRKVSVHWESIGLQNTPF 522
gi|262384061|ref|ZP_06077197.1      LMKMFYDRSSSLG----NPPKYLLLFGDGIYDNRGISGEVQGVSRSN--M 487
gi|150009174|ref|YP_001303917.      LMKMFYDRSSSLG----NPPKYLLLFGDGIYDNRGISGEVQGVSRSN--M 487
gi|255015781|ref|ZP_05287907.1      FMKMFYDRSVSAG----NAPKYLLLFGDGIYDNRGICSDVKNIARDN--M 481
gi|154494061|ref|ZP_02033381.1      FMKMFYDRQTSEA----DAPKYLLLFGDGSFDNRKLTSAWKSVDMSN--M 477
gi|218258224|ref|ZP_03474626.1      FMKMFYDRQTSEA----DAPKYLLLFGDGSFDNRKLTSAWKSVDMSN--M 477
gi|224536225|ref|ZP_03676764.1      LMKMLYDRFPNEA----ERPKYLLFFGDCSYDNRMLTSSWKSYRPEN--F 634
gi|189463715|ref|ZP_03012500.1      LMKMLYDRFPNKA----ESPKYLLFFGDCSYDNRMLTSSWKNYRPEN--F 634
gi|282881188|ref|ZP_06289875.1      YLKMLYDRATTAA----DMPKYLLLFGDCVWDNRMLTAQCQSLHPDD--Y 516
gi|282877309|ref|ZP_06286140.1      YLKMLYDRAQTDA----DMPKYLLLFGDCVWDNRMLTANCRNLNPDD--Y 528
gi|258648534|ref|ZP_05736003.1      YMKMLYDRAQSNA----DMPRYLLLFGDCFWDNRMLTT--TKLNAAD--F 603
gi|124008749|ref|ZP_01693438.1      FARMLYLRDSQ-------VFKYLLLFGDASFDYKDRVANNTNFVPIYE-- 498
gi|110639610|ref|YP_679820.1|       FVKMVYDRGSAGS-----QLKYLLLFGKPTYDYKNHTGIGGNFVPTYE-- 652
gi|163754141|ref|ZP_02161264.1      FIKYVYDNASTVDN----RVKYVCMFGDASYDYKDRIEGNTNIVPVYQ-- 650
gi|150025454|ref|YP_001296280.      FVKYVYDNASIPAR----RLKYLCLFGDASYDFKDRIKNNTNIVPVFQ-- 636
gi|254494946|ref|ZP_01052732.2      FIKHLYTTNSSPST----QLKYVCFFGDSSYDYKDRIAANNNIVPVKL-- 496
                                      : .*                : :**.   * :

gi|188993886|ref|YP_001928138.      LLTFQA-------VNSTNVNS---------YVTDDYFGLLDDQPA---SV 540
gi|229496468|ref|ZP_04390183.1      LLTYQS-------SNSLNIYS---------YTTDDYFGYLRDGED---HL 553
gi|262384061|ref|ZP_06077197.1      LLTFQS-------QESLNVYS---------YATDDYFAFLEDNSG---SN 518
gi|150009174|ref|YP_001303917.      LLTFQS-------QESLNVYS---------YATDDYFAFLEDNSG---SN 518
gi|255015781|ref|ZP_05287907.1      LLTYQS-------RASLFEYSGLFYNSNFSYVTDDYYGLLDNVSE---EA 521
gi|154494061|ref|ZP_02033381.1      LLTYQT-------ENSLSSQS---------YVIDDYFGFLDDADN---KK 508
gi|218258224|ref|ZP_03474626.1      LLTYQT-------ENSLSSQS---------YVIDDYFGFLDDADN---KK 508
gi|224536225|ref|ZP_03676764.1      LLSYQS-------EASLEETS--------SYVTDDYFGFLDDEEG---DD 666
gi|189463715|ref|ZP_03012500.1      LLSYQS-------ETSLEETS--------SYVTDDYFGFLDDGEG---EN 666
gi|282881188|ref|ZP_06289875.1      LLCFES-------ENSFNEVD--------CFVDDGFFCALDDGEG---LN 548
gi|282877309|ref|ZP_06286140.1      LLCFES-------ENSFNALH--------CYVDDGFFCALDDGEG---LN 560
gi|258648534|ref|ZP_05736003.1      LLAYEAGPGDGQTDISIGTLY--------SYVTDDYFGMLDDGEG---NS 642
gi|124008749|ref|ZP_01693438.1      ------------SRESLHPILS--------HSSDDYFGFLEASEGEWTET 528
gi|110639610|ref|YP_679820.1|       ------------SRESLKELQS--------YNSDDYFGFMDNTEGYWDET 682
gi|163754141|ref|ZP_02161264.1      ------------AFDSFNLTRG--------FMTDDFYGMMDPDEG----L 676
gi|150025454|ref|YP_001296280.      ------------SMESFSLSGS--------FVSDDYFGLMNANEG----N 662
gi|254494946|ref|ZP_01052732.2      ------------STISFNLASS--------YVTDDFFVMLDDNEG----T 522
                                                   *              .  *.::  :

gi|188993886|ref|YP_001928138.      NIGWRNYNMAVGRFPVRTPAEARIAVDKTIRYEE--DRESGAWRIRACFA 588
gi|229496468|ref|ZP_04390183.1      TNGRKQLSIGIGRFPIRTLGEAKAAVDKTIRYAE--NGDPGVWKTRTLFL 601
gi|262384061|ref|ZP_06077197.1      FSR-DKMCLGVGRFPIRTVTEATQMVDKTISYME--NKDLGSWKNNVTFV 565
gi|150009174|ref|YP_001303917.      FSR-DKMCVGVGRFPIRTVTEATQMVDKTISYME--NKDSGSWKNNVTFV 565
gi|255015781|ref|ZP_05287907.1      LDKPNSICIGVGRFPVRTVTEATQMVDKVIGYMR--NEDRGGWKNQITFV 569
gi|154494061|ref|ZP_02033381.1      SLQNKKLCLGIGRFPIRTVEQATQMVDKVISYME--NRNIGSWKNNLCFM 556
gi|218258224|ref|ZP_03474626.1      SLQNKKLCLGIGRFPIRTVEQATQMVDKVISYME--NKNTGSWKNNLCFM 556
gi|224536225|ref|ZP_03676764.1      LTA-GMLDIGIGRFPVRTAAEAKAAVDKTIAYME--NKQAGSWKHTVCYV 713
gi|189463715|ref|ZP_03012500.1      LSD-AMLDIGIGRFPVRTAAEAKATVDKTIAYMG--NKHAGPWKHTVCYV 713
gi|282881188|ref|ZP_06289875.1      KDFSDQQDIAVGRFPVTTEADAKVMVDKVIAYVT--NKDAGAWQNTLVFM 596
gi|282877309|ref|ZP_06286140.1      TNFSDKQDVAVGRFPVKTEAEAKVMVDKVINYVK--NQNGGAWQNTLVFM 608
gi|258648534|ref|ZP_05736003.1      MAQTDKLDLGIGRFMCYDAETAKMLVDKTIDYLE--NKKVGNWKNKIVMI 690
gi|124008749|ref|ZP_01693438.1      SLGDHTLEIGIGRLPVKTLEEAANVVTKLIGYANNQSN-LGSWRNRVVFV 577
gi|110639610|ref|YP_679820.1|       PLDDNTLNIGVGRLPIGSAAQGDMIVSKLINASKPSTNTNGNWKNKMTLL 732
gi|163754141|ref|ZP_02161264.1      LTISDRLDIALGRMLILDANQAKEMVDKTLTYYS--KESYGRWRNTVTIV 724
```

```
gi|150025454|ref|YP_001296280.       MLGALGLDIAVGRMLVSSLPQAEQMVSKVIDYYD--KKSFGRWRNNLVFI 710
gi|254494946|ref|ZP_01052732.2       MSTSHTVDVASSRIPVSTIAEASVVVDKILSYYS--KDAIGDWRNTITLL 570
                                             :. .*:       .   * * :          * *:

gi|188993886|ref|YP_001928138.       ADNGD-------KHATETSRLIDTVKRYAPA-IMPVRAFQDVYPHVIENG 630
gi|229496468|ref|ZP_04390183.1       ADNQDG-----YSHARQANELTEILEEIQPE-LIVNKVYLDAFPKSTVNG 645
gi|262384061|ref|ZP_06077197.1       ADDGNNEDSFTTNHMKQADQLAEAIEEVQPG-FLVNKVYFDAYKRSS--- 611
gi|150009174|ref|YP_001303917.       ADDGNNEDSFTTNHMKQADQLAEAIEEMQPG-FLVNKVYFDAYKRSS--- 611
gi|255015781|ref|ZP_05287907.1       ADDGNNADHYTSSHAEQADELARFIEENHGA-FLTNKVYFDAFKRDN--- 615
gi|154494061|ref|ZP_02033381.1       ADDGSNTDGFMTEHMEFADQLAGYVESEHPE-FLVNKLYYDAYKKDMT-- 603
gi|218258224|ref|ZP_03474626.1       ADDGSNTDGFMTEHMEFADQLAGYVESEHPE-FLVNKLYYDAYKKDMA-- 603
gi|224536225|ref|ZP_03676764.1       ADDGDK-----NLHASQSELLASYTERNYPS-LLVNRIYADAFHRESSAT 757
gi|189463715|ref|ZP_03012500.1       GDDGDG-----RLHMSQSEILASYTERNYPS-FLVERIYADAFRRESSAT 757
gi|282881188|ref|ZP_06289875.1       GDDG-----DNNLHMQDIDKTANAISSQYPS-YLVKKIMWDAYQRETSSS 640
gi|282877309|ref|ZP_06286140.1       GDDG-----EDNLHMHDINATADAISTQYPS-YLVKKIMWDAYVRETSST 652
gi|258648534|ref|ZP_05736003.1       ADNGKK--GENNLHMNDCENVIRTINAVSGDRFNLKKVYPDAYTYMTSAT 738
gi|124008749|ref|ZP_01693438.1       ADDGDV-----NIHQLDADVLAEDIRNNHKN-FNVSKLFLDAFEQVSTPN 621
gi|110639610|ref|YP_679820.1|        ADDGDG-----ITHLQAAEVLSATVNARNKQ-ININKIYVDAYQQEATPG 776
gi|163754141|ref|ZP_02161264.1       SDDAENN--SDKDLEVDLDALGNTIAQQKPF-INITKIHSDAYVQQSSAA 771
gi|150025454|ref|YP_001296280.       SDDIDKV--SDATLQTDLDDLANKITLEKPF-FNAKKIHADSYVQETTSG 757
gi|254494946|ref|ZP_01052732.2       ADDIDVV--GEEVIEQGVESIADEIKDNKPV-FNVNKIYADAFVQENSSG 617
                                     .*:               .                 :   * :

gi|188993886|ref|YP_001928138.       LHSIPGAKKKMLETLQSGIILLNYAGHGGPAGWSDEHLLTLNDIHNFN-Y 679
gi|229496468|ref|ZP_04390183.1       LTTVPTAKRKLLDAIDQGLLLVNYTGHGSPTAWTDEQIMTLPDVQRLS-N 694
gi|262384061|ref|ZP_06077197.1       LGTYPDVHNEIEKLLKSGQLLINYTGHGSTTHWADESVWTQTDINNSS-Y 660
gi|150009174|ref|YP_001303917.       LGTYPDVHNEIEKLLKSGQLLINYTGHGSTTHWADESVWTQTDINNSS-Y 660
gi|255015781|ref|ZP_05287907.1       SGTYPDVRKRISELLKKGQLLINYTGHGNTTAWSDEYVWTQTDILQST-Y 664
gi|154494061|ref|ZP_02033381.1       AGTYPDVRSGLQKLLKDGLLLFNYTGHGGTTALSDEKVLTQTDINQFT-Y 652
gi|218258224|ref|ZP_03474626.1       AGTYPDVRSGLQKLLKDGLLLFNYTGHGGTTALSDEKVLTQTDINQFT-Y 652
gi|224536225|ref|ZP_03676764.1       GETYPDATKRLLQLFNRGMLVVNYTGHGSTSAWAAENLLTMADITKMT-S 806
gi|189463715|ref|ZP_03012500.1       GETYPDATKRLLQLFDKGMLVVNYTGHGSTSAWAEENLLNMGDITQMT-S 806
gi|282881188|ref|ZP_06289875.1       GNTYPEVSRIIKQQQAAGALIMDYGGHGMATQISHEKVLNLHDFQTFQ-H 689
gi|282877309|ref|ZP_06286140.1       GHAYPEVTRLIKQQQAAGALIMDYGGHGSEIQISHEKVLNFSDFQSFN-N 701
gi|258648534|ref|ZP_05736003.1       GNSFPQVTKMLREEMNRGALIFNYTGHGSPIELSQAHLLATKDYELPT-Q 787
gi|124008749|ref|ZP_01693438.1       GELAPTLKEALTQNVEKGALIVNYTGHGGEIGWAEEKLLNVPQINDWENY 671
gi|110639610|ref|YP_679820.1|        GETASLVNEAIYRDIEDGTLIWNYVGHGGNNVLAQEAIVTTTTINSWNNI 826
gi|163754141|ref|ZP_02161264.1       GERYPEVNEAIKDNIALGSLVVNYFGHGGEDGLSGERIFEKGDSQELTNE 821
gi|150025454|ref|YP_001296280.       GQKYPKAKLDFINSFGQGALVFNYFGHGGEEGLAGERLFEAKDAMALNNK 807
gi|254494946|ref|ZP_01052732.2       GERYPEVNEAITNAIEKGTLVFDYFGHGGEDGFASERILDVSQIQSFNNL 667
                                         .     :      * :: :* ***     :   :

gi|188993886|ref|YP_001928138.       KHMPIWITATCDFANYDSQTT-SAGEEVFLHEKSGTPIMFSTTRVVYNTQ 728
gi|229496468|ref|ZP_04390183.1       KRLPLWITATCDFCPYDNSTT-SAGEVAFLNDRGGASALFTTTRVVFDVP 743
gi|262384061|ref|ZP_06077197.1       KHLPVWVTATCDFTRFDDVKT-SAGESVFLNPTSGGIALFTTTRVVFSGN 709
gi|150009174|ref|YP_001303917.       KHLPVWVTATCDFTRFDDVKT-SAGESVFLNPTSGGIALFTTTRVVFSGN 709
gi|255015781|ref|ZP_05287907.1       TKLPIWVTATCDFTRFDDVNT-SAGESVFLNAKSGGIALFTTTRVVVSSG 713
gi|154494061|ref|ZP_02033381.1       THLPVWVTATCDFTRFDDLNT-SAGEDVFLNKSSGGIALFTTVRVAYSRP 701
gi|218258224|ref|ZP_03474626.1       THLPVWVTATCDFTRFDDLNT-SAGEDVFLNKSSGGIALFTTVRVAYSRP 701
gi|224536225|ref|ZP_03676764.1       PRLPLWITATCDFTRFDDIQT-SAGEQAFLNTKGGAIALLTTSRVVYASQ 855
gi|189463715|ref|ZP_03012500.1       PRLPLWITATCDFTRFDDIQT-SAGEQAFLNAKGGAIALLTTSRVVYAAQ 855
gi|282881188|ref|ZP_06289875.1       PHLPLWITASCDIMPFDGETP-TIGEVAVRNPRGGAISFFGTTRTVIAHY 738
gi|282877309|ref|ZP_06286140.1       PNLPLWVTASCDIMPFDGVVP-TIGEAAVLNPKGGALAFFGTTRTVWAYY 750
gi|258648534|ref|ZP_05736003.1       GNLPLWIFASCEVTPFDQQTN-DIGRAALYNKNGGAMAVICASRSVFANY 836
gi|124008749|ref|ZP_01693438.1       HNLPLFVTATCEFGRYDDPLRVAGAEYILLNPKGGGIGLITTTRPVYSST 721
gi|110639610|ref|YP_679820.1|        NKLPFFITATCSFGRFDKPGLVSGAENVLMSSNGGSFGNLTSTRTVYSFS 876
gi|163754141|ref|ZP_02161264.1       CRLPLFITITCEYTRFDNPLRETAGEFMFWNENGGAVALLTTTRQIFQNV 871
gi|150025454|ref|YP_001296280.       YKYPLFITVTCEFTRFDNPSRPTAGEQTYWNPLGGAVSMITTTRQIGQST 857
gi|254494946|ref|ZP_01052732.2       NTLPLLITVTCDFSRFDNPNRITAGEITFKKSDGGAASMITTTREVFIST 717
                                        *. :  :*.   :*       ..       .*    : : *

gi|188993886|ref|YP_001928138.       NEKINGFMLRRMFEKAKDGRYRTMGEIIRSAKQGM---LSTVFP----DS 771
gi|229496468|ref|ZP_04390183.1       NQELNRHFLRTLFTQDKGGRLHQLGDVLRNAKN------SAYTS----DT 783
gi|262384061|ref|ZP_06077197.1       NANLNKALIDNLFQEDANSRY-TLGEAMMYTKRQL---NDS--------- 746
gi|150009174|ref|YP_001303917.       NANLNKALIDNLFQEGANSRY-TLGEAMMYTKRQL---NDS--------- 746
gi|255015781|ref|ZP_05287907.1       NSALNKELYRNLFERESDGRARTLGEAMMETKRKL---SGI--------- 751
gi|154494061|ref|ZP_02033381.1       NFPINDNVIRNLFERN-NGRRRTLGEVMQATKNTL---SSV--------- 738
gi|218258224|ref|ZP_03474626.1       NFPINDNVIRNLFERN-NGRRRTLGEVMQATKNTL---SSV--------- 738
gi|224536225|ref|ZP_03676764.1       NSTLNQAFLRHIFSRP-EGKRLRLGDIMRLSKCDE---SLAN-------D 894
gi|189463715|ref|ZP_03012500.1       NSTLNQAFLRHIFSRP-EGKRLRLGDVMRLSKCDA---SLAT-------D 894
gi|282881188|ref|ZP_06289875.1       NARINTSFLRYVLSKDAHGNPVTIGEAQRLAKNEM---IT----SRKDLT 781
gi|282877309|ref|ZP_06286140.1       NAQINTSFLRHVLSKDRAGKPITIGEAQRLAKNEM---ID----TKRDLT 793
gi|258648534|ref|ZP_05736003.1       NRSLNVVLNKNLLSMNQDGAYNTMGEAMRLTKIGL---LDGSYNKETDRS 883
```

```
gi|124008749|ref|ZP_01693438.1      NFLLSKAFYAEVFKP-IDGAMPRLGDLMIKTKNNS--L----------NG 758
gi|110639610|ref|YP_679820.1|       NLAINNAFYSYAFLTNADGSYYTLGDIMKNTKNNAAAL----------QG 916
gi|163754141|ref|ZP_02161264.1      GVSINELLARYLFSYGSN-EYQPVSEALRQTKTLV-------------TT 907
gi|150025454|ref|YP_001296280.      GEIFNVNLASKLFAYGSE-NYTSVAESVRLTKVMS-------------AD 893
gi|254494946|ref|ZP_01052732.2      GQRFNEQLIRVLLSFNGE-DYS-IAEALATTKNQF-------------NS 752
                                    .  :.  .    :          :.:    :*

gi|188993886|ref|YP_001928138.      INQLSFFLMGDPSVRMNLPTHKVQLTAINGQDPEGQYGT-IMLKSLERVA 820
gi|229496468|ref|ZP_04390183.1      INKLNFSLIGDPALRLKMPTHQAALLKINGKQPDPQKG--ISLHALERVS 831
gi|262384061|ref|ZP_06077197.1      -NKLNFILIGDPALKFAYPEYKARVTAVNGEAVSDEP---FEFKALSRIT 792
gi|150009174|ref|YP_001303917.      -NKLNFILIGDPALKFAYPEYKARVTAVNGEAVSDEP---FEFKALSRIT 792
gi|255015781|ref|ZP_05287907.1      -NKLNFILIGDPALRISYPEYKAQVTAVNGKAISDEP---FTFKALEKIT 797
gi|154494061|ref|ZP_02033381.1      -YKLGFCLIGDPAVKIAYPEFGMKVTTVNGQSVDGNS---ISFKALEKIT 784
gi|218258224|ref|ZP_03474626.1      -YKLGFCLIGDPAVKMAYPEFGMKVTTVNGQAVDENS---ISFKALEKIT 784
gi|224536225|ref|ZP_03676764.1      RNKLNFSLIGDPALTLAYPDYQVQVDEFAGVNVAEETSVYPQVKAGSKIT 944
gi|189463715|ref|ZP_03012500.1      KNKLNFSLIGDPALTLAYPDYQIQVDEFAGVNVAEETNTYPQVKAGSKIT 944
gi|282881188|ref|ZP_06289875.1      CNKLQYALLGDPAVALHQPSYNIVVDSINGKALASTPT--VALQAGSTVT 829
gi|282877309|ref|ZP_06286140.1      DNKLQYSLLGDPAVALHQPTLKVVVDSINGSPVSTDVK--ASLKAGGIAK 841
gi|258648534|ref|ZP_05736003.1      MNKLKYTLLGDPALPLMAPRQEIVLDSINGAPINSSSK--IQLKAGEKVR 931
gi|124008749|ref|ZP_01693438.1      AINRNFALLGDPSLRLAYPKHKVAVTKITNANTGQVVN---TISALDKVT 805
gi|110639610|ref|YP_679820.1|       VYNRNYTLLGDPSMKISYPQKKMIIIAVNGTPVNSVPD---TLKALSKVT 963
gi|163754141|ref|ZP_02161264.1      TNKRTVFYIGDPALKLAIPRPRVNLTAINDVPITQPTD---TLKALSKVK 954
gi|150025454|ref|YP_001296280.      SGNNVISYIGDPALKLAIPKPKVKLTKINGVPITGTPD---VLQALGYVT 940
gi|254494946|ref|ZP_01052732.2      TQKFFIYSFGDPAMKLAIPKPNVRITKMNDVPITQSLD---TIKALSKIK 799
                                      :     :***:: :  *     :  . .            . :

gi|188993886|ref|YP_001928138.      LKGKVTDEKGTFDETFSGKVFLTVFDGRKKMTALEE---EGN-DL-SLVY 865
gi|229496468|ref|ZP_04390183.1      LEGAIHDLSGTIDGNFNGTIAITVFDAEQISSTLKENIPEYQ-ES-VYKF 879
gi|262384061|ref|ZP_06077197.1      VEGEILNPSGSFAADFTGVLSSTIFDSQSSITTLGNSSE-------KFTY 835
gi|150009174|ref|YP_001303917.      VEGEILNPSGSFAADFTGVLSSTIFDSQSSITTLGNSSE-------KFTY 835
gi|255015781|ref|ZP_05287907.1      VEGEILDTKEGLANDFTGILNATVLDSKASLTTLGNNTNEKG-DTVRFSY 846
gi|154494061|ref|ZP_02033381.1      VEGEVLDASGQLVTDFTGIVNPTVKDSKVTVTCLKNSNKD---DSPAFTF 831
gi|218258224|ref|ZP_03474626.1      VEGEVLDASGQLVTDFTGIVNPTVKDSKVTVTCLKNSNKD---DTPAFTF 831
gi|224536225|ref|ZP_03676764.1      VKGRILTPEGALAEDFTGTVHPTVLDSKEEVTTLDNRDEG------AFTY 988
gi|189463715|ref|ZP_03012500.1      VKGRILTPEGALAEDFTGTVHPTVLDSKEEVTTLDNRGEG------AYTY 988
gi|282881188|ref|ZP_06289875.1      VKGHIEG-----VSDFQGLVFATVRDNEEKIVCRRNDQKE---TKEALTF 871
gi|282877309|ref|ZP_06286140.1      VTGHVEH-----AADFNGKVTVTVRDSQEKIVCRMNDAAD---ITSPFTY 883
gi|258648534|ref|ZP_05736003.1      FSGHVIKNN-AVDTGFSGVVTGSLHDRKETITCK-NNGGN---ASEAMVY 976
gi|124008749|ref|ZP_01693438.1      VEGEVTNAQDILLDGFEGVLDITIFDKAQELTTLG-TESSR------IQF 848
gi|110639610|ref|YP_679820.1|       LDGLVQDYTGTTLTSYSGVSRITIFDKPTTVPTLGSTGSSK------TTF 1007
gi|163754141|ref|ZP_02161264.1      MAGNITDEFGNLLDTYNGTVFSTIYDKKIQRQTLANDFP------FVLDF 998
gi|150025454|ref|YP_001296280.      LTGEVTDQFDAPLTSYNGELAINIYDKNINRTTLANDAISLNGVLQIMNF 990
gi|254494946|ref|ZP_01052732.2      FEGVVTDDTNSVLTNYNGTLSTTVFDKVIEKNTLDNDGFG-----VTMVF 844
                                    . * :          : *    .: *                       :

gi|188993886|ref|YP_001928138.      YDYPNVMYAGIAEVKDGLFETSFIVPKDVNYSEHEGRINLYAYNESTKAE 915
gi|229496468|ref|ZP_04390183.1      SEYPGIIYAGNAEVVNGQFTASFVVPKDISYSDKKGKINFYAYSSSLKRE 929
gi|262384061|ref|ZP_06077197.1      LDYPNTIYIGRDSVRNGKFSFTFMVPKDISYSNKKGKLNLYASSET--KE 883
gi|150009174|ref|YP_001303917.      LDYPNTIYIGRDSVRNGKFSFTFMVPKDISYSNKKGKLNLYASSET--KE 883
gi|255015781|ref|ZP_05287907.1      TDYPNTIYIGQDSVRQGKFSFTFMVPKDISYSNKQGKLNLYASDEVNKHE 896
gi|154494061|ref|ZP_02033381.1      TDYPNTIFIGNDSVRNGKFSFTFTVPKDISYSNLQGKMNLYAVDTESGNE 881
gi|218258224|ref|ZP_03474626.1      TDYPNTIFIGNDSVRNGKFSFTFTVPKDISYSNLQGKMNLYAVDTENGHE 881
gi|224536225|ref|ZP_03676764.1      TERSKTLFSGSDSVRQGRFEFTFPVPLDINYSDEEGLLSLYALDAVHSHE 1038
gi|189463715|ref|ZP_03012500.1      TERSKTLFSGSDSVRQGRFEFTFPVPLDINYSDEEGLLSLYALDAVHSNE 1038
gi|282881188|ref|ZP_06289875.1      MDRPRTLYNGANKVENGKFSFVFAVPSDINYSEGSGKLNLYAISDNHQMR 921
gi|282877309|ref|ZP_06286140.1      YDRTKTLFNGSGNVVGGRFTLTFAIPQDINYTDGTGLMNLYALSDDHRLK 933
gi|258648534|ref|ZP_05736003.1      TDWPNSLYEGSDSVKQGCFTLNITIPHEISYSNQNGRLSLYAVDNTRQEE 1026
gi|124008749|ref|ZP_01693438.1      KAQNNKIFVGKASVKAGKFTFTFIVPKDIDYKFGEGKWSFYAQANDGVND 898
gi|110639610|ref|YP_679820.1|       KLQNNIIFDGLVTVTNGAYKVSFIVPKDISYNFDYGKISLYSELTNGTTD 1057
gi|163754141|ref|ZP_02161264.1      ETLGEIIFRGKSEVVDGNFEFEFVVPRDITIPVGNGRISFYAEKGDILED 1048
gi|150025454|ref|YP_001296280.      TTLGETIFRGNASVTNGQFEFSFVVPRDIKIPVGNGKISFYAKNNNALED 1040
gi|254494946|ref|ZP_01052732.2      DTQDSKLFRGKATVENGVFSFDFVVPKDIKIAYGKSKLSFYAEDG--EVE 892
                                          :: *   *  * :   : :* ::      .  .:*:

gi|188993886|ref|YP_001928138.      AMGVDFSIRVQPGIPDEVTEDNTPPEIISCFLNDSTFRSGDEVNPTPLFM 965
gi|229496468|ref|ZP_04390183.1      AMGVDKSLKLEMGG-SGSEEDKTPPEIQRIYLNDSTLRAPIVVGTTPLFV 978
gi|262384061|ref|ZP_06077197.1      AQGSFFDFIVGGTSD-TAETDTIGPEIRQIYLNDSSFVSGDKVNTTPYFV 932
gi|150009174|ref|YP_001303917.      AQGSFFDFIVGGTSD-TAETDTIGPEIRQIYLNDSSFVSGDKVNTTPYFV 932
gi|255015781|ref|ZP_05287907.1      AQGSFLNYIVGGTAD-NAEADTIGPEIRQIYLNDSSFVEGDQVNTTPYFV 945
gi|154494061|ref|ZP_02033381.1      AQGNFDNFIVGGTSD-TAETDTIGPEIRALYLNDTTFVDGGQVNTTPYFV 930
gi|218258224|ref|ZP_03474626.1      AQGNFDNFIVGGTSD-TAETDTIGPEIRALYLNDTTFVDGGQVNTTPYFV 930
gi|224536225|ref|ZP_03676764.1      AGGAFDRFLVGGTDDGVSLTDTLGPKI-TVYLNTPDFSPGGQTNTTPLFV 1087
gi|189463715|ref|ZP_03012500.1      AGGAFDRFLVGGTDDDVSLADTLGPKI-TIYLNTPDFSSGGQTNTTPLFV 1087
gi|282881188|ref|ZP_06289875.1      ASGVSEAFTLGGGAE-LTT-DSLGPSI-YCYLNSPSFVNGGNVNSTPYFV 968
gi|282877309|ref|ZP_06286140.1      AHGTSEAFTIGGTAD-VAN-DSIGPSI-YCYLNSPSFTNGDKVNSTPYFV 980
gi|258648534|ref|ZP_05736003.1      AHGFNENFFMNGSAT-TTETDSIGPKI-FIYLNTPDFPNGGYVKSDALLI 1074
```

```
gi|124008749|ref|ZP_01693438.1      AQGAKTDVIIGGTSA--SITPDDTPPAITLFMNDETFVNGGVVQPSATLI 946
gi|110639610|ref|YP_679820.1|       AGGYLSNIVVGGSNA--NAPEDKIPPTVKLYLNDPSFVSGGVSRENPVFY 1105
gi|163754141|ref|ZP_02161264.1      HTGFNESFKVGELDE--NAPVDNDPPVVNLFLNDESFVSGGITNESPFLL 1096
gi|150025454|ref|YP_001296280.      QRGHDFSIKIGGLNT--NAVADVVPPKIKIYMNDTSFVSGGITNQSPLFL 1088
gi|254494946|ref|ZP_01052732.2      KAGANFDVTVGGINE--NAPEDTIGPEISLFMNDESFIDGGNTNASPNLI 940
                                      *      :                    ::*   :         . :

gi|188993886|ref|YP_001928138.      AEVFDLNGINITGSGVGHDITLCIDGRADLTYNLNAYFTSSATDAGVGTI 1015
gi|229496468|ref|ZP_04390183.1      ADLFDMSGINLSAGGLGHGITLSIDNNPTYTFTLNDYYRANQLEAGKGSV 1028
gi|262384061|ref|ZP_06077197.1      AKLWDKSGVNITGSSVGHDMMLTIDSMPSMSYNLNSYYALLPDSENEGLV 982
gi|150009174|ref|YP_001303917.      AKLWDKSGVNITGSSVGHDMMLTIDSMPSMSYNLNSYYALLPDSENEGLV 982
gi|255015781|ref|ZP_05287907.1      ARLWDKSGVNITGSSVGHDIMLTVDSMPSMSYNLNNYYALLPDKEGEGLV 995
gi|154494061|ref|ZP_02033381.1      AELWDKSGVNITGSSVGHDMMLVIDESTVLSYNLNSYYELLPGEDGTGIV 980
gi|218258224|ref|ZP_03474626.1      AELWDKSGVNITGSSVGHDMMLVIDESTVLSYNLNSYYELLPGEEGAGIV 980
gi|224536225|ref|ZP_03676764.1      AELEDADGINTVGNGIGHDLSLCIDGSAVLTYNLNDYYTPVAGDYTRGTV 1137
gi|189463715|ref|ZP_03012500.1      AELEDADGINTVGNGIGHDLSLSIDGSAVSTYNLNDYYTPVAGDYTRGTV 1137
gi|282881188|ref|ZP_06289875.1      AEIKDDDGINVSGSGIGHDLQLIIDGDMSKSYNLNANFTYDFGSYTAGST 1018
gi|282877309|ref|ZP_06286140.1      AKITDENGINSAGSGIGHDLQLIIDGEMSKTYNLNDHFVYDFGSYTTGTT 1030
gi|258648534|ref|ZP_05736003.1      ATLSDSAGINTVKNSVGHALELVLDNQQGIPILLNDYFSYDFGSSTSGTV 1124
gi|124008749|ref|ZP_01693438.1      AKLSDENGLNIAQTGVGHEMTAVLDGDLENPWILNDFYEGNLDTYQEGVV 996
gi|110639610|ref|YP_679820.1|       ADVADENGINVSAAGIGHEITLVLSNNSE-VIILNKYYTASKDDYSKGKV 1154
gi|163754141|ref|ZP_02161264.1      AKLSDENGINT-ASGIGHDIVAILDGDEVNPFVLNDYYETELNDYTRGIV 1145
gi|150025454|ref|YP_001296280.      AFLEDEHGINT-ASGIGHDIVVYLDGDEAKPYVLNDFYETELNNYTKGKL 1137
gi|254494946|ref|ZP_01052732.2      AVLSDANGINTSITAVDHDIVAILDGDTANPIILNDFYQTELNDFTTGKV 990
                                    * : *  *:*    .:.* :   :.        **  :         *

gi|188993886|ref|YP_001928138.      LFMIPALAEGDHTARLTVWDIFNNAVHHDFSFRVVDGIAPDVADVILFPN 1065
gi|229496468|ref|ZP_04390183.1      VYLLPTLPEGDHTATLRVWDVFNNAAEKSISFRVNKDLAPQVAASQAFPN 1078
gi|262384061|ref|ZP_06077197.1      QFSIPELEPGMHTAEFKVWDILNNSTTYIFTFEVAEGLKPNLIEMYATPN 1032
gi|150009174|ref|YP_001303917.      QFSIPELEPGMHTAEFKVWDILNNSTTYTFTFEVAEGLKPNLIEMYATPN 1032
gi|255015781|ref|ZP_05287907.1      QFSIPELEPGMHTAEFKVWDILNNSTTYTFTFEVAEGLKPNLIEMYATPN 1045
gi|154494061|ref|ZP_02033381.1      KFPIPALEPGKHTAEFWVWDILNNSTVRTFTFEVVEGLKPFLFDVIATPG 1030
gi|218258224|ref|ZP_03474626.1      KFPIPALEPGKHTAEFWVWDIQNNSTVRTFTFEVVEGLKPFLFDVIATPG 1030
gi|224536225|ref|ZP_03676764.1      YFSVSELTEGKHTLSFRAWDLLNNSSTKTLEFEVVRGLRPGLFSVICTQS 1187
gi|189463715|ref|ZP_03012500.1      HFSVPELTEGKHILSFRAWDLLNSSSTKTLEFEVVRGLRPGLFSVTCTQS 1187
gi|282881188|ref|ZP_06289875.1      FYQLPELAPGPHQLLFRAWDVLNNPSTTKLSFNVVRGMEPQLFSIDCTNN 1068
gi|282877309|ref|ZP_06286140.1      HYNIPELAPGPHKLQFRAWDVLNNSSTAELTFHVVKGMQPNLFSVDCTNN 1080
gi|258648534|ref|ZP_05736003.1      TYPLEGLTNGKHRLSLRAWDINDNSTTAYLDFIVSEQAG-KEMDVNATQN 1173
gi|124008749|ref|ZP_01693438.1      QYPVRDLAKGKHTVVVKVWDTHNNSSEASIDFVVTDNA-VMAINEVMNYP 1045
gi|110639610|ref|YP_679820.1|       EFPLKDLSPGTYSLRFKVWDTYNNSTEETLEFTIEATS-KIQLSHVLNYP 1203
gi|163754141|ref|ZP_02161264.1      KFPLRDLEDGTHTLSLKAWDVYNNSSTTEIQFTVFNENNSLTITNVLNYP 1195
gi|150025454|ref|YP_001296280.      KYPFRNLAIGLHTLTFKAWDVYNNIATASLQFVVVGDE-GLALTNVLNYP 1186
gi|254494946|ref|ZP_01052732.2      NYTLRDLETGPHTLKIKAWDTYNNSSETTLNFVVVSDA-ILNLENVLNYP 1039
                                     : .  *  * :   . .**  :.     : * :

gi|188993886|ref|YP_001928138.      P--VRESATFRIFHNRPGSDLNVVVEIYDFTGRLVNSL-----PVKTYSS 1108
gi|229496468|ref|ZP_04390183.1      PALLGSPITFEIFTNTPGEEMSLTIELFDFTGRCVAKS-----PSFSLVS 1123
gi|262384061|ref|ZP_06077197.1      P--ARDQVEFFLHHNRPESNLKVTVMVYDMTGKFLWS------TEKSGSS 1074
gi|150009174|ref|YP_001303917.      P--ARDQVEFFLHHNRPESNLKVTVMVYDMTGKFLWS------TEKSGSS 1074
gi|255015781|ref|ZP_05287907.1      P--ARDQVEFFLHHNRPESNLKVTVMVYDMTGKFLWS------TEKSGSS 1087
gi|154494061|ref|ZP_02033381.1      I--AREQVTFHLMHNRPESRMRVGIMVYDLAGRQLWK------HEESGTS 1072
gi|218258224|ref|ZP_03474626.1      I--AREQVTFHLMHNRPESRMRVGIMVYDLAGRQLWK------HEESGTS 1072
gi|224536225|ref|ZP_03676764.1      P--ARESTTFVLSHNRPGSTLAVRMSVYDFAGREMWT------HLEKGVS 1229
gi|189463715|ref|ZP_03012500.1      P--ARESTTFILSHNRPGSTLSVRMSVYDFAGREMWT------HMEKGIS 1229
gi|282881188|ref|ZP_06289875.1      P--AREATTFILEHDRAGAVVDVEIEVFDLSGRLLWH------HQES-SV 1109
gi|282877309|ref|ZP_06286140.1      P--ARTTTTFIINHDRVGAQVDVEIDVFDMSGRQLWK------HQER-GV 1121
gi|258648534|ref|ZP_05736003.1      P--AKNNTLFITTIASDQAEQPVTTEVYDVSGRLVWS------ASAK-VS 1214
gi|124008749|ref|ZP_01693438.1      N-PFNQATTFRFDHNRAGEALEVTIQIYNRNGQHVKK------LQYTTSK 1088
gi|110639610|ref|YP_679820.1|       N-PFTTNTTFHFDHNRFGDNLMVQVQIYTVSGKLVKT------LDETIFN 1246
gi|163754141|ref|ZP_02161264.1      N-PFVSYTEFWFNHNSSSE-LDIMVQVFTISGKVVRTLVGKTNAGATSKD 1243
gi|150025454|ref|YP_001296280.      N-PFVNHTEFWFTHNRPFEPLEVQIQVFTITGKIVWT----KNQIVSNEG 1231
gi|254494946|ref|ZP_01052732.2      N-PFVNYTEFWFNHNKPNEPLEVQVQIFTVSGKLVKT----INRNVQTTG 1084
                                             *            :   ::   *: :

gi|188993886|ref|YP_001928138.      SYGEPIEIKWDLTSKYGVKIGNGFYLYRCVVNSPGGQTASMAKKMIVVAQ 1158
gi|229496468|ref|ZP_04390183.1      PSQGSIRIPWTPTTSYQTSPIRGLYIYRCTLSGSNGKSATTMGKLLLEES 1173
gi|262384061|ref|ZP_06077197.1      ELFKAYIVTWNLTDNGGRRLRPGVYLYRAAISTNNSKEATKANKLIILAQ 1124
gi|150009174|ref|YP_001303917.      ELFKAYIVTWNLTDNGGRRLRPGVYLYRAAISTNNSKEATKANKLIILAQ 1124
gi|255015781|ref|ZP_05287907.1      ELFKAYIVTWNLTDNGGRRLRPGVYLYRAAISTNNSKEATKANKLIILAQ 1137
gi|154494061|ref|ZP_02033381.1      GLFENYTVSWDLT-SGGARMRPGVYIYRAAISTDNSKDATKARKFIILGE 1121
gi|218258224|ref|ZP_03474626.1      GLFENYTVSWDLT-CGGARMRPGVYIYRAAISTDNSKDATKARKFIILGE 1121
gi|224536225|ref|ZP_03676764.1      EG-QTYYVEWDLCSNGGQRLAPGVYLYRASIVSDGSRESTKSQKIIILSQ 1278
gi|189463715|ref|ZP_03012500.1      EG-QTYYVEWDLCSNGGQRLAPGVYLYRASIVSDGSRESTKSQKIIILSQ 1278
gi|282881188|ref|ZP_06289875.1      STTNTQTVNWNLTVENGNRLQTGVYLYRARISSEGSAKVSKAKKLIVVSN 1159
gi|282877309|ref|ZP_06286140.1      STNDAYTVDWDLIVDNGARLQTGVYLYRVSLSSEGSQKVSKAKKLIVIGN 1171
gi|258648534|ref|ZP_05736003.1      GT--YATINWDLTNSRGAKVNAGIYIYRSLVNG----KHTKAKKMIVLKQ 1258
```

```
gi|124008749|ref|ZP_01693438.1      ALTRFEG-VWDGLDKQGKKIPQGVYVYKITARSVTDGAEEFVVRRLVVLD 1137
gi|110639610|ref|YP_679820.1|       SPSHVANLTWDGLDDYGDKIGRGVYVYKLKIRSLQDGSTTHVFEKLVLLN 1296
gi|163754141|ref|ZP_02161264.1      FSSLSRDIVWDGKDDFGDKLAKGVYVYKITVKSPLTNQKVEKFEKLVIL- 1292
gi|150025454|ref|YP_001296280.      FT--SRDITWDGRDDFGDKIGKGVYVYKLTVKSNLTNKKIEKFEKLVIL- 1278
gi|254494946|ref|ZP_01052732.2      NL--SRNITWNGLDDFGNKIGKGVYVYKLTVKATASNLVSEKYEKLVILQ 1132
                                             *            *.*:*:               . ::

gi|188993886|ref|YP_001928138.      ------
gi|229496468|ref|ZP_04390183.1      SSGATK 1179
gi|262384061|ref|ZP_06077197.1      ------
gi|150009174|ref|YP_001303917.      ------
gi|255015781|ref|ZP_05287907.1      ------
gi|154494061|ref|ZP_02033381.1      ------
gi|218258224|ref|ZP_03474626.1      ------
gi|224536225|ref|ZP_03676764.1      ------
gi|189463715|ref|ZP_03012500.1      ------
gi|282881188|ref|ZP_06289875.1      N----- 1160
gi|282877309|ref|ZP_06286140.1      N----- 1172
gi|258648534|ref|ZP_05736003.1      ------
gi|124008749|ref|ZP_01693438.1      ------
gi|110639610|ref|YP_679820.1|       ------
gi|163754141|ref|ZP_02161264.1      ------
gi|150025454|ref|YP_001296280.      ------
gi|254494946|ref|ZP_01052732.2      ------
```
